# Gray-Horse Melanoma—A Wolf in Sheep’s Clothing

**DOI:** 10.3390/ijms26146620

**Published:** 2025-07-10

**Authors:** Daniela M. Brodesser, Karin Schlangen, Alexandro Rodríguez-Rojas, Benno Kuropka, Pavlos G. Doulidis, Sabine Brandt, Barbara Pratscher

**Affiliations:** 1 Research Group Oncology (RGO), Centre for Equine Health and Research, Department for Small Animals and Horses, University of Veterinary Medicine, Veterinaerplatz 1, 1210 Vienna, Austria; daniela.brodesser@vetmeduni.ac.at; 2 Section for Biosimulation and Bioinformatics, Centre for Medical Statistics, Informatics and Intelligent Systems, Medical University of Vienna (MUV), Waehringer Guertel 18-20, 1090 Vienna, Austria; karinschlangen@gmx.net; 3 Division of Small Animal Internal Medicine, Department for Small Animals and Horses, University of Veterinary Medicine, Veterinaerplatz 1, 1210 Vienna, Austria; alexandro.rojas@vetmeduni.ac.at (A.R.-R.); pavlos.doulidis@vetmeduni.ac.at (P.G.D.); barbara.pratscher@vetmeduni.ac.at (B.P.); 4 Institute of Chemistry and Biochemistry, Freie Universität Berlin, 14195 Berlin, Germany

**Keywords:** melanoma, gray-horse melanoma, tumor tissue, primary melanoma cells, RNAseq, proteomics

## Abstract

Malignant melanoma (MM) affects not only humans but also animals, with gray horses being particularly predisposed to acquiring the disease. Multiomics have greatly advanced the understanding of human MM. In contrasty little is known regarding the pathogenesis of gray-horse melanoma and the unique phenomenon of melanoma “dormancy” in some animals. To help close this gap in knowledge, melanoma tissue and intact skin collected from gray horses were subjected to transcriptome analysis using RNAseq. In the next step, cultured primary tumor cells and normal skin fibroblasts were established from gray horses, and their protein expression profiles were determined. The obtained data unambiguously identified gray-horse melanoma (ghM) as a malignant tumor, as reflected by the overrepresentation of pathways typically activated in human melanoma and other human cancers. These included the RAS/RAF/MAPK, the IRS/IGF1R, and the PI3K/AKT signaling networks. In addition, the obtained data suggest that the key molecules RAC1, RAS, and BRAF, which are frequently mutated in human melanoma, may also contain activating mutations in ghM, whilst PTEN may harbor loss-of-function mutations. This issue will be subject to downstream analyses determining the mutational status in ghM to further advance the understanding of this frequent disease in gray horses.

## 1. Introduction

Malignant melanoma (MM) is a dreaded cancer disease that arises from melanocytes. In humans, MMs develop in response to genetic aberrations that are induced by ultraviolet radiation (UVR) and/or other environmental and intrinsic factors, including a genetic predisposition [1]. Melanoma is not confined to humans. It also affects various animal species, including mammals, birds, and fish. Research in rodent and zebrafish models, as well as MM-susceptible animal species such as Xiphophorus hybrid fish, miniature pigs, and several canine breeds, has considerably helped advance the understanding of the mechanisms underlying MM development [2,3,4].

The highest incidence of melanoma is probably observed in horses with a gray coat color (i.e., white). It is estimated that about 80% of gray horses older than 15 years of age are affected by these tumors, while non-gray horses only rarely develop the disease [5,6,7]. Genetic studies have led to the identification of a 4.6 kbp duplication within Intron 6 of the Syntaxin 17 gene that is responsible for both the gray phenotype and the high susceptibility to melanoma [8]. In addition, it was shown that horses homozygous for the Intron 6 duplication (G/G; with G = gray) are at higher risk of developing more aggressive melanoma at a younger age than heterozygous individuals (G/g) [8,9,10].

**Figure 1:**
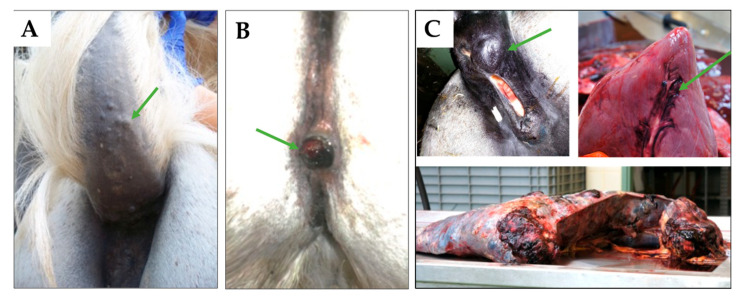
Clinical presentation of ghM. (**A**) GhMs typically develop under the tail-root and present as firm, encapsulated nodules (green arrow), as observed in a Shagya–Arabian mare (GYN). (**B**) Surgical therapy bears the risk of tumor recurrence. Excision of anogenital lesions affecting a gray Icelandic horse (DOL) resulted in the reoccurrence of ghM at the same site (black nodular mass indicated by the green arrow). (**C**) Metastatic disease in a Trakehner cross-breed (ELL). The mare presented with a large perianal lesion (black nodular mass; green arrow) and was euthanized due to acute hemorrhage from the ruptured spleen. Necropsy revealed multiple organ metastases, involving, e.g., the interventricular sulcus of the heart (black region; green arrow), the liver, and the entire spleen (bottom).

The malignancy of gray-horse melanoma (ghM) is still a matter of discussion. The observation that the tumors are frequently encapsulated and remain quiescent for many years has led to the clinical classification of gray-horse melanomas as a “benign” tumor disease by some [11]. However, several aspects refute this theory. As a matter of fact, a considerable proportion of gray horses—notably those with the G/G phenotype—develop multiple melanomas in their early adulthood that progress to metastasizing and eventually lethal disease [10,11,12]. In addition, two-thirds of individuals affected by ghM were shown to display metastatic disease at necropsy irrespective of the clinical picture [5]. Examples of the clinical appearance of ghM are given in Figure 1.

Innovative multiomics technologies—especially when applied on a single-cell level—have pronouncedly advanced the understanding of human MM (hMM) onset and progression [4,13]. It is well accepted today that hMM is a highly heterogeneous tumor composed of different cancer cell phenotypes. Using a biological program termed epithelial–mesenchymal transition (EMT), hyperproliferative but hypo-invasive epithelial cancer cells can reversibly acquire a mesenchymal phenotype endowed with the capacity to detach from the primary lesion and migrate to and enter the vasculature [14,15,16,17,18]. In addition, melanoma cells can switch to a multi-drug-resistant stem cell-like phenotype that has the ability to self-renew and re-differentiate [19,20]. Intriguingly, these cancer stem cells (CSCs) can adopt an endothelial phenotype that can build an alternative network of pseudo-vessels, which is termed vasculogenic mimicry (VM). VM was first demonstrated for hMM by Maniotis and colleagues [21] in vitro, and it was subsequently confirmed in vivo [22,23,24]. There is robust evidence that VM assures hMM perfusion and promotes metastasis. Consequently, the presence of VM is associated with invasive disease and poor prognosis [25].

Not only have multiomics helped uncover the high plasticity of tumor cells, but the technology has also allowed the dissection of a tumor’s microenvironment and identify protumoral cells, factors, and molecules, as well as important mechanisms of immune evasion driving hMM development and metastasis [13]. These considerable advances fueled the establishment of more effective and personalized therapies, which led to a decrease in hMM-associated mortality rates within the last decade [26].

Whilst a wealth of multiomics data are available for hMM, information on the differential gene expression in equine and, notably, gray-horse melanoma is still lacking. To improve today’s understanding of this common disease on a molecular-biological level, a pilot transcriptome study of ghM versus intact skin was conducted using RNAseq. Subsequently, protein isolates from primary cell cultures established from tumor and skin samples were subjected to quantitative proteomics analysis. In sum, the obtained data revealed the deregulation of important biological pathways in ghM compared to normal skin and underscored the malignant potential of melanoma in gray horses, thus unmasking the disease as “a wolf in sheep’s clothing”.

## 2. Results

### 2.1. RNAseq Reveals Deregulated Gene Transcription That Notably Affects ECM-Associated Pathways in ghM

RNAseq analysis led to the identification of 765 genes with upregulated transcription and 1175 genes with downregulated transcription in ghM compared to normal skin (*p* < 0.001; FDR < 0.001; ≥2-fold deregulation of transcription). These data are provided in Supplementary Files S1 and S2 (“File S1-RNAseq Upregulated”, “File S2-RNAseq Downregulated”).

Notably, deregulation events affected, inter alia, the transcription of genes commonly used as diagnostic MM markers in human medicine [27], as shown in Table 1.

In order to determine the effect of transcriptional deregulation events on biological pathways in ghM, RNAseq data (Supplementary Files S1 and S2) were subjected to pathway analysis. Overall, 1339 out of 1940 submitted identifiers were found via Reactome, where 1941 pathways were hit by at least one of them. Table 2 presents the biological pathways that were most significantly (*p* ≤ 0.002) enriched in ghM compared to normal skin.

Table 2 reflects that the deregulation of gene transcription notably affected GTPase activity and signaling in the tumor samples. Reactome analysis detected the RAC1, RGO, RHOA, and CDC42 cycles as overrepresented pathways. A marked enrichment was also observed for pathways associated with ECM organization/integrity, as well as with cell adhesion, polarity, and migration (Table 2).

### 2.2. Proteome Analysis Unambiguously Identifies ghM as a Malignant Disease

Following pilot transcriptomics from ghM tissue versus intact skin, cultures of primary melanoma cells were established from ghMs of horse DOL (DOLmc; DOLrmc) and TIM (TIMmc), as well as a ghM liver metastasis of horse ELL (ELLmtc). Subsequently, these tumor cells versus normal equine skin fibroblasts (ArcA) were subjected to differential protein expression analysis through quantitative mass spectrometry, followed by a statistical and bioinformatic evaluation of the data.

**Figure 2:**
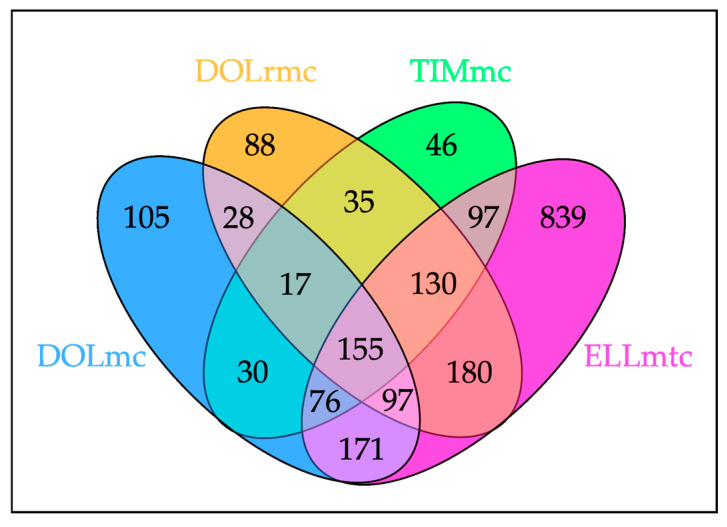
Venn diagram showing the distribution of differentially expressed proteins in gray-horse melanoma (ghM) cell lines compared to normal fibroblasts. The diagram highlights both the shared (*n* = 155) and unique sets of deregulated proteins across the four tumor cell lines. Notably, a high number of proteins were specifically deregulated in the liver-metastasis-derived cells (ELLmtc), indicating a distinct proteomic signature associated with metastatic disease. DOLmc: cells from a primary melanoma affecting horse DOL; DOLrmc: cells from the melanoma of horse DOL that recurred following excision of the primary lesion; TIMmc: melanoma cells from horse TIM; ELLmtc: tumor cells from the liver metastasis of horse ELL.

The comparison of the cell expression profiles included only proteins with a minimum change of twofold in relative intensity and *p* ≤ 0.05. In total, 2909 proteins were identified and quantified, with 155 proteins showing deregulated expression in all four tumor cell lines compared to fibroblasts, as shown in Figure 2. The complete dataset is provided in Supplementary File S3 (“File S3-AllProteomicsData”).

**Figure 3:**
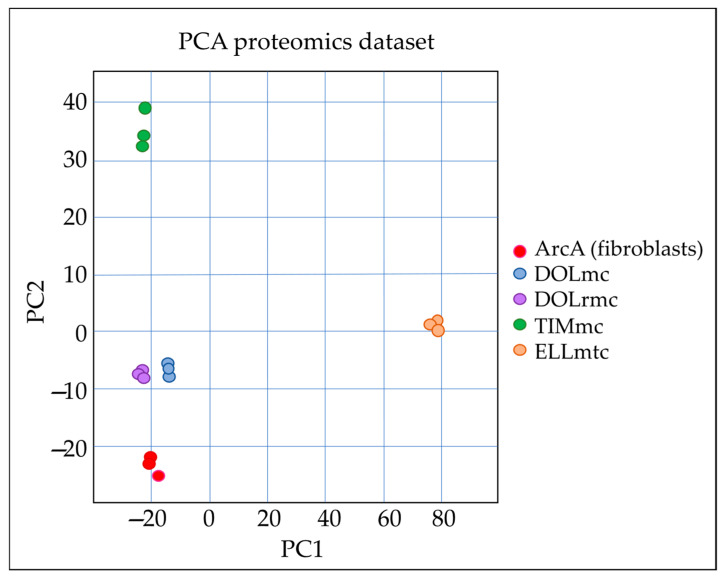
PCA of differential expression data. The biplot reflects the consistency of the data obtained for the three replicates per cell line (note the co-localization of respective dots) and a clear segregation of the different cell cultures with respect to their individual protein expression profiles. This segregation was particularly pronounced in the case of ELLmtc, i.e., ghM cells derived from a liver metastasis. PC1: Principal Component 1; PC2: Principal Component 2.

A principal component analysis (PCA) reflected the good quality of the obtained data, as depicted in Figure 3.

The expression of different melanoma markers varied between cell cultures, as shown in Table 3.

The 155 proteins differentially expressed in all tumor cells compared to normal control fibroblasts were subjected to pathway analysis. A total of 124 identifiers were found in the Reactome, where 1498 pathways were hit by at least one of the proteins. The most significant results, sorted by *p*-value, are presented in Table 4.

Quantitative proteome analysis revealed the enrichment of known pathways in cancer as a common feature of the primary ghM and ghM metastasis cells, irrespective of their origin. These notably included the BRAF/RAS and MAPK signaling networks and pathways associated with stress response (HSF1, NFE2L2) (Table 4).

Given the high number of proteins specifically deregulated in the cells from the ghM liver metastasis (ELLmtc) compared to the other ghM cells (Figure 2 and Figure 3) and normal fibroblasts, we next determined the pathways most significantly overrepresented in the metastasis cells. The identified pathways were exclusively clustered within the fibroblast growth factor receptor (FGFR/FGFR2) signaling network, the insulin receptor/growth factor receptor (IRS/IGF1R) signaling network, or the phosphoinositide 3 kinase/protein kinase B (PI3K/AKT) signaling networks (Table 5).

## 3. Discussion

In horses, melanoma is predominantly diagnosed in gray individuals. This is due to a tight association of the gray genotype with the incidence of the disease [8]. Apart from this important insight, little is known regarding ghM onset and progression, and there are no effective therapies to treat the disease [5]. In addition, the quiescence of ghM in a subset of affected horses over several years has partially led to the interpretation of ghM as a benign disease that requires neither treatment nor particular research efforts [5,6,7,12,27]. Indeed, gray horses with melanoma can reach old age, as previously observed, e.g., in a cohort of 296 horses of the Lipizzaner breed. In this cohort, 148 individuals (50%) were affected by ghM, with 51 of the melanoma-bearing horses being older than 15 years [6]. On the other hand, gray individuals can prematurely succumb to disease [5,6]. Once a ghM is diagnosed, it is impossible to predict the course that the disease will take. There is virtually no information on the pathobiological mechanisms underlying ghM. As a consequence, prognostic markers and therapeutic targets are still lacking.

In order to help reduce this gap of knowledge, ghM tissue versus intact gray-horse skin samples were first subjected to a pilot transcription analysis using RNAseq, followed by bioinformatics. Given the low number of sample specimens included in the experiment, which certainly constituted a limiting factor of the study, stringent evaluation criteria were applied; only transcription levels showing a deregulation of at least twofold with *p* < 0.001 and FDR < 0.001 were regarded as significant. This approach resulted in the identification of 765 genes with upregulated transcription and 1175 genes with downregulated transcription in ghM compared to intact skin.

The obtained data unveiled, inter alia, the deregulation of several genes commonly used as diagnostic hMM markers, such as MITF, TYR, TYRP 1, and gp100. Surprisingly, the melanoma cell adhesion molecule MCAM (CD146) was found to be downregulated in tumors compared to skin. This finding was somewhat surprising, since overexpression of this molecule has been associated with either the onset or the malignant progression of most solid cancers, most notably hMM [28]. Given that MCAM is mainly expressed by tumor cells and endothelial cells [28], the lower MCAM transcription levels in ghM compared to skin may be explained by the poor vascularization of the encapsulated ghMs included in the study compared to the normal-skin controls, which were well vascularized. This explanation is strengthened by the observation that the MCAM protein was found to be overexpressed on the cell level, i.e., in DOLrmc and TIMmc, compared to normal fibroblasts.

Deregulation of gene transcription in ghM also affected GTPase activity and signaling. The small related GTPases of the RAS and the RHO families are known to drive oncogenic processes when activated by growth factors or mutations [29]. The RHO GTPase RAC1, for example, is crucially involved in cytoskeleton regulation and the production of reactive oxygen species (ROSs) [30]. The molecule is also key to vascular homeostasis. In hMM, wild-type and mutated RAC1 act as oncoproteins by promoting tumor cell proliferation, survival, and motility. In addition, RAC1 mutations were shown to be associated with resistance to drugs, including BRAF inhibitors [29,31].

Notably, deregulated gene transcription in ghM also affected pathways involved in the organization and remodeling of the ECM. This deregulation was reflected, e.g., by the enhanced transcription of several matrix metalloproteinases (MMPs), such as MMP1, with protumoral activity in hMM [32,33,34,35] and the reduced transcription of tissue inhibitors of metalloproteinases (TIMPs). Similarly, genes coding for several matricellular proteins such as osteonectin (SPARC), as well as ECM proteoglycans, were over-transcribed in ghM. Overall, the ECM-related transcription pattern observed in the tumor samples pointed to the epithelial–mesenchymal transition (EMT) of tumor cells and ECM degradation events, allowing mesenchymal-type tumor cells to detach from the primary lesion and migrate through the ECM as a hallmark of cancer progression [14,16,17,36].

Following RNAseq analysis, cultured primary tumor cells established from fresh ghM lesions, including a liver metastasis, were subjected to protein expression analysis using quantitative mass spectrometry. Given that equine melanocytes were not available, gray-horse primary fibroblasts were used as control cells in this experiment. Following proteomics analysis and stringent evaluation of data, the 155 proteins with deregulated expression in ghM cells were subjected to pathway analysis. Similar to what was observed on the transcription level, GTPase-related pathways—notably, those involving RAC1, RAC3, and RAS proteins—were significantly enriched in the tumor cells.

Under physiological conditions, the RAS/RAF/MAPK signaling cascade regulates cell proliferation, differentiation, and survival. New technologies such as next-generation sequencing (NGS) have unveiled that this cascade is usually altered by somatic mutations in hMM [37]. HMMs that are not directly attributable to cumulative sun damage typically harbor BRAF V600E mutations, whilst UV-induced lesions commonly present with other MAPK-pathway mutations, including BRAF V600K, NRAS G12/G13, and KIT mutations, or with suppressed negative regulators of RAS [38]. Irrespective of their origin, these mutations have a crucial role in hMM development, as they promote tumor cell hyperproliferation and survival via constitutive activation of the serine–threonine kinases in the ERK–MAPK pathway [39]. In ghM, Reactome analysis pointed to the enrichment of signaling pathways by and downstream of RAS mutants. Similarly, an enrichment of RAF/BRAF and oncogenic MAPK signaling was observed in ghM cells. On these grounds, screening of ghM for gene mutations affecting the RAS/RAF/MAPK signaling network will be carried out in the near future.

The high number of proteins with deregulated expression in ghM metastasis cells led to the idea to next determine the pathways that were overrepresented in these cells. Reactome analysis resulted in the identification of three groups of enriched pathways. The first comprised pathways related to fibroblast growth factor receptor 2 (FGFR 2) signaling. In mammals, signaling by FGFR2 and its key-ligand fibroblast growth factor 10 (FGF10) assures normal epidermal growth and development, as well as hair follicle patterning [40]. In skin cancers, the role of FGFR2 is not completely understood. For example, there is evidence that loss of the tumor-suppressing lipid phosphatase PTEN and, thereby, induced autocrine FGF10/FGFR2 signaling may support the development of cutaneous squamous cell carcinoma (SCC) [41]. On the other hand, there are indications that FGFR2 can act as a tumor suppressor in the skin [42]. In addition, polyclonal point mutations in the kinase domain of FGFR2 were shown to induce drug resistance in human cholangiocarcinoma (CCA) patients [43]. Missense mutations leading to the loss of function of FGFR2 are also reported in hMM, but their biological significance is still unclear [44]. In ghM, the role and mutation status of FGFR2 still remain to be elucidated. Provided that FGFR2 would indeed have a tumor-suppressive function, loss-of-function mutations of FGFR2 in ghM may help promote metastasis.

The second cluster of pathways enriched in ghM metastasis cells comprised the insulin receptor (IRS) and insulin-like growth factor 1 receptor (IGF1R) signaling cascades. It is accepted today that the IGF1-IGF1R system is tightly associated with the sustained proliferation, migration, and survival of cancer cells, as well as with tumor neovascularization [45]. In addition, IGF1 upregulates the expression of the anti-apoptotic proteins Bcl-2, Bcl-XL, and survivin, thus promoting a drug-resistant tumor cell phenotype associated with metastatic hMM [46]. The overrepresentation of IRS/IGF1R pathways in the ghM metastasis cells agrees with these data.

The third group of enriched pathways encompassed the phosphoinositide 3 kinase/protein kinase B (PI3K/AKT) signaling network. Activation of PI3K/AKT signaling is one of the most frequently detected events in human cancers, including hMM, where it is mediated, inter alia, by the above-mentioned mutations, e.g., in RAS family members [47]. In addition, PI3K/AKT signaling in hMM is activated by the loss of function of PTEN [47], which is similar to what is observed for autocrine FGF10/FGFR2 signaling. The enrichment of the PI3K/AKT signaling network in ghM metastasis cells agrees with the malignant behavior of the disease in horse ELL. Given the crucial role of PTEN in skin homeostasis and the implications of its loss of function in hMM, in-depth studies on the mutational state and functionality of PTEN in ghM are warranted.

## 4. Materials and Methods

### 4.1. Sample Material

Tumor tissue was obtained from eight gray horses with ghM. Intact skin was collected from two ghM-affected and two tumor-free gray individuals. All horse and sample specifications are provided in Table 6.

Tumor specimens were obtained with the owners’ written consent and in accordance with the guidelines of the Veterinary University Vienna, Austria, authorizing the scientific use of tissue excised for therapeutic or diagnostic reasons. Intact skin was collected from the necks of horses DIM, GYN, and HAC (Table 6) with the approval of the institutional ethics committee and the Federal Ministry of Science and Research (BMWF; license Nr.: GZ 68.205/0102-II/3b/2013) under local anesthesia using a 4 mm biopsy punch. One skin sample was excised from the axilla of the ghM-free horse ARC immediately after euthanasia with the owner’s written consent.

The lesions were diagnosed as ghM through clinical examination and histopathological assessment of tumor sections, as described previously [48]. In one case (ELL), necropsy was also carried out.

### 4.2. Transcriptome Analysis in Gray-Horse Melanoma Versus Intact Skin

RNAseq analysis was used to assess differential gene transcription in ghM versus intact skin, exactly as previously described [12,48]. To purify RNA, tumor and intact skin aliquots kept in 500 µL of TRI Reagent^®^ (Sigma-Aldrich, Vienna, Austria) were homogenized, incubated with chloroform, and then centrifuged. The upper aqueous phases were transferred to 1.5 mL Eppendorf tubes and supplemented with ice-cold isopropanol for RNA precipitation. Following centrifugation, the obtained RNA pellets were washed with 75% ethanol, air-dried, and resuspended in 20 µL of sterile water. Subsequent DNase digestion was carried out in RNeasy columns (Qiagen, Hilden, Germany) according to the instructions of the manufacturer. The obtained DNA-free RNA was eluted with 22 µL of sterile water into 1.5 mL Eppendorf tubes, each containing 1 µL of the RNase inhibitor (RNaseOUT; Invitrogen, Thermo Fisher Scientific, Vienna, Austria). Melanin remnants, as occasionally detected according to the yellowish color of eluates, were removed with further RNA purification using the RNeasy Mini kit (Qiagen) according to the instructions of the manufacturer.

RNA yields were determined via photometry (Eppendorf BioPhotometer^TM^ D30, Hamburg, Germany). RNA integrity numbers (RINs) were assessed using a Bioanalyzer RNA 6000 Nano assay (Agilent Technologies, Vienna, Austria). RNA isolates with an OD 260/280 ranging between 1.8 and 2.2, an OD 260/230 of >2.0, and an RIN of >7 were subjected to RNAseq and basic bioinformatics analyses (BGI Genomics, Shenzhen, China).

The obtained raw data were processed further. Following the removal of adaptors and low-quality reads, the remaining reads were aligned to the whole-genome Shotgun assembly EquCab2 (GenBank Assembly ID GCA_000002305.1) of the thoroughbred mare “Twilight” [49] using the SOAP2 alignment program [50]. Uniquely mapped reads in proper pairs were subsequently gene-mapped. To identify differentially transcribed genes, the levels of reads per kilobase per million reads (RPKM) were calculated. The false discovery rate method (FDR) was used to correct for multiple tests [51]. Genes revealing a ≥2-fold deregulation of transcription in ghM, an FDR of <0.001, and a *p*-value of <0.001 were considered as being differentially transcribed in the tumors compared to normal skin and subjected to pathway analysis using the open-source, peer-reviewed pathway database https://reactome.org/ [52].

### 4.3. Proteome Analysis in ghM Cells Versus Fibroblasts

The proteome analysis was carried out from primary ghM cells and gray-horse fibroblasts that we previously established [12,53].

According to their respective sources (Table 1), the tumor cells received the designations DOLmc (eqRGO1), DOLrmc (eqRGO1_2R), TIMmc (eqRGO4), and ELLmtc (eqRGO6), with “mc” denoting melanoma cells, “rmc” denoting cells from a recurrent melanoma, and “mtc” denoting cells from a metastasis. Gray-horse fibroblasts (ArcA) originated from intact axillary skin of a ghM-free individual ARC [53].

For protein isolation, the cultured tumor cells and fibroblasts (10^6^ cells/sample) were harvested and resuspended in 100 μL of urea-based denaturing buffer (6 M urea, 2 M thiourea, and 10 mM HEPES; pH 8.0; all reagents from Sigma-Aldrich). Protein disulfide bonds were reduced via incubation with dithiothreitol (DTT; 10 mM; Sigma-Aldrich) for 30 min at room temperature. Then, the samples were alkylated by incubation with iodoacetamide (55 mM) solution (Sigma-Aldrich) in the dark at room temperature. After 30 min, the samples were diluted with ammonium bicarbonate buffer (40 mM) in order to reduce the urea concentration below 2 M. Protein digestion was conducted with trypsin protease (1 µg/sample; ThermoFisher Scientific, Vienna, Austria) at room temperature overnight. Then, all samples were acidified through the addition of 5% acetonitrile (ThermoFisher Scientific) and 0.3% trifluoroacetic acid (TFA; Sigma-Aldrich), and they were desalted using C18 StageTips with Empore™ C18 Extraction Disks (ThermoFisher Scientific) [54]. After elution of the peptides from the StageTips, they were dried through vacuum centrifugation.

Dried peptides were reconstituted in 40 µL of 0.05% trifluoroacetic acid and 4% acetonitrile in water. Subsequently, 1 µL of each sample was analyzed using an Ultimate 3000 reversed-phase capillary nano liquid chromatography system connected to a Q Exactive HF mass spectrometer (Thermo Fisher Scientific). Samples were injected and concentrated on a trap column (PepMap100 C18, 3 µm, 100 Å, 75 µm i.d. × 2 cm, Thermo Fisher Scientific) equilibrated with 0.05% TFA in water. After switching the trap column inline, LC separations were performed on a capillary column (double nanoViper PepMap Neo C18, 2 µm, 100 Å, 75 µm i.d. × 50 cm, Thermo Fisher Scientific) at an eluent flow rate of 300 nl/min. Mobile phase A contained 0.1% formic acid in water, and mobile phase B contained 0.1% formic acid in 80% acetonitrile/20% water. The column was pre-equilibrated with 5% mobile phase B followed by an increase of 5–44% in mobile phase B in 70 min. Mass spectra were acquired in a data-dependent mode utilizing a single MS survey scan (*m*/*z* 300–1650) with a resolution of 60,000 and MS/MS scans of the 15 most intense precursor ions with a resolution of 15,000 with a normalized collision energy of 27. The dynamic exclusion time was set to 20 s, and the automatic gain control was set to 3 × 10^6^ and 1 × 10^5^ for MS and MS/MS scans, respectively.

The analysis of MS and MS/MS raw data was conducted using the MaxQuant software package e (version 2.0.3.0) with the Andromeda peptide search engine [55]. The Equus caballus reference proteome (ID: UP000002281; downloaded from https://www.uniprot.org/ on 6 November 2022; 44,484 sequences) was searched against the data using the default parameters, along with the options of label-free quantification (LFQ) and a match between runs. Data filtering and statistical analysis were performed using the Perseus 1.6.14 software [56].

For downstream analysis, only proteins that were identified and quantified with LFQ intensity values in at least three replicates within one of the five experimental groups were considered. Missing values were imputed from a normal distribution using the default settings (width 0.3, downshift 1.8). Mean log2-fold differences between groups were calculated using Student’s *t*-test. Proteins with a minimum 2-fold intensity change compared to the control (log2-fold change ≥ 1 or log2-fold change ≤ −1) and a *p*-value of ≤0.05 were considered significantly altered in their expression and subjected to pathway involvement analysis using https://reactome.org/ [52].

## 5. Conclusions

To the authors’ knowledge, this is the first report presenting the transcription and protein expression signatures of ghM and the primary tumor cells derived therefrom.

The obtained data underline that typical markers used for hMM diagnosis are not consistently expressed in ghM and by the primary cells derived therefrom. Hence, a combination of markers, e.g., TYRP, MAGE D2, and MCAM, should be used for immunohistochemical or immunofluorescence-based diagnosis of ghM.

Overall, ghM lesions and cells exhibited gene transcription and protein expression patterns that selectively overlapped. However, it has to be stressed that RNAseq and proteomics data were not statistically compared on the grounds that they described different types of samples, that is, whole tumor and skin tissue on one side, with each harboring a plethora of different cell types, and monocultures of tumor cells and fibroblasts on the other.

However, the ghM gene transcription and protein expression signatures closely resembled those typically observed in human cancers, notably hMM. Despite the low number of tumor tissue samples and cancer cells analyzed, the presented data clearly show that ghM is a disease with high malignant potential. Hence, substantially more efforts should be directed towards a better pathobiological understanding of ghM and the establishment of more effective therapies.

The data reported herein indicate that ghM and hMM are—at least in part—driven by similar pathways, e.g., RAS/RAF/MAPK, or PI3K/AKT. On these grounds, corresponding inhibitors used in human medicine may also prove effective in the treatment of ghM. This possibility should be further exploited.

The obtained data also suggest that ghM may harbor activating mutations in RAC1, RAS, BRAF, or PTEN, similar to what is observed in hMM. Given the impact of these mutations on disease onset and progression in humans, determining the mutational status of ghM has the potential to significantly help advance the understanding and management of this frequent disease in gray horses.

## Figures and Tables

**Table 1:** Deregulated transcription of melanoma markers in ghM.

Transcription	Genes	Definition
Upregulated	*MAGE D2*	Melanoma-associated antigen D2
	*MITF*	Microphthalmia-associated transcription factor
	*PMEL17 (SILV, gp100)*	Premelanosome protein
	*TYR*	Tyrosinase
	*TYRP1*	Tyrosinase related protein 1
Downregulated	*MCAM (CD146; MUC18)*	Melanoma cell adhesion molecule
	*S100A6*	S100 Calcium Binding Protein A6; Calcyclin
	*S100A7*	S100 Calcium Binding Protein A7; Psoriasin

**Table 2:** Reactome pathways that are most significantly overrepresented in ghM (*p* ≤ 0.001).

Pathway Name	Reactome Entities				Reactions	
	Found	Ratio	*p*-Value	FDR	Found	Ratio
RAC1 GTPase cycle	54/191	0.012	4.76 × 10^−6^	0.006	4/6	3.93 × 10^−4^
ECM organization	84/350	0.0022	8.06 × 10^−6^	0.006	256/330	0.022
RHO GTPase cycle	104/460	0.029	8.76 × 10^−6^	0.006	52/91	0.006
RHOA GTPase cycle	44/154	0.01	2.70 × 10^−5^	0.014	4/6	3.93 × 10^−4^
Integrin cell surface interaction	29/86	0.005	3.87 × 10^−5^	0.016	44/55	0.004
ECM proteoglycans	27/79	0.005	5.63 × 10^−5^	0.019	20/23	0.002
Activation of gene expression by SREBF	24/71	0.005	1.65 × 10^−4^	0.049	42/42	0.003
GLI proteins bind promoters of Hh-responsive genes to promote transcription	7/8	5.07 × 10^−4^	1.99 × 10^−4^	0.052	4/4	2.62 × 10^−4^
Non-Integrin membrane-ECM interactions	26/83	0.005	2.89 × 10^−4^	0.058	19/33	0.002
AP−2 family regulates transcription of growth factors and their receptors	11/21	0.001	3.15 × 10^−4^	0.058	9/18	0.001
TRP channels	14/32	0.002	3.20 × 10^−4^	0.058	1/7	4.59 × 10^−4^
EGR2- and SOX10-mediated initiation of Schwann cell myelination	16/40	0.003	3.32 × 10^−4^	0.058	26/27	0.002
Formation of the cornified envelope	37/138	0.009	3.71 × 10^−4^	0.059	17/27	0.002
Syndecan interactions	13/29	0.002	4.12 × 10^−4^	0.061	9/15	9.83 × 10^−4^
Kinesins	22/68	0.004	5.36 × 10^−4^	0.074	11/14	9.17 × 10^−4^
Degradation of the ECM	38/148	0.009	6.88 × 10^−4^	0.085	80/105	0.007
RND3 GTPase cycle	16/43	0.003	7.12 × 10^−4^	0.085	1/2	1.31 × 10^−4^
Defective B3GALTL causes PpS	15/39	0.002	7.43 × 10^−4^	0.085	1/1	6.55 × 10^−5^
Assembly of collagen fibrils and other multimeric structures	21/67	0.004	0.001	0.114	23/26	0.002
O-glycosylation of TSR domain-containing proteins	15/41	0.003	0.001	0.122	2/2	1.31 × 10^−4^
Type I hemidesmosome assembly	7/11	6.98 × 10^−4^	0.001	0.122	6/6	3.93 × 10^−4^
CDC42 GTPase cycle	39/159	0.01	0.001	0.122	4/6	3.93 × 10^−4^
EPH-ephrin-mediated repulsion of cells	18/55	0.003	0.001	0.122	9/9	5.90 × 10^−4^
ERBB2 activates PTK6 signaling	9/18	0.001	0.001	0.122	2/2	1.31 × 10^−4^
Signaling by RHO GTPases	133/708	0.045	0.002	0.127	113/203	0.013

**Table 3:** Upregulated melanoma markers in ghM cells compared to fibroblasts.

DOLmc	DOLrmc	TIMmc	ELLmtc
	MCAM	MCAM	
	MAGE D2		
			MLANA
			PMEL
S100A1			S100A1
		S100A10	
S100A11	S100A11		
S100A4	S100A4		S100A4
S100A6	S100A6	S100A6	S100A6
			TYR
			TYRP

**Table 4:** The most significantly enriched pathways in ghM cells (*p* ≤ 0.009).

Pathway Name	Entities				Reactions	
	Found	Ratio	*p*-Value	FDR	Found	Ratio
HSF1-dependent transactivation	8/59	0.002	3.93 × 10^−7^	6.42 × 10^−4^	5/8	5.24 × 10^−4^
Attenuation phase	7/47	0.002	1.14 × 10^−6^	9.34 × 10^−4^	3/5	3.28 × 10^−4^
NFE2L2 regulates pentose phosphate pathway genes	4/23	9.52 × 10^−4^	1.35 × 10^−4^	0.074	4/8	5.24 × 10^−4^
Orc1 removal from chromatin	5/64	0.003	7.74 × 10^−4^	0.211	3/4	2.62 × 10^−4^
Signaling by BRAF and RAF1 fusions	5/73	0.003	0.001	0.322	5/5	3.28 × 10^−4^
RHOF GTPase cycle	4/46	0.002	0.002	0.363	1/3	1.97 × 10^−4^
Regulation of HSF1-mediated heat shock response	13/302	0.013	0.002	0.378	11/14	9.17 × 10^−4^
NFE2L2 regulating TCA cycle genes	2/7	2.90 × 10^−4^	0.003	0.455	2/4	2.62 × 10^−4^
RHOD GTPase cycle	4/57	0.002	0.004	0.566	1/9	5.90 × 10^−4^
Cellular response to heat stress	14/391	0.016	0.004	0.591	17/29	0.002
RAC3 GTPase cycle	5/100	0.004	0.005	0.591	1/6	3.93 × 10^−4^
DNA strand elongation	5/101	0.004	0.005	0.591	7/15	9.83 × 10^−4^
RSK activation	2/11	4.55 × 10^−4^	0.007	0.665	4/4	2.62 × 10^−4^
Oncogenic MAPK signaling	5/112	0.005	0.008	0.665	30/46	0.003
Signaling by moderate kinase activity BRAF mutants	4/73	0.003	0.009	0.665	5/7	4.59 × 10^−4^
Signaling downstream of RAS mutants	4/73	0.003	0.009	0.665	5/7	4.59 × 10^−4^
Paradoxical activation of RAF signaling by kinase inactive BRAF	4/73	0.003	0.009	0.665	5/7	4.59 × 10^−4^
Activation of ATR in response to replication stress	5/73	0.003	0.009	0.665	5/9	5.90 × 10^−4^
Signaling by RAS mutants	4/73	0.003	0.009	0.665	5/9	5.90 × 10^−4^
CREB1 phosphorylation through NMDA receptor-mediated activation of RAS signaling	3/39	0.002	0.009	0.669	7/7	4.59 × 10^−4^

**Table 5:** Clusters of pathways that were significantly overrepresented in gray-horse melanoma metastasis (ELLmtc).

Cluster 1	Cluster 2	Cluster 3
Signaling by FGFR Signaling by FGFR2 SHC-mediated cascade: FGFR2 FRS-mediated FGFR2 signaling Signaling by FGFR2 IIIa TM Downstream signaling of FGFR2 FGFR2 mutant receptor activation	Insulin receptor signaling cascade IRS-mediated signaling IRS-related events triggered by IGF1R IGF1R signaling cascade Signaling by IGF1R AP-2 family regulates transcription of growth factors and their receptors	PI5P, PP2A and IER3 regulate PI3K/AKT signaling Negative regulation of PI3K/AKT network Constitutive signaling by aberrant PI3K in cancer

**Table 6:** Horse and sample specifications.

Code	Breed	Sex	Age	Short Disease Description	Samples Collected
ARI	Warmblood	G	22	Four melanomas, thoracic region	One tumor
DIM	Trakehner mix	G	14	Multiple melanomas in the anogenital, parotidal, ocular, and labial regions	One anogenital tumor^,^
					Intact skin
DOL	Icelandic horse	G	14	Multiple anogenital melanomas and a recurrent lesion after surgical excision	One anogenital tumor
					Recurrent tumor
ELL	Trakehner mix	M	13	Large perianal tumor, metastases (spleen, liver, heart)	Liver metastasis
GYN	Shagya Arabian	M	>20	Two encapsulated melanomas under the tail root, maximum diameter < 2 cm	One tumor
					Intact skin
MAN	Lipizzan horse	M	14	Two encapsulated melanomas under the tail root	One tumor
MAK	Arabian horse	S	4	Single small, encapsulated melanoma, inner thigh	Tumor
TIM	Warmblood	M	15	Single encapsulated melanoma under the tail root	Tumor
HAC	PRE	S	13	Melanoma-free	Intact skin
ARC	GRP	G	19	Melanoma-free	Intact skin

PRE: Pura Raza Espagnola; GRP: German Riding pony; G: gelding, M: mare; S: stallion; age in years.

## Data Availability

Major research data are presented in the main article and Supplementary File S1. Raw data will be made available by the corresponding author upon request.

## Supplementary Materials

The following supporting information can be downloaded at: https://www.mdpi.com/article/10.3390/ijms26146620/s1.

## Abbreviations

The following abbreviations are used in this manuscript:

MM	malignant melanoma
hMM	human malignant melanoma
ghM	gray-horse melanoma
VM	vasculogenic mimicry
RNAseq	RNA sequencing
FDR	false discovery rate
RPKM	reads per kilobase million
