# Supplementary material for: Gray-Horse Melanoma—A Wolf in Sheep’s Clothing
Source: Int J Mol Sci. 2025 Jul 10;26(14):6620. doi: 10.3390/ijms26146620 (PMC12295847; doi:10.3390/ijms26146620)
Supplement: Supplementary file 1 [file ijms-26-06620-s001.zip › File S1-RNAseq-Upregulated.pdf]

Table S1: Genes with upregulated transcription in ghM compared to intact skin

Gene ID	Gene	Transcription		ghM sum/ skin sum	Regulation	P value	FDR
		skin sum	ghM sum				
ABAT	4-aminobutyrate aminotransferase	54	264	4.89	Up	6.84E-33	1.89E-23
ABCA6	ATP binding cassette subfamily A member 6	693	1359	1.96	Up	1.62E-54	5.56E-47
ABCA9	ATP binding cassette subfamily A member 9	3826	20300	5.31	Up	0	0
ABCC8	ATP binding cassette subfamily C member 8	113	377	3.34	Up	1.34E-32	3.73E-24
ABCG2	ATP binding cassette subfamily C member 2	758	2110	2.78	Up	1.26E-162	7.71E-153
ABI3BP	ABI family member 3 binding protein	5655	33322	5.89	Up	0	0
ACP2	Acid phosphatase 2, lysosomal	1948	7762	3.98	Up	0	0
ACSS1	Acyl-coa synthetase short chain family member 1	560	1782	3.18	Up	5.48E-162	3.35E-152
ACTA1	Actin alpha 1 skeletal muscle	94	5221	55.54	Up	0	0
ACTN2	Actinin alpha 2	18	176	9.78	Up	1.70E-30	4.61E-22
ACTN3	Actinin alpha 3	130	2792	21.48	Up	0	0
ADAD2	Adenosine deaminase domain containing 2	10	354	35.40	Up	1.22E-91	5.37E-83
ADAM28	A disintegrin and metalloprotease 28	83	435	5.24	Up	2.40E-60	8.46E-50
ADAM9	A disintegrin and metalloprotease 9	2300	9504	4.13	Up	0	0
ADAMTS15	ADAM with thrombospondin motifs	290	714	2.46	Up	8.29E-44	2.57E-34
ADCY2	Adenylate cyclase 2	367	2623	7.15	Up	0	0
ADRBK2	G-protein-coupled receptor kinase 3	629	3472	5.52	Up	0	0
AFF3	ALF transcription elongation factor 3	177	474	2.68	Up	4.07E-33	1.12E-22
AGM	Abnormal gametophytes	8659	19130	2.21	Up	0	0
AGTR2	Angiotensin II receptor type 2	8	2520	315.00	Up	0	0
AHI1	Abelson helper integration site 1	1856	4987	2.69	Up	0	0
AIFM1	Apoptosis inducing factor mitochondria associated 1	3281	8896	2.71	Up	0	0
AIP	Aryl hydrocarbon receptor interacting protein	1957	4877	2.49	Up	0	0
AK8	Adenylate kinase 8	47	240	5.11	Up	1.28E-29	3.47E-21
AKAP11	A-kinase anchor protein 11	1385	2665	1.92	Up	4.03E-107	1.92E-97
AKAP12	A-kinase anchor protein 12	3475	11591	3.34	Up	0	0
AKAP6	A-kinase anchor protein 6	193	703	3.64	Up	5.44E-72	2.12E-63
AKR1A1	Aldo-keto reductase family 1 member A1	6828	16619	2.43	Up	0	0
AKT3	AKT Serine/Threonine kinase 3	1135	3425	3.02	Up	6.61E-295	6.08E-285
ALB	Albumin	266	953	3.58	Up	4.49E-97	2.02E-87
ALDH1A2	Aldehyde dehydrogenase 1 A2	20	2232	111.60	Up	0	0
ALDH3B1	Aldehyde dehydrogenase 3 B1	3863	17490	4.53	Up	0	0
ALDH9A1	Aldehyde dehydrogenase 9 A1	1269	5436	4.28	Up	0	0
ALG6	Alg6 alpha-1,3-glucosyltransferase	616	1330	2.16	Up	1.09E-65	4.08E-57
ALOX5	Arachidonate 5-lipoxygenase	434	3802	8.76	Up	0	0
ALX1	ALX Homeobox 1. Overexpressed in HMM	41	2131	51.98	Up	0	0
AMIGO2	Adhesion Molecule with Ig Like Domain 2	96	391	4.07	Up	4.04E-43	1.25E-33
AMPD1	Adenosine monophosphate deaminase 1	0	75	-	Up	1.75E-18	4.10E-11
ANKRD32	Ankyrin repeat domain-containing protein 32	431	859	1.99	Up	3.21E-35	9.11E-26
ANKRD34A	Ankyrin repeat domain-containing protein 34A	47	228	4.85	Up	9.20E-29	2.39E-19
ANKRD34B	Ankyrin repeat domain-containing protein 34B	92	5785	62.88	Up	0	0
ANKRD5	Ankyrin repeat domain-containing protein 5	53	469	8.85	Up	4.76E-86	2.02E-76
ANKRD52	Ankyrin repeat domain-containing protein 52	6691	22914	3.42	Up	0	0

ANKS1B	Ankyrin Repeat and Sterile Alpha Motif Domain Containing 1B	87	6935	79.71	Up	0	0
ANO4	Anoctamin 4	7	590	84.29	Up	8.81E-169	5.54E-159
ANTXR1	Anthrax toxin receptor 1	3860	15174	3.93	Up	0	0
ANXA6	Annexin A6	8634	26366	3.05	Up	0	0
AOAH	Acyloxyacyl hydrolase	618	4915	7.95	Up	0	0
AP2M1	Adaptor related protein complex 2 subunit Mu 1	27895	55699	2.00	Up	0	0
AP3M2	Adaptor related protein complex 3 subunit mu 2	176	2942	16.72	Up	0	0
APBB1	Amyloid beta precursor protein binding family B member 1	1168	2275	1.95	Up	1.34E-92	5.93E-84
APH1B	Aph-1 homolog B, Gamma-secretase subunit	200	416	2.08	Up	4.84E-17	1.09E-07
APOBEC3Z3	Apolipoprotein B mrna editing enzyme, catalytic polypeptide-like 3Z3	97	384	3.96	Up	3.68E-42	1.11E-31
ARAP1	Arfgap with rhogap domain, Ankyrin Repeat and PH Domain 1	4243	8901	2.10	Up	0	0
ARHGAP1	Rho gtpase Activating Protein 1	7016	13253	1.89	Up	0	0
ARHGAP15	Rho gtpase Activating Protein 15	403	1170	2.90	Up	5.58E-94	2.48E-84
ARHGAP20	Rho gtpase Activating Protein 20	62	299	4.82	Up	2.68E-37	7.77E-28
ARHGAP24	Rho gtpase Activating Protein 24	1445	2710	1.88	Up	1.62E-101	7.60E-93
ARHGAP31	Rho gtpase Activating Protein 31	1279	3957	3.09	Up	0	0
ARHGAP9	Rho gtpase Activating Protein 9	370	790	2.14	Up	2.00E-37	5.81E-28
ARHGEF6	Rac/Cdc42 Guanine nucleotide exchange factor 6	446	839	1.88	Up	9.43E-30	2.52E-20
ARNT2	Aryl hydrocarbon receptor nuclear translocator 2	242	577	2.38	Up	7.28E-33	2.01E-23
ARPC1B	Actin related protein 2/3 complex subunit 1B	12738	25289	1.99	Up	0	0
ARVCF	Armadillo Repeat gene deleted in Velo-Cardio-Facial syndrome	767	5795	7.56	Up	0	0
ASB2	Ankyrin repeat and SOCS box-containing protein 2	90	463	5.14	Up	1.54E-61	5.54E-53
ASL	Argininosuccinate lyase	1694	6175	3.65	Up	0	0
ASPA	Aspartoacylase	246	1287	5.23	Up	1.17E-181	7.70E-173
ATG7	Autophagy related 7	1149	2450	2.13	Up	1.92E-121	9.90E-113
ATP2B1	Atpase plasma membrane ca2+ transporting 1	7621	20326	2.67	Up	0	0
ATP6AP1	Atpase h+ transporting accessory protein 1	12086	40484	3.35	Up	0	0
ATP6V0D2	Atpase h+ transporting v0 subunit d2	381	13026	34.19	Up	0	0
ATP8A1	Atpase phospholipid transporting 8A1	629	4734	7.53	Up	0	0
ATP8B4	Atpase phospholipid transporting 8B4	114	463	4.06	Up	1.55E-50	5.14E-42
AZIN1	Antizyme inhibitor 1	2375	5533	2.33	Up	0	0
B2M	Beta-2-microglobulin; beta-chain of MHC I	32402	163099	5.03	Up	0	0
BANK1	B Cell Scaffold Protein with Ankyrin Repeats 1	479	1474	3.08	Up	2.99E-128	1.59E-118
BBS7	Bardet-biedl syndrome 7	879	1894	2.15	Up	6.45E-97	2.90E-86
BBS9	Bardet-biedl syndrome 9	532	1439	2.70	Up	1.31E-104	6.19E-96
BBX	BBX High Mobility Group Box Domain Containing protein	2407	4743	1.97	Up	1.26E-202	9.10E-194
BCHE	Butyrylcholinesterase	162	848	5.23	Up	1.13E-118	5.73E-109
BEST1	Bestrophin 1	68	1556	22.88	Up	0	0
BIN2	Bridging integrator 2	1051	2125	2.02	Up	8.68E-95	3.87E-85
BSG	Basigin; CD147; plasma membrane protein	36184	68655	1.90	Up	0	0
BTK	Bruton tyrosine kinase	589	2859	4.85	Up	0	0
C15H2orf40	Equus caballus chromosome 15 open reading frame, transcript X1	432	10383	24.03	Up	0	0
C2	Complement component 2 protein	564	1875	3.32	Up	3.03E-179	1.98E-169
C4A	Complement component 4A protein	630	1342	2.13	Up	1.58E-64	5.84E-56
C7	Complement component 7 protein	694	1314	1.89	Up	3.07E-49	1.00E-39

C8B	Complement component 8B protein	5	111	22.20	Up	4.57E-24	1.14E-14
CAB39L	Calcium binding protein 39 like	1725	4265	2.47	Up	4.13E-279	3.67E-269
CACNA1D	Calcium voltage-gated channel subunit alpha1 D	157	802	5.11	Up	3.74E-110	1.81E-100
CADM1	Cell adhesion molecule 1	3879	11160	2.88	Up	0	0
CADM3	Cell adhesion molecule 3	576	5169	8.97	Up	0	0
CALML4	Calmodulin like 4	214	522	2.44	Up	1.23E-29	3.31E-21
CAMK1G	Calcium/Calmodulin dependent protein kinase IG	55	835	15.18	Up	2.32E-187	1.57E-177
CAMTA1	Calmodulin binding transcription activator 1	631	1430	2.27	Up	7.28E-80	2.96E-73
CAND2	Cullin associated and neddylation dissociated 2	184	563	3.06	Up	1.13E-45	3.60E-37
CAP2	Cyclase associated Actin Cytoskeleton Regulatory Protein 2	110	745	6.77	Up	8.88E-123	4.57E-112
CAPG	Capping Actin Protein, Gelsolin-like	19791	37966	1.92	Up	0	0
CAPN11	Calpain 11	67	468	6.99	Up	1.44E-75	5.79E-67
CASKIN1	Cask interacting protein 1	75	322	4.29	Up	1.17E-35	3.38E-27
CAV2	Caveolin 2	5586	19005	3.40	Up	0	0
CCDC112	Coiled-coil domain-containing protein 112	1196	6005	5.02	Up	0	0
CCDC136	Coiled-coil domain-containing protein 136	167	359	2.15	Up	2.10E-16	4.57E-06
CCDC148	Coiled-coil domain-containing protein 148	19	303	15.95	Up	1.04E-66	3.89E-57
CCDC158	Coiled-coil domain-containing protein 158	267	573	2.15	Up	1.60E-26	4.10E-17
CCDC82	Coiled-coil domain-containing protein 82	836	2611	3.12	Up	1.37E-232	1.07E-223
CCDC88A	Coiled-coil domain-containing protein 88A	3945	9360	2.37	Up	0	0
CCL3	C-C motif chemokine ligand 3	24	201	8.38	Up	5.02E-34	1.41E-24
CD180	Cluster of differentiation 180	29	377	13.00	Up	4.70E-79	1.91E-69
CD40	Cluster of differentiation 40	533	1066	2.00	Up	7.49E-45	2.35E-35
CD68	Cluster of differentiation 68; macrophage marker	3154	123520	39.16	Up	0	0
CDH19	Cadherin 19; high in proliferative HMM	89	821	9.22	Up	8.68E-156	5.17E-146
CDHR2	Cadherin related family member 2	146	3297	22.58	Up	0	0
CDK15	Cyclin-dependent kinase 15	9	876	97.33	Up	1.18E-254	9.77E-246
CDK8	Cyclin-dependent kinase 8	1389	3547	2.55	Up	1.77E-242	1.43E-233
CDKL2	Cyclin-dependent kinase-like 2	42	220	5.24	Up	2.52E-28	6.61E-19
CELF2	Cugbp elav-like family member 2	393	1286	3.27	Up	7.82E-121	3.99E-111
CENPI	Centromere protein i	361	1849	5.12	Up	1.72E-257	1.44E-248
CEP128	C-terminally encoded peptide 128	620	1609	2.60	Up	1.69E-110	8.26E-102
CEP170	C-terminally encoded peptide 170	1104	2327	2.11	Up	1.69E-112	8.33E-104
CFI	Complement factor I	868	2419	2.79	Up	3.55E-187	2.40E-181
CFP	Complement factor properdin	1334	2743	2.06	Up	1.04E-126	5.54E-118
CG	Comb gap protein	93	270	2.90	Up	3.96E-20	9.22E-10
CHD6	Chromodomain helicase dna binding protein 6	3446	6670	1.94	Up	2.84E-277	2.51E-266
CHIT1	Chitinase 1	813	1737	2.14	Up	2.98E-87	1.27E-76
CHST10	Carbohydrate sulfotransferase 10	1688	5631	3.34	Up	0	0
CLASP2	Cytoplasmic linker associated protein 2	5605	11445	2.04	Up	0	0
CLCN4	Chloride channel 4	296	4317	14.58	Up	0	0
CLEC12A	C-type lectin domain family 12 member a	447	1059	2.37	Up	2.36E-62	8.50E-53
CLIP3	CAP-GLY domain containing linker protein 3	1937	5203	2.69	Up	0	0
CLTA	Clathrin light chain A	3181	6129	1.93	Up	5.27E-251	4.34E-241
CLU	Clusterin	30319	58106	1.92	Up	0	0
CNKSR2	Connector enhancer of kinase suppressor of Ras 2	156	1612	10.33	Up	0	0
CNNM1	Cyclin and cbs domain divalent metal cation transport mediator 1	238	956	4.02	Up	9.12E-109	4.38E-99

CNTLN	Centlein	798	1884	2.36	Up	1.05E-111	5.14E-103
CNTN6	Contactin 6	5	1915	383.00	Up	0	0
COL13A1	Collagen type xiii alpha 1 chain	169	641	3.79	Up	4.59E-68	1.73E-58
COL15A1	Collagen type xv alpha 1 chain	7282	16136	2.22	Up	0	0
COL9A2	Collagen type ix alpha 2 chain	299	1865	6.24	Up	1.22E-294	1.12E-285
CORO2B	Coronin 2B	936	4637	4.95	Up	0	0
CORO6	Coronin 6	106	374	3.53	Up	1.65E-34	4.70E-26
CPEB1	Cytoplasmic polyadenylation element binding protein 1	996	3273	3.29	Up	0	0
CPEB4	Cytoplasmic polyadenylation element binding protein 4	4249	11137	2.62	Up	0	0
CPT1C	Carnitine palmitoyltransferase 1C	141	1370	9.72	Up	1.38E-267	1.19E-258
CPVL	Carboxypeptidase. Vitellogenic-like	4724	26083	5.52	Up	0	0
CRELD1	Cysteine rich with egf like domains 1	659	1469	2.23	Up	1.16E-77	4.70E-69
CTNNAL1	Catenin alpha like 1	6064	64067	10.57	Up	0	0
CTSA	Cathepsin A	16134	41448	2.57	Up	0	0
CTSB	Cathepsin B	15476	78172	5.05	Up	0	0
CTTNBP2	Cortactin-binding protein 2	2757	8384	3.04	Up	0	0
CXCL9	Chemokine 9	243	652	2.68	Up	1.50E-45	4.73E-36
CYTH4	Cytohesin 4	259	563	2.17	Up	1.02E-25	2.62E-17
DAAM2	Dishevelled associated activator of morphogenesis 2	1622	10105	6.23	Up	0	0
DCLK2	Doublecortin like kinase 2	188	387	2.06	Up	3.21E-15	6.97E-06
DCT	Dopachrom-tautomerase	616	52091	84.56	Up	0	0
DENND5A	Denn domain containing 5a	6641	16955	2.55	Up	0	0
DENND5B	Denn domain containing 5b	322	2264	7.03	Up	0	0
DFNA5	Gasdemin 5	1450	26427	18.23	Up	0	0
DHPS	Deoxyhypusine synthase	3738	6991	1.87	Up	2.14E-267	1.84E-257
DIXDC1	Dix domain containing 1	418	1367	3.27	Up	1.72E-126	9.14E-118
DLGAP1	Dlg associated protein 1	78	557	7.14	Up	7.84E-95	3.46E-83
DMXL2	Dmx like 2	200	1882	9.41	Up	0	0
DNAH5	Dynein axonemal heavy chain 5	15	290	19.33	Up	8.61E-67	3.21E-57
DNAH9	Dynein axonemal heavy chain 9	28	714	25.50	Up	4.56E-179	2.97E-169
DNAJC18	Dnaj heat shock protein family (hsp40) member c18	650	1555	2.39	Up	1.39E-94	6.18E-85
DNER	Delta/notch like egf repeat containing	842	9008	10.70	Up	0	0
DNM3	Dynamin 3	156	1421	9.11	Up	1.54E-270	1.33E-260
DOCK10	Dedicator of cytokinesis 10	1375	4942	3.59	Up	0	0
DOCK11	Dedicator of cytokinesis 11	1240	3624	2.92	Up	4.00E-305	3.71E-290
DOCK2	Dedicator of cytokinesis 2	535	1660	3.10	Up	2.87E-146	1.65E-136
DOK2	Docking protein 2	596	1258	2.11	Up	4.30E-60	1.52E-50
DST	Dystonin	16980	39656	2.34	Up	0	0
DTNA	Dystrobrevin alpha	450	1438	3.20	Up	9.14E-131	4.92E-121
DZIP3	Daz interacting zinc finger protein 3	1094	2602	2.38	Up	8.82E-160	5.31E-149
EDIL3	Egf like repeats and discoidin domains 3	420	798	1.90	Up	7.10E-29	1.87E-19
EDNRB	Endothelin receptor type B	351	2557	7.28	Up	0	0
EEF1A2	Eukaryotic translation elongation factor 1 alpha 2	399	67798	169.92	Up	0	0
EFCAB6	Ef-hand calcium binding domain 6	45	178	3.96	Up	3.19E-18	7.21E-08
EHBP1	Eh domain binding protein 1	4388	18259	4.16	Up	0	0
EHD3	Eh domain containing 3	130	932	7.17	Up	8.01E-158	4.81E-148

EIF4A2	Eukaryotic translation initiation factor 4a2	16850	57503	3.41	Up	0	0
ELP2	Elongator acetyltransferase complex subunit 2	1958	4276	2.18	Up	4.85E-226	3.73E-216
EML5	Emap like 5	446	1110	2.49	Up	3.94E-71	1.52E-61
ENHO	Energy homeostasis associated	244	506	2.07	Up	2.56E-21	6.16E-12
ENO2	Enolase 2	297	5636	18.98	Up	0	0
ENPP2	Ectonucleotide pyrophosphatase/phosphodiesterase 2	2998	24586	8.20	Up	0	0
ENPP3	Ectonucleotide pyrophosphatase/phosphodiesterase 3	41	239	5.83	Up	1.65E-32	4.58E-24
ENPP6	Ectonucleotide pyrophosphatase/phosphodiesterase 6	59	852	14.44	Up	1.28E-187	8.69E-179
EPB41L3	Erythrocyte membrane protein band 4.1 like 3	454	1941	4.28	Up	1.98E-235	1.56E-226
EPB49	Dematin actin binding protein	588	1832	3.12	Up	1.55E-162	9.47E-153
EPHA3	Eph receptor a3	180	399	2.22	Up	9.93E-19	2.30E-09
EPHA5	Eph receptor a5	41	477	11.63	Up	2.48E-97	1.12E-87
EPHA6	Eph receptor a6	17	706	41.53	Up	4.30E-190	2.95E-180
ERC2	Elks/rab6-interacting/cast family member 2	186	456	2.45	Up	1.41E-25	3.62E-17
ESRRB	Estrogen related receptor beta	0	101	#DIV/0!	Up	1.03E-26	2.68E-18
ETV1	Ets variant transcription factor 1	251	983	3.92	Up	2.37E-109	1.14E-100
ETV4	Ets variant transcription factor 4	598	1181	1.97	Up	1.29E-47	4.18E-39
ETV5	Ets variant transcription factor 5	1150	4629	4.03	Up	0	0
FAM96B	Cytosolic iron-sulfur assembly component 2b	1120	3179	2.84	Up	5.02E-253	4.14E-243
FANCL	Fa complementation group l	441	1017	2.31	Up	5.80E-57	2.01E-47
FBLN5	Fibulin 5	4362	10063	2.31	Up	0	0
FBP2	Fructose-bisphosphatase 2	203	20033	98.68	Up	0	0
FBXL4	F-box and leucine rich repeat protein 4	1755	3790	2.16	Up	8.31E-196	5.83E-186
FBXO10	F-box protein 10	577	1083	1.88	Up	3.88E-39	1.15E-30
FCER1G	Fc epsilon receptor ig	4572	45220	9.89	Up	0	0
FERMT3	Ferm domain containing kindlin 3	1027	7490	7.29	Up	0	0
FES	Fes proto-oncogene. Tyrosine kinase	932	4185	4.49	Up	0	0
FEZ1	Fasciculation and elongation protein zeta 1	582	2607	4.48	Up	0	0
FGD2	Fyve. Rhogef and ph domain containing 2	299	601	2.01	Up	3.93E-24	9.80E-15
FGD4	Fyve. Rhogef and ph domain containing 4	456	1475	3.23	Up	4.23E-136	2.34E-126
FGD5	Fyve. Rhogef and ph domain containing 5	1133	2665	2.35	Up	1.11E-158	6.72E-150
FGF1	Fibroblast growth factor 1	64	362	5.66	Up	1.78E-50	5.89E-42
FGGY	Fggy carbohydrate kinase domain containing	923	2131	2.31	Up	4.77E-123	2.47E-113
FGL2	Fibrinogen like 2	1621	22625	13.96	Up	0	0
FGR	Fgr proto-oncogene. Src family tyrosine kinase	1208	2774	2.30	Up	1.42E-158	8.56E-150
FILIP1	Filamin a interacting protein 1	851	3273	3.85	Up	0	0
FKBP15	Fkbp prolyl isomerase family member 15	4393	8273	1.88	Up	0	0
FLRT3	Fibronectin leucine rich transmembrane protein 3	7891	16976	2.15	Up	0	0
FRMD5	Ferm domain containing 5	26	317	12.19	Up	1.45E-63	5.30E-55
FRMPD4	Ferm and pdz domain containing 4	244	703	2.88	Up	1.25E-53	4.26E-45
FSD1L	Fibronectin type iii and spry domain containing 1 like	23	151	6.57	Up	1.56E-21	3.76E-12
FTH1	Ferritin heavy chain 1	3605	29204	8.10	Up	0	0
FTL	Ferritin light chain	13602	196231	14.43	Up	0	0
FUCA1	Alpha-l-fucosidase 1	2078	4152	2.00	Up	2.22E-184	1.48E-173
FUT8	Fucosyltransferase 8	2487	7229	2.91	Up	0	0
FXR1	Fmr1 autosomal homolog 1	6133	12514	2.04	Up	0	0

FYB	Fyn binding protein 1	647	2210	3.42	Up	4.18E-219	3.14E-208
G6PC3	Glucose-6-phosphatase catalytic subunit 3	1474	2939	1.99	Up	2.42E-128	1.29E-118
G6S	Solute Carrier Family 1 Member 2 gene variant G6S	4551	23412	5.14	Up	0	0
GABRA3	Gamma-aminobutyric acid type a receptor subunit alpha3	1739	3963	2.28	Up	3.47E-226	2.68E-216
GALNT12	Polypeptide n-acetylgalactosaminyltransferase 12	1113	2202	1.98	Up	9.33E-94	4.14E-84
GALNT14	Polypeptide n-acetylgalactosaminyltransferase 14	51	880	17.25	Up	3.20E-204	2.31E-194
GAPDH	Glyceraldehyde-3-phosphate dehydrogenase	111650	233339	2.09	Up	0	0
GAPDHS	Glyceraldehyde-3-phosphate dehydrogenase. Spermatogenic	165	342	2.07	Up	2.81E-13	5.88E-05
GARNL3	Gtpase Activating Rap/rangap Domain Like 3	389	12767	32.82	Up	0	0
GATSL2	Gats protein-like 2 (gatsl2) pseudogene	260	533	2.05	Up	4.90E-22	1.19E-12
GBE1	1.4-alpha-glucan branching enzyme 1	1779	3489	1.96	Up	1.93E-146	1.11E-137
GBP2	Guanylate binding protein 2	271	658	2.43	Up	3.59E-39	1.06E-29
GCC2	Grip and coiled-coil domain containing 2	3338	6435	1.93	Up	5.23E-264	4.46E-254
GCGR	Glucagon receptor	76	2095	27.57	Up	0	0
GCNT1	Glucosaminyl (n-acetyl) transferase 1	388	1103	2.84	Up	5.63E-86	2.39E-76
GCNT7	Glucosaminyl (n-acetyl) transferase family member 7	264	830	3.14	Up	1.01E-71	3.94E-65
GDAP2	Ganglioside induced differentiation associated protein 2	426	1084	2.54	Up	7.03E-72	2.73E-62
GDF10	Growth differentiation factor 10	560	1066	1.90	Up	7.24E-40	2.16E-38
GDF11	Growth differentiation factor 11	64	366	5.72	Up	1.83E-51	6.11E-43
GFRA2	Gdnf family receptor alpha 2	58	3703	63.84	Up	0	0
GGA2	Golgi associated. Gamma adaptin ear containing. ARF Binding Protein 2	2033	4389	2.16	Up	4.40E-227	3.40E-217
GGT5	Gamma-glutamyltransferase 5	319	3551	11.13	Up	0	0
GGT7	Gamma-glutamyltransferase 7	780	2208	2.83	Up	3.27E-175	2.10E-164
GHDC	Gh3 domain containing	434	875	2.02	Up	6.62E-37	1.92E-27
GJB1	Gap junction protein beta 1	609	3868	6.35	Up	0	0
GLA	Galactosidase alpha	1879	13450	7.16	Up	0	0
GLRB	Glycine receptor beta	126	396	3.14	Up	5.78E-33	1.60E-23
GLS	Glutaminase	545	1192	2.19	Up	5.74E-62	2.05E-51
GLS2	Glutaminase 2	69	255	3.70	Up	2.28E-24	5.69E-15
GM2A	Ganglioside gm2 activator	2920	8102	2.77	Up	0	0
GNAI2	G protein subunit alpha i2	30993	60009	1.94	Up	0	0
GNPTAB	N-Acetylglucosamine-1-Phosphate Transferase Subunits Alpha and Beta	6235	27731	4.45	Up	0	0
GPM6B	Glycoprotein M6B	392	2407	6.14	Up	0	0
GPNMB	Glycoprotein nmb	19533	565436	28.95	Up	0	0
GPR19	G-protein coupled receptor 19	46	220	4.78	Up	2.34E-26	6.00E-18
GPR34	G-protein coupled receptor 34	200	669	3.35	Up	2.29E-62	8.25E-53
GPR75	G-protein coupled receptor 75	22	647	29.41	Up	7.46E-166	4.64E-156
GPR84	G-protein coupled receptor 84	7	108	15.43	Up	3.09E-21	7.42E-12
GPX1	Glutathione peroxidase 1	18072	34290	1.90	Up	0	0
GRAMD1A	GRAM domain-containing protein 1A	2619	11123	4.25	Up	0	0
GRAMD1B	GRAM domain-containing protein 1B	194	403	2.08	Up	2.38E-16	5.28E-07
GRID1	Glutamate ionotropic receptor delta type subunit 1	95	522	5.49	Up	2.97E-75	1.17E-64
GRIK5	Glutamate ionotropic receptor kainate type subunit 5	9	562	62.44	Up	5.60E-157	3.36E-147
GRINA	Glutamate Ionotropic Receptor NMDA type subunit associated protein 1	3556	10501	2.95	Up	0	0

GRN	Granulin precursor	26643	57395	2.15	Up	0	0
GRSF1	G-rich rna sequence binding factor 1	3967	9210	2.32	Up	0	0
GSTA4	Glutathione s-transferase alpha 4	2347	5990	2.55	Up	0	0
GUSB	Glucuronidase beta	4742	13249	2.79	Up	0	0
GYS1	Glycogen synthase 1	3749	7065	1.88	Up	3.35E-276	2.95E-265
HBA	Hemoglobin subunit alpha 1	215	417	1.94	Up	2.83E-14	6.03E-05
HCLS1	Hematopoietic cell-specific lyn substrate 1	1540	5334	3.46	Up	0	0
HCN1	Hyperpolarization activated cyclic nucleotide gated potassium channel 1	118	1724	14.61	Up	0	0
HECW2	Hect. C2 and ww domain containing e3 ubiquitin protein ligase 2	305	681	2.23	Up	7.63E-36	2.16E-25
HKDC1	Hexokinase domain containing 1	29	884	30.48	Up	4.01E-229	3.12E-219
HMGCS2	3-Hydroxy-3-Methylglutaryl-coa Synthase 2	0	201	#DIV/0!	Up	2.29E-59	8.05E-50
HPRT1	Hypoxanthine phosphoribosyltransferase 1	4305	14763	3.43	Up	0	0
HPS3	Hps3 biogenesis of lysosomal organelles complex 2 subunit 1	2376	21341	8.98	Up	0	0
HPSE	Heparanase	179	1187	6.63	Up	1.17E-192	8.11E-184
HS3ST2	Heparan sulfate-glucosamine 3-sulfotransferase 2	38	3636	95.68	Up	0	0
HSF4	Heat shock transcription factor 4	210	1780	8.48	Up	0	0
HSP90AA1	Heat shock protein 90 alpha family class a member 1	101321	216097	2.13	Up	0	0
HSPA12A	Heat shock protein family a (hsp70) member 12a	93	891	9.58	Up	5.80E-172	3.70E-162
HSPBAP1	Hspb1 associated protein 1	609	2519	4.14	Up	2.00E-304	1.85E-289
HUS1	Hus1 checkpoint clamp component	235	449	1.91	Up	5.35E-15	1.16E-05
IFFO1	Intermediate filament family orphan 1	676	1916	2.83	Up	5.00E-151	2.93E-141
IFIT1	Interferon induced protein with tetratricopeptide repeats 1	215	1363	6.34	Up	2.84E-217	2.13E-208
IFIT1B	Interferon induced protein with tetratricopeptide repeats 1b	114	381	3.34	Up	4.62E-34	1.30E-24
IFIT3	Interferon induced protein with tetratricopeptide repeats 3	326	785	2.41	Up	1.08E-45	3.44E-37
IFT52	Intraflagellar transport 52	2903	5970	2.06	Up	1.65E-280	1.48E-271
IFT74	Intraflagellar transport 74	1506	2823	1.87	Up	8.65E-107	4.12E-97
IGSF11	Immunoglobulin superfamily member 11	45	1248	27.73	Up	0	0
IGSF8	Immunoglobulin superfamily member 8	2074	4579	2.21	Up	3.08E-247	2.51E-237
IL4R	Interleukin-4 receptor	8583	18223	2.12	Up	0	0
IL8	Interleukin-8	16	358	22.38	Up	6.70E-86	2.84E-77
ILT11A	Immunoglobulin-like transcript 11 A	52	4462	85.81	Up	0	0
INSC	Spindle orientation adaptor protein	28	2521	90.04	Up	0	0
INTS8	Integrator complex subunit 8	3257	6149	1.89	Up	3.50E-240	2.80E-230
IP6K3	Inositol hexakisphosphate kinase 3	2	418	209.00	Up	1.11E-122	5.78E-114
IPO5	Importin 5	19331	41330	2.14	Up	0	0
IRF4	Interferon-regulatory factor 4	1618	4582	2.83	Up	0	0
IRF8	Interferon-regulatory factor 8	175	2576	14.72	Up	0	0
IRG1	Immunoresponsive gene 1	50	429	8.58	Up	2.32E-78	9.33E-68
ISL2	ISL LIM Homeobox 2; putative tumor suppressor	17	864	50.82	Up	1.23E-237	9.78E-229
ISM1	Isthmin 1	676	1478	2.19	Up	2.64E-76	1.05E-66
ITGA11	Integrin subunit alpha 11	1564	4083	2.61	Up	4.55E-290	4.15E-280
ITGA4	Integrin subunit alpha 4	251	649	2.59	Up	8.10E-43	2.49E-33
ITGAE	Integrin subunit alpha e	689	1922	2.79	Up	2.70E-149	1.56E-138
ITGAL	Integrin subunit alpha l	459	1089	2.37	Up	2.27E-64	8.28E-55
ITGB2	Integrin subunit beta 2	1178	6877	5.84	Up	0	0

ITGB3	Integrin subunit beta 3	321	907	2.83	Up	1.49E-69	5.68E-60
JAK3	Janus kinase 3. A thyrosine kinase	516	1041	2.02	Up	1.36E-43	4.26E-35
JAKMIP1	Janus kinase and microtubule interacting protein 1	98	261	2.66	Up	5.31E-16	1.17E-06
KAZN	Kazrin. Periplakin interacting protein	1935	4335	2.24	Up	3.03E-240	2.42E-230
KBTBD10	Kelch like family member 41	4	444	111.00	Up	3.32E-128	1.77E-118
KCNA6	Potassium voltage-gated channel subfamily a member 6	37	153	4.14	Up	5.20E-15	1.12E-05
KCNB2	Potassium voltage-gated channel subfamily b member 2	24	834	34.75	Up	4.13E-220	3.11E-210
KCNJ13	Potassium inwardly rectifying channel subfamily j member 13	0	656	#DIV/0!	Up	2.15E-203	1.54E-193
KCNJ8	Potassium inwardly rectifying channel subfamily j member 8	469	1054	2.25	Up	2.11E-56	7.26E-47
KHDRBS3	Kh rna binding domain containing. Signal transduction associated 3	238	5893	24.76	Up	0	0
KIF1A	Kinesin family member 1a	46	601	13.07	Up	3.14E-128	1.67E-118
KIF3A	Kinesin family member 3a	1271	2519	1.98	Up	4.07E-109	1.95E-98
KIF3C	Kinesin family member 3c	379	1571	4.15	Up	8.20E-186	5.51E-176
KIRREL3	Kirre like nephrin family adhesion molecule 3	35	480	13.71	Up	3.01E-103	1.41E-94
KIT	Kit proto-oncogene. Receptor tyrosine kinase	821	2586	3.15	Up	1.22E-232	9.59E-225
KLHDC8B	Kelch domain containing 8b	192	693	3.61	Up	2.94E-70	1.13E-60
KLHL25	Kelch like family member 25	1095	6570	6.00	Up	0	0
KLHL30	Kelch like family member 30	23	380	16.52	Up	2.52E-85	1.06E-75
KNG1	Kininogen 1	136	409	3.01	Up	2.99E-32	8.20E-24
KRT86	Keratin 86	5545	14398	2.60	Up	0	0
KYNU	Kynureninase	23	165	7.17	Up	3.76E-25	9.50E-16
LAMA2	Laminin subunit alpha 2	5947	11679	1.96	Up	0	0
LAMA4	Laminin subunit alpha 4	5305	13051	2.46	Up	0	0
LASS2	Ceramide synthase 2; tumor metastasis suppressor	4810	9432	1.96	Up	0	0
LCP1	Lymphocyte cytosolic protein 1	3435	19247	5.60	Up	0	0
LCP2	Lymphocyte cytosolic protein 2	789	1971	2.50	Up	1.75E-128	9.38E-120
LDHB	Lactate dehydrogenase b	11949	31771	2.66	Up	0	0
LDHD	Lactate dehydrogenase d	460	1620	3.52	Up	2.84E-164	1.76E-154
LGB1	Beta-lactoglobulin-1. Horse	48	457	9.52	Up	2.20E-87	9.39E-77
LGI4	Leucine rich repeat lgi family member 4	96	327	3.41	Up	2.06E-29	5.47E-20
LILRB3	Leukocyte immunoglobulin like receptor b3	103	2276	22.10	Up	0	0
LIMA1	Lim domain and actin binding 1	4621	8818	1.91	Up	0	0
LIMCH1	LIM and calponin homology domains 1; meta predictor in breast cancer	2180	8641	3.96	Up	0	0
LIPA	Lipase A; cholesterol ester hydrolase	490	10842	22.13	Up	0	0
LITAF	Lipopolysaccharide Induced TNF Factor; p53-inducible gene 7 (PIG7)	7128	48309	6.78	Up	0	0
LMBRD1	Lysosomal Cobalamin transport escort protein	4396	9052	2.06	Up	0	0
LMOD2	Leiomodin 2	12	141	11.75	Up	2.15E-28	5.52E-17
LONRF3	Lon peptidase n-terminal domain and ring finger 3	292	1563	5.35	Up	9.78E-225	7.49E-215
LPL	Lipoproteinlipase	190	1182	6.22	Up	5.77E-186	3.88E-177
LPXN	Leupaxin	320	1835	5.73	Up	1.90E-276	1.68E-266
LRRC48	Leucine rich repeat containing 48	173	360	2.08	Up	2.67E-14	5.70E-05
LRRC49	Leucine rich repeat containing 49	188	661	3.52	Up	4.77E-65	1.75E-55
LRRN2	Leucine rich repeat neuronal 2	300	1437	4.79	Up	1.01E-190	6.96E-181
LSAMP	Limbic system associated membrane protein	53	6937	130.89	Up	0	0
LY9	Lymphocyte antigen 9	146	2303	15.77	Up	0	0

LY96	Lymphocyte antigen 99	840	4219	5.02	Up	0	0
LZTR1	Leucine zipper like post translational regulator 1	2172	6985	3.22	Up	0	0
MAB21L1	Mab-21 like 1	227	801	3.53	Up	8.28E-80	3.39E-70
MAD1L1	Mitotic arrest deficient 1 like 1	1416	3116	2.20	Up	2.78E-166	1.73E-156
MAGED2	Melanoma-associated antigen D2	9100	21524	2.37	Up	0	0
MAK	Male germ cell associated kinase	89	242	2.72	Up	4.12E-15	8.93E-06
MAOB	Monoamine oxidase B	355	2987	8.41	Up	0	0
MARCH10	Member of the MARCH family of membrane-bound E3 ubiquitin ligases	3	103	34.33	Up	6.79E-24	1.69E-14
MBOAT1	Membrane bound o-acyltransferase domain containing 1	415	2771	6.68	Up	0	0
MBOAT2	Membrane bound o-acyltransferase domain containing 2	49	433	8.84	Up	3.86E-79	1.57E-69
MC1R	Melanocortin 1 receptor	149	434	2.91	Up	5.81E-33	1.61E-23
MCOLN1	Mucolipin trp cation channel 1	7376	17465	2.37	Up	0	0
MCOLN2	Mucolipin trp cation channel 2	452	4832	10.69	Up	0	0
MCOLN3	Mucolipin trp cation channel 3	67	3283	49.00	Up	0	0
ME2	Malic enzyme 2	1642	3668	2.23	Up	9.04E-202	6.46E-192
ME3	Malic enzyme 3	1652	6364	3.85	Up	0	0
MECR	Mitochondrial Trans-2-Enoyl-coa Reductase	1333	2667	2.00	Up	4.65E-117	2.33E-107
MED21	Mediator complex subunit 21	1612	3233	2.01	Up	2.73E-143	1.55E-133
MEF2C	Myocyte enhancer factor 2	3792	8282	2.18	Up	0	0
MEGF10	Multiple egf like domains 10	60	788	13.13	Up	2.01E-169	1.27E-159
MEX3B	Muscle excess 3RNA-binding family member B; confers resistance to cancer immunotherapy	242	852	3.52	Up	6.86E-85	2.89E-75
MFI2	Melanotransferrin	38	9092	239.26	Up	0	0
MFRP	Membrane frizzled-related protein	4	237	59.25	Up	9.34E-64	3.39E-54
MGAT4B	Alpha-1.3-mannosyl-glycoprotein 4-beta-n-acetylglucosaminyltransferase b	6774	13234	1.95	Up	0	0
MHCB3	Equus caballus MHC class I heavy chain	1596	17733	11.11	Up	0	0
MHCX1	MHC class I heavy chain (horse)	8622	18653	2.16	Up	0	0
MICAL1	Molecule interacting with casl 1; Calponin homologue	3381	17618	5.21	Up	0	0
MILL	MHC class I-like located near the leukocyte receptor complex	101	601	5.95	Up	1.63E-89	7.11E-81
MIPOL1	Mirror-image polydactyly 1	464	1147	2.47	Up	8.35E-73	3.26E-63
MITF	Microphthalmia-associated transcription factor; mel marker. High in proliferative HMM. Low in invase-type HMM	4278	69573	16.26	Up	0	0
MLH1	DNA mismatch repair protein	554	1550	2.80	Up	1.75E-118	8.88E-111
MLIP	Muscular lmna interacting protein	8	368	46.00	Up	1.55E-98	7.06E-89
MLPH	Melanophilin	3732	24913	6.68	Up	0	0
MME	Membrame metalloendopeptidase	362	17139	47.35	Up	0	0
MMP1	Matrix metalloproteinase 1	353	44222	125.27	Up	0	0
MMP3	Matrix metalloproteinase 3	469	930	1.98	Up	5.96E-39	1.74E-28
MMP9	Matrix metalloproteinase 9	224	991	4.42	Up	1.18E-121	6.08E-113
MORN1	Morn repeat containing 1	26	148	5.69	Up	4.36E-19	1.01E-09
MOSPD2	Motile sperm domain containing 2	2635	6527	2.48	Up	0	0
MPDZ	Multiple pdz domain crumbs cell polarity complex component	1787	3948	2.21	Up	4.98E-214	3.68E-203
MPEG1	Macrophage expressed 1	565	7507	13.29	Up	0	0
MPP1	Maguk p55 scaffold protein 1	2107	6685	3.17	Up	0	0
MPP6	Maguk p55 scaffold protein 6	136	3506	25.78	Up	0	0

MSR1	Macrophage scavenger receptor 1	404	4136	10.24	Up	0	0
MTHFD2	Methylenetetrahydrofolate dehydrogenase 2; controls effector and regulatory T cell fate and function	377	1647	4.37	Up	8.73E-204	6.29E-194
MTMR2	Myotubularin related protein 2	1657	4284	2.59	Up	2.00E-305	1.86E-290
MVP	Major vault protein	6867	19219	2.80	Up	0	0
MYBPC1	Myosin binding protein c1	8	665	83.13	Up	1.60E-189	1.10E-180
MYH1	Myosin heavy chain 1	18	632	35.11	Up	3.36E-166	2.09E-156
MYH2	Myosin heavy chain 2	4	2690	672.50	Up	0	0
MYLK2	Myosin light chain kinase 2	3	73	24.33	Up	7.93E-15	1.71E-05
MYO16	Myosin 16	48	197	4.10	Up	3.48E-20	8.23E-11
MYO1G	Myosin IG	871	1679	1.93	Up	1.28E-65	4.76E-57
MYOT	Myosin T	104	404	3.88	Up	1.10E-42	3.37E-33
MYPN	Myopalladin	18	159	8.83	Up	7.37E-28	1.90E-17
NAGLU	N-acetyl-alpha-D-glucosaminide	2962	5781	1.95	Up	1.38E-242	1.11E-233
NAIP	Nlr family apoptosis inhibitory protein	33	313	9.48	Up	5.26E-58	1.83E-48
NAP1L1	Nucleosome assembly protein 1 like 1	17156	45355	2.64	Up	0	0
NAP1L3	Nucleosome assembly protein 1 like 3	223	654	2.93	Up	8.10E-52	2.70E-42
NAPEPLD	N-acyl phosphatidylethanolamine phospholipase d	167	679	4.07	Up	3.53E-78	1.42E-67
NBEA	Neurobeachin	746	2165	2.90	Up	1.97E-175	1.28E-166
NCAM1	Neural cell adhesion molecule 1	89	4106	46.13	Up	0	0
NCAM2	Neural cell adhesion molecule 2	23	228	9.91	Up	4.42E-42	1.35E-32
NCF2	Neutrophil cytosolic factor 2	386	3552	9.20	Up	0	0
NCF4	Neutrophil cytosolic factor 4	116	1588	13.69	Up	0	0
NCKAP1L	Nck associated protein 1 like	559	2225	3.98	Up	4.05E-256	3.36E-245
NEB	Nebulin	52	495	9.52	Up	7.75E-94	3.44E-84
NEDD1	Neural precursor cell expressed. Developmentally down-regulated 1	1816	3430	1.89	Up	8.29E-133	4.51E-123
NEDD4	Neural precursor cell expressed. Developmentally down-regulated 4	857	1743	2.03	Up	3.53E-79	1.43E-68
NEDD9	Neural precursor cell expressed. Developmentally down-regulated 9	1466	2835	1.93	Up	1.77E-114	8.80E-106
NFYB	Nuclear transcription factor y subunit beta	1126	2833	2.52	Up	1.62E-188	1.11E-179
NIPAL3	Nipa like domain containing 3	408	948	2.32	Up	1.33E-53	4.47E-44
NLK	Nemo like kinase	1155	2736	2.37	Up	5.54E-167	3.44E-156
NOTCH4	Neurogenic locus notch homologue protein 4	472	1233	2.61	Up	1.47E-85	6.22E-76
NPHP1	Nephrocystin 1	526	1314	2.50	Up	2.73E-86	1.15E-75
NR4A3	Nuclear receptor 4A3	77	10150	131.82	Up	0	0
NRCAM	Neuronal cell adhesion molecule	151	4361	28.88	Up	0	0
NRP2	Neuropilin 2	1574	3131	1.99	Up	3.21E-136	1.77E-126
NSF	N-Ethylmaleimide Sensitive Factor. Vesicle Fusing atpas	2993	8207	2.74	Up	0	0
NUP210	Nucleoporin 210	719	1389	1.93	Up	7.60E-56	2.59E-45
OAS2	2'-5'-oligoadenylate synthetase 2	1073	2270	2.12	Up	1.83E-110	8.90E-102
OCA2	Oculocutaneous Albinism type 2 melanosomalt transmembrane protein	67	688	10.27	Up	1.17E-135	6.44E-127
OGFRL1	Opioid growth factor receptor like 1	514	3147	6.12	Up	0	0
OMG	Oligodendrocyte myelin glycoprotein	145	666	4.59	Up	6.59E-85	2.76E-74
OSGIN1	Oxidative stress-induced growth inhibitor 1	699	3082	4.41	Up	0	0
OSGIN2	Oxidative stress-induced growth inhibitor 2	744	2951	3.97	Up	0	0
OTOA	Otoancorin	96	1434	14.94	Up	0	0

P2RX4	Purinergic receptor p2x 4	3127	8871	2.84	Up	0	0
P2RY2	Purinergic receptor p2y2	155	1331	8.59	Up	6.18E-247	5.03E-237
P4HTM	Prolyl 4-hydroxylase. Transmembrane	542	7092	13.08	Up	0	0
PABPC4	Poly(a) binding protein cytoplasmic 4	8235	19829	2.41	Up	0	0
PABPC5	Poly(a) binding protein cytoplasmic 5	0	90	#DIV/0!	Up	3.13E-25	7.81E-15
PACSIN1	Protein kinase c and casein kinase substrate in neurons 1	87	484	5.56	Up	3.50E-69	1.33E-60
PADI2	Peptidyl arginine deiminase 2	272	2238	8.23	Up	0	0
PAQR8	Progestin And adipoq Receptor Family Member 8	370	1595	4.31	Up	4.32E-195	3.01E-185
PARP14	Poly(adp-ribose) polymerase family member 14	457	978	2.14	Up	2.49E-48	7.97E-39
PATZ1	Poz/btb and at hook containing zinc finger 1	2948	14258	4.84	Up	0	0
PAX3	Paired box gene 3	55	3826	69.56	Up	0	0
PAX6	Paired box gene 6	28	209	7.46	Up	1.65E-32	4.58E-24
PCDH9	Protocadherin 9	94	714	7.60	Up	1.02E-122	5.31E-116
PCDHGB2	Protocadherin gamma subfamily b. 2	272	605	2.22	Up	2.37E-30	6.37E-21
PCDHGB5	Protocadherin gamma subfamily b. 5	226	466	2.06	Up	4.20E-20	9.77E-10
PCNXL2	Pecanex 2	100	816	8.16	Up	9.22E-148	5.31E-137
PDE1C	Phosphodiesterase 1C	272	1351	4.97	Up	5.62E-184	3.75E-174
PDE4D	Phosphodiesterase 4D	217	458	2.11	Up	9.14E-20	2.15E-10
PDE5A	Phosphodiesterase 5A	360	918	2.55	Up	1.38E-59	4.92E-51
PDGFD	Platelet derived growth factor d	859	1725	2.01	Up	3.15E-75	1.25E-65
PDZRN4	Pdz domain containing ring finger 4	287	695	2.42	Up	2.28E-41	6.91E-32
PELI2	Pellino e3 ubiquitin protein ligase family member 2	194	405	2.09	Up	1.20E-15	2.69E-08
PEPD	Peptidase D	3779	9974	2.64	Up	0	0
PFKFB3	6-phosphofructo-2-kinase/fructose-2.6-biphosphatase 3	1321	2934	2.22	Up	4.13E-159	2.49E-149
PFKM	Phosphofructokinase. Muscle	2601	10947	4.21	Up	0	0
PHACTR1	Phosphatase and actin regulator 1	179	586	3.27	Up	6.78E-53	2.27E-43
PHF16	Jade family phd finger 3	186	389	2.09	Up	6.38E-16	1.40E-06
PHIP	Pleckstrin homology domain interacting protein	4819	8989	1.87	Up	0	0
PHLDB1	Pleckstrin homology like domain family b member 1	2818	8340	2.96	Up	0	0
PICALM	Phosphatidylinositol binding clathrin assembly protein	7504	15387	2.05	Up	0	0
PIGZ	Phosphatidylinositol glycan anchor biosynthesis class z	84	293	3.49	Up	9.84E-27	2.53E-18
PIK3C3	Phosphatidylinositol 3-kinase catalytic subunit type 3	1416	2824	1.99	Up	2.90E-123	1.51E-114
PION	Gamma-secretase activating protein	347	1288	3.71	Up	4.60E-137	2.55E-127
PIP5K1B	Phosphatidylinositol-4-phosphate 5-kinase type 1 beta	420	2276	5.42	Up	0	0
PJA1	Praja ring finger ubiquitin ligase 1	1478	3126	2.12	Up	1.46E-153	8.69E-145
PKM2	Pyruvate kinase m1/2	65611	143027	2.18	Up	0	0
PKN3	Protein kinase n3	483	1001	2.07	Up	2.30E-45	7.25E-36
PKNOX2	Pbx/knotted 1 homeobox 2	243	878	3.61	Up	5.78E-90	2.51E-81
PLA2G15	Phospholipase a2 group xv	615	1563	2.54	Up	7.93E-106	3.73E-95
PLA2G6	Phospholipase a2 group vi	342	670	1.96	Up	1.22E-24	3.10E-16
PLAT	Plasminogen activator. Tissue type	988	2009	2.03	Up	1.52E-89	6.64E-81
PLCB2	Phospholipase c beta 2	541	1126	2.08	Up	6.66E-53	2.22E-42
PLCB4	Phospholipase c beta 4	2913	18185	6.24	Up	0	0
PLCL2	Phospholipase c like 2	761	2451	3.22	Up	4.83E-228	3.73E-217

PLD3	Phospholipase d family member 3	27824	84122	3.02	Up	0	0
PLD4	Phospholipase d family member 4	168	951	5.66	Up	1.28E-139	7.17E-131
PLEKHA5	Pleckstrin homology domain containing a5	6228	12633	2.03	Up	0	0
PLEKHO2	Pleckstrin homology domain containing o2	1247	7543	6.05	Up	0	0
PLOD3	Procollagen-lysine.2-oxoglutarate 5-dioxygenase 3	2937	9720	3.31	Up	0	0
PLRP2	Pancreatic lipase related protein 2 (gene/pseudogene)	19	157	8.26	Up	1.24E-24	3.16E-16
PLTP	Phospholipid transfer protein	9572	48817	5.10	Up	0	0
PLXDC1	Plexin domain containing 1	2329	6531	2.80	Up	0	0
PMEL17	Silv. Gp100	6400	732784	114.50	Up	0	0
PMP22	Peripheral myelin protein 22	3070	29024	9.45	Up	0	0
PNMA1	Pnma family member 1	268	735	2.74	Up	1.88E-53	6.34E-44
POLE4	Dna polymerase epsilon 4. Accessory subunit	1621	6553	4.04	Up	0	0
PORCN	Porcupine o-acyltransferase	442	1127	2.55	Up	4.29E-75	1.70E-65
POT1	Protection of telomeres 1	797	2118	2.66	Up	5.69E-153	3.36E-143
PPARGC1A	Pparg coactivator 1 alpha	165	857	5.19	Up	2.57E-119	1.30E-109
PPAT	Phosphoribosyl pyrophosphate amidotransferase	925	2372	2.56	Up	7.21E-163	4.44E-153
PPP1R3A	Protein phosphatase 1 regulatory subunit 3a	0	153	#DIV/0!	Up	3.57E-44	1.11E-34
PRAM1	Pml-rara regulated adaptor molecule 1	98	1304	13.31	Up	1.38E-282	1.24E-273
PRCP	Prolylcarboxypeptidase	2749	6544	2.38	Up	0	0
PRKG1	Protein kinase cgmp-dependent 1	308	2046	6.64	Up	0	0
PRMT2	Protein arginine methyltransferase 2	2639	5592	2.12	Up	7.79E-280	6.93E-270
PRUNE	Prune exopolyphosphatase 1	663	1415	2.13	Up	1.57E-71	5.99E-60
PSAP	Prosaposin	90707	272967	3.01	Up	0	0
PSAT1	Phosphoserine aminotransferase 1	1759	5277	3.00	Up	0	0
PTGDS	Prostaglandin d2 synthase	132	5736	43.45	Up	0	0
PTGIS	Prostaglandin i2 synthase	316	809	2.56	Up	2.42E-53	8.15E-44
PTGR2	Prostaglandin reductase 2	883	1744	1.98	Up	2.52E-73	9.85E-64
PTPDC1	Protein tyrosine phosphatase domain containing 1	154	356	2.31	Up	7.43E-18	1.69E-08
PTPN18	Protein tyrosine phosphatase non-receptor type 18	232	2051	8.84	Up	0	0
PTPN6	Protein tyrosine phosphatase non-receptor type 6	2430	4802	1.98	Up	1.71E-206	1.24E-197
PTPRC	Protein tyrosine phosphatase receptor type c	2346	5248	2.24	Up	1.13E-290	1.03E-280
PTPRD	Protein tyrosine phosphatase receptor type d	1450	2744	1.89	Up	3.96E-106	1.88E-96
PTPRE	Protein tyrosine phosphatase receptor type e	814	2788	3.43	Up	1.02E-276	9.05E-270
PTPRJ	Protein tyrosine phosphatase receptor type j	436	1323	3.03	Up	7.70E-113	3.78E-103
PTPRM	Protein tyrosine phosphatase receptor type m	1550	4158	2.68	Up	0	0
PYGM	Glycogen phosphorylase. Muscle associated	101	1000	9.90	Up	6.68E-196	4.69E-186
PYROXD2	Pyridine nucleotide-disulphide oxidoreductase domain 2	267	1748	6.55	Up	1.29E-283	1.16E-274
RAB11FIP1	Rab11 family interacting protein 1	3388	17834	5.26	Up	0	0
RAB11FIP5	Rab11 family interacting protein 5	2456	7062	2.88	Up	0	0
RAPSN	Receptor associated protein of the synapse	0	404	#DIV/0!	Up	1.26E-122	6.56E-114
RARB	Retinoic acid receptor beta	304	1147	3.77	Up	1.12E-122	5.85E-114
RASAL2	Ras protein activator like 2	2751	5509	2.00	Up	1.91E-244	1.55E-239
RASGRF2	Ras protein specific guanine nucleotide releasing factor 2	277	1676	6.05	Up	1.16E-259	9.77E-251
RASGRP3	Ras guanyl releasing protein 3	310	1194	3.85	Up	1.98E-131	1.07E-121
RASSF4	Ras association domain family member 4	210	728	3.47	Up	6.89E-71	2.65E-61
RASSF8	Ras association domain family member 8	1362	2769	2.03	Up	6.54E-126	3.44E-116

RDX	Radixin	4498	8548	1.90	Up	0	0
RFC3	Replication factor c subunit 3	991	3569	3.60	Up	0	0
RFTN1	Raftlin. Lipid raft linker 1	770	1531	1.99	Up	5.86E-66	2.15E-55
RFTN2	Raftlin. Lipid raft linker 2	1349	5194	3.85	Up	0	0
RFX4	Regulatory factor x4	29	138	4.76	Up	3.72E-15	8.08E-06
RGS7	Regulator of g protein signaling 7	42	683	16.26	Up	1.92E-154	1.14E-145
RMND1	Required for meiotic nuclear division 1 homolog	553	1060	1.92	Up	2.60E-40	7.77E-31
RNF13	Ring finger protein 13	7855	19407	2.47	Up	0	0
RNF157	Ring finger protein 157	658	2025	3.08	Up	2.39E-179	1.55E-167
RNF19B	Ring finger protein 19B	5449	10902	2.00	Up	0	0
RNFT2	Ring finger protein transmembrane 2	22	228	10.36	Up	7.76E-43	2.39E-33
RPA1	Replication protein a1	4464	8597	1.93	Up	0	0
RPS27L	Ribosomal protein s27 like	6708	16424	2.45	Up	0	0
RRAGD	Ras related gtp binding d	297	1501	5.05	Up	2.64E-207	1.92E-197
RRM2B	Ribonucleotide reductase regulatory tp53 inducible subunit m2b	123	387	3.15	Up	3.48E-32	9.56E-23
RSPO1	R-spondin 1	109	279	2.56	Up	3.49E-16	7.71E-08
RSPO2	R-spondin 2	11	1490	135.45	Up	0	0
RTTN	Rotatin	754	3574	4.74	Up	0	0
RUFY3	Run and fyve domain containing 3	3625	9742	2.69	Up	0	0
SAC3D1	Sac3 domain containing 1	662	1619	2.45	Up	2.89E-102	1.35E-92
SALL2	Sac3 domain containing 1	60	200	3.33	Up	3.40E-16	7.53E-07
SAMD9L	Sterile alpha motif domain containing 9 like	640	1448	2.26	Up	1.18E-78	4.81E-70
SCAND3	Scan domain containing 3	24	186	7.75	Up	4.97E-30	1.33E-20
SCG3	Secretogranin III	389	1481	3.81	Up	5.62E-162	3.43E-152
SCLY	Selenocysteine lyase	564	1150	2.04	Up	8.82E-52	2.91E-41
SCML4	Scm polycomb group protein like 4	23	734	31.91	Up	5.60E-191	3.86E-181
SCN7A	Sodium voltage-gated channel alpha subunit 7	77	294	3.82	Up	1.28E-28	3.40E-20
SCN9A	Sodium voltage-gated channel alpha subunit 9	30	437	14.57	Up	2.36E-96	1.06E-85
SCPEP1	Serine carboxypeptidase 1	9647	56932	5.90	Up	0	0
SCRN2	Secernin 2	1080	2337	2.16	Up	4.72E-120	2.41E-110
SCUBE2	Signal peptide. Cub domain and egf like domain containing 2	2980	5701	1.91	Up	1.76E-228	1.37E-219
SDCBP	Syndecan binding protein	5447	40780	7.49	Up	0	0
SDCCAG8	Shh signaling and ciliogenesis regulator sdccag8	312	700	2.24	Up	3.01E-36	8.65E-27
SDK2	Sidekick cell adhesion molecule 2	726	1706	2.35	Up	5.95E-101	2.75E-91
SEC14L1	SEC14-like peptide binding 1	3398	8396	2.47	Up	0	0
SEC14L2	SEC14-like peptide binding 2	787	2168	2.75	Up	8.26E-166	5.12E-155
SEC14L5	SEC14-like peptide binding 5	15	517	34.47	Up	5.63E-135	3.09E-125
SELPLG	Selectin p ligand; cd162	858	2867	3.34	Up	3.43E-277	3.04E-267
SEMA3D	Semaphorin 3D	286	1313	4.59	Up	2.01E-168	1.26E-158
SEMA4C	Semaphorin 4C	1733	3601	2.08	Up	2.90E-173	1.85E-162
SEMA6D	Semaphorin 6D	630	3593	5.70	Up	0	0
SEPP1	Selenoprotein P	30141	120156	3.99	Up	0	0
SERINC1	Serine incorporator 1	4264	13453	3.16	Up	0	0
SERPINB1	Serpin B1	2055	9201	4.48	Up	0	0
SESTD1	Sec14 and spectrin domain containing 1	2384	4634	1.94	Up	6.71E-193	4.65E-183
SEZ6L2	Seizure 6-like protein 2	73	408	5.59	Up	5.59E-58	1.94E-48
SGIP1	Sh3gl interacting endocytic adaptor 1)	147	639	4.35	Up	8.14E-78	3.27E-67

SH3BP4	Sh3 domain binding protein 4	3673	16825	4.58	Up	0	0
SHMT2	Serine hydroxymethyltransferase 2	2385	6568	2.75	Up	0	0
SIGLEC1	Sialic acid binding ig like lectin 1	191	412	2.16	Up	2.51E-18	5.76E-09
SIPA1L2	Signal induced proliferation associated 1 like 2	2437	5764	2.37	Up	0	0
SIRPA	Signal Regulatory Protein Alpha. Associated with Gioblastoma	2034	5056	2.49	Up	0	0
SKAP2	Src kinase associated phosphoprotein 2	422	1546	3.66	Up	5.11E-164	3.14E-159
SLAIN1	SLAIN motif family member 1. Novel stem cell gene	144	2562	17.79	Up	0	0
SLC11A1	Solute carrier family	211	582	2.76	Up	5.39E-42	1.64E-32
SLC13A4	Solute carrier family	33	685	20.76	Up	5.52E-165	3.42E-155
SLC13A5	Solute carrier family	7	390	55.71	Up	1.03E-105	4.88E-97
SLC15A3	Solute carrier family	540	5326	9.86	Up	0	0
SLC16A1	Solute carrier family	1502	17779	11.84	Up	0	0
SLC16A2	Solute carrier family	288	615	2.14	Up	2.08E-28	5.45E-19
SLC24A4	Solute carrier family	96	537	5.59	Up	2.13E-77	8.59E-68
SLC24A5	Solute carrier family	944	104996	111.22	Up	0	0
SLC28A3	Solute carrier family	2573	10917	4.24	Up	0	0
SLC2A4	Solute carrier family	163	2998	18.39	Up	0	0
SLC2A9	Solute carrier family	981	2057	2.10	Up	6.34E-99	2.90E-89
SLC35F1	Solute carrier family	34	1129	33.21	Up	2.84E-297	2.63E-287
SLC36A1	Solute carrier family	417	1828	4.38	Up	3.79E-227	2.93E-217
SLC36A2	Solute carrier family	438	842	1.92	Up	1.42E-30	3.87E-22
SLC37A1	Solute carrier family	270	1263	4.68	Up	2.87E-164	1.78E-154
SLC38A6	Solute carrier family	1064	3656	3.44	Up	0	0
SLC39A10	Solute carrier family	2128	9284	4.36	Up	0	0
SLC45A1	Solute carrier family	15	404	26.93	Up	8.86E-101	4.10E-91
SLC45A2	Solute carrier family	360	34552	95.98	Up	0	0
SLC4A2	Solute carrier family	8040	20790	2.59	Up	0	0
SLC6A17	Solute carrier family	135	563	4.17	Up	6.57E-65	2.41E-55
SLC6A8	Solute carrier family	3814	8863	2.32	Up	0	0
SLITRK2	Slit and ntrk like family member 2	217	922	4.25	Up	4.19E-110	2.03E-100
SMAD6	Smad family member 6	302	2856	9.46	Up	0	0
SMARCA1	Swi/snf related. Matrix associated. Actin dependent regulator of chromatin. Subfamily a. Member 1	1138	2540	2.23	Up	1.37E-137	7.66E-129
SMARCD3	Swi/snf related. Matrix associated. Actin dependent regulator of chromatin. Subfamily d. Member 3	1390	4287	3.08	Up	0	0
SNAP91	Runx family transcription factor 1	123	618	5.02	Up	3.84E-83	1.60E-73
SNCA	Synuclein alpha	212	33053	155.91	Up	0	0
SNX25	Sorting nexin 25	277	6425	23.19	Up	0	0
SNX8	Sorting nexin 8	1322	3684	2.79	Up	1.43E-287	1.29E-277
SOBP	Sine oculis binding protein homolog	284	676	2.38	Up	5.93E-40	1.75E-29
SOCS5	Suppressor of cytokine signaling 5	2341	4489	1.92	Up	4.75E-181	3.12E-171
SORBS1	Sorbin and sh3 domain containing 1	852	8738	10.26	Up	0	0
SORBS3	Sorbin and sh3 domain containing 3	5400	23884	4.42	Up	0	0
SORCS2	Sortilin related vps10 domain containing receptor 2	814	4927	6.05	Up	0	0
SOX10	SRY-box transcription factor 10; upregulated in proliferative HMM	457	15278	33.43	Up	0	0
SOX5	SRY-box transcription factor 5	177	612	3.46	Up	6.56E-59	2.30E-49

SOX6	SRY-box transcription factor 6	169	1079	6.38	Up	4.72E-173	3.01E-162
SPATA17	Spermatogenesis associated 17	106	355	3.35	Up	1.38E-31	3.76E-22
SPG20	Spartan; protein containing a MIT (Microtubule Interacting and Trafficking molecule) domain	3928	9304	2.37	Up	0	0
SPHK2	Sphingosine kinase 2	1457	2728	1.87	Up	8.39E-103	3.93E-93
SPRY2	Sprouty rtk signaling antagonist 2	1143	3168	2.77	Up	3.15E-244	2.55E-234
SPTBN1	Spectrin beta. Non-erythrocytic 1	38417	73343	1.91	Up	0	0
SQSTM1	Sequestosome 1	9622	22990	2.39	Up	0	0
ST3GAL1	St3 beta-galactoside alpha-2.3-sialyltransferase 1	203	757	3.73	Up	1.26E-78	5.16E-70
ST3GAL5	St3 beta-galactoside alpha-2.3-sialyltransferase 5	1615	7694	4.76	Up	0	0
STAC2	Sh3 and cysteine rich domain 2	505	1866	3.70	Up	4.66E-199	3.30E-189
STARD9	Star related lipid transfer domain containing 9	1607	3258	2.03	Up	8.35E-149	4.83E-138
STAT4	Signal transducer and activator of transcription 4	263	776	2.95	Up	1.39E-61	5.00E-54
STK10	Serine/threonine kinase 10	908	3433	3.78	Up	0	0
STK32A	Serine/threonine kinase 32a	30	153	5.10	Up	3.10E-18	7.11E-09
STK32C	Serine/threonine kinase 32c	84	442	5.26	Up	1.59E-59	5.66E-51
STK33	Serine/threonine kinase 33	60	418	6.97	Up	6.20E-69	2.34E-59
STOX1	Storkhead box 1	301	1115	3.70	Up	5.30E-118	2.68E-108
STRADB	Ste20 related adaptor beta	346	764	2.21	Up	1.22E-37	3.59E-29
STX2	Syntaxin 2	288	863	3.00	Up	2.29E-71	8.85E-63
STXBP1	Synthaxin-binding protein 1	953	4830	5.07	Up	0	0
SYNPO	Synaptopodin	3812	20028	5.25	Up	0	0
SYT17	Synaptotagmin 17	118	1192	10.10	Up	6.31E-236	4.98E-226
SYTL2	Synaptotagmin like 2	2869	14741	5.14	Up	0	0
SYTL4	Synaptotagmin like 4	672	1362	2.03	Up	6.09E-60	2.15E-50
SYTL5	Synaptotagmin like 5	159	2896	18.21	Up	0	0
TAGAP	T Cell Activation rhogtpase Activating Protein	534	3053	5.72	Up	0	0
TAOK2	Serine/threonine-protein kinase TAO2	5423	15844	2.92	Up	0	0
TAP2	Transporter 2. Atp binding cassette subfamily b member	1671	3746	2.24	Up	1.88E-208	1.37E-197
TASP1	Taspase 1	534	1100	2.06	Up	1.60E-50	5.23E-40
TBC1D1	Tbc1 domain family member 1	1790	6594	3.68	Up	0	0
TBC1D10C	Tbc1 domain family member 10c	161	499	3.10	Up	1.14E-40	3.45E-32
TBC1D16	Tbc1 domain family member 16	547	5311	9.71	Up	0	0
TBX15	T-box transcription factor 15	1177	11643	9.89	Up	0	0
TBXAS1	Thromboxane A synthase 1	343	855	2.49	Up	3.41E-54	1.16E-47
TES	Testin lim domain protein	3431	15166	4.42	Up	0	0
TEX9	Testis expressed 9	268	689	2.57	Up	3.01E-45	9.46E-36
TF	Transferrin	1970	51602	26.19	Up	0	0
TFAP2B	Transcription factor ap-2 beta	3721	13492	3.63	Up	0	0
TFEB	Transcription factor EB	1118	5228	4.68	Up	0	0
TGFB1	Transforming growth factor beta 1	2753	5338	1.94	Up	2.10E-222	1.59E-211
THNSL1	Threonine synthase like 1	138	572	4.14	Up	1.22E-64	4.52E-56
THNSL2	Threonine synthase like 2	121	301	2.49	Up	7.73E-17	1.73E-07
TIMM44	Translocase of inner mitochondrial membrane 44	2417	11386	4.71	Up	0	0
TIMP2	Tissue inhibitor of metalloproteinase 2	8958	20233	2.26	Up	0	0
TLN2	Talin 2	2646	10695	4.04	Up	0	0
TLR2	Toll-like receptor 2	2058	3866	1.88	Up	3.97E-148	2.30E-138
TLR6	Toll-like receptor 6	162	479	2.96	Up	1.75E-36	5.07E-28

TLR7	Toll-like receptor 7	84	929	11.06	Up	2.38E-189	1.62E-179
TLR8	Toll-like receptor 8	166	1142	6.88	Up	6.01E-190	4.11E-180
TM4SF19	Transmembrane 4 l six family member 19	138	970	7.03	Up	1.04E-161	6.41E-153
TM6SF2	Transmembrane 6 l six family member 2	71	374	5.27	Up	9.68E-51	3.19E-43
TM7SF3	Transmembrane 7 l six family member 3	1531	7028	4.59	Up	0	0
TM7SF4	Transmembrane 7 l six family member 4	11	278	25.27	Up	1.81E-66	6.78E-58
TMC6	Transmembrane channel like 6	1918	4890	2.55	Up	0	0
TMEFF2	Transmembrane protein with an EGF-like and two follistatin-like domains 2	2	544	272.00	Up	2.44E-165	1.51E-153
TMEM104	Transmembrane protein 104	1287	4104	3.19	Up	0	0
TMEM161A	Transmembrane protein 161A	831	2280	2.74	Up	1.63E-171	1.04E-162
TMEM185A	Transmembrane protein 185A	810	2246	2.77	Up	2.19E-173	1.40E-162
TMOD2	Tropomodulin 2	507	3009	5.93	Up	0	0
TMPRSS5	Transmembrane serine protease 5	12	162	13.50	Up	2.35E-33	6.46E-23
TMSB4X	Thymosin beta-4	71858	244792	3.41	Up	0	0
TNC	Tenascin C	12741	30235	2.37	Up	0	0
TNFRSF14	Tnf receptor superfamily member 14	1256	3457	2.75	Up	3.02E-264	2.58E-254
TNFSF12	Tnf superfamily member 12	1352	4063	3.01	Up	0	0
TNFSF13	Tnf superfamily member 13	1228	2291	1.87	Up	4.40E-86	1.86E-75
TOMM40L	Translocase of outer mitochondrial membrane 40 like	283	645	2.28	Up	3.65E-34	1.03E-24
TOX	Thymocyte selection associated high mobility group box	384	2341	6.10	Up	0	0
TPST1	Tyrosylprotein sulfotransferase 1	763	1476	1.93	Up	1.14E-57	3.99E-49
TRAF3IP3	TRAF3-interacting JNK-activating modulator	408	783	1.92	Up	5.28E-29	1.39E-19
TRDN	Triadin	103	323	3.14	Up	3.44E-26	8.79E-17
TRIM37	Tripartite motif 37	1377	5451	3.96	Up	0	0
TRIM63	Tripartite motif 63	118	4084	34.61	Up	0	0
TRPC1	Transient receptor potential cation channel subfamily c member 1	197	2969	15.07	Up	0	0
TRPM1	Transient receptor potential cation channel subfamily m member 1	615	45080	73.30	Up	0	0
TRPM2	Transient receptor potential cation channel subfamily m member 2	108	1245	11.53	Up	1.65E-259	1.39E-248
TRPV2	Transient receptor potential cation channel subfamily v member 2	1368	13419	9.81	Up	0	0
TSNARE1	T-snare domain containing 1	403	1314	3.26	Up	5.17E-122	2.66E-112
TTC18	Tetratricopetide repeat protein 18	156	748	4.79	Up	7.53E-98	3.42E-88
TTC32	Tetratricopetide repeat protein 32	601	1349	2.24	Up	6.47E-73	2.53E-63
TTC7B	Tetratricopetide repeat protein 78	906	3624	4.00	Up	0	0
TTC8	Tetratricopetide repeat protein 8	388	4009	10.33	Up	0	0
TUBA8	Tubulin alpha 8	1179	4320	3.66	Up	0	0
TUBB2A	Tubulin Beta 2A Class iia	26743	50537	1.89	Up	0	0
TUSC3	Tumor suppressor candidate 3	1178	2342	1.99	Up	5.29E-101	2.45E-92
TXNRD1	Thioredoxin reductase 1	2701	5537	2.05	Up	3.50E-259	2.95E-249
TYRP1	Tyrosinase related protein 1; 5.6-Dihydroxyindol-2-carbonsäure-Oxidase; The expression of TYRP1 is regulated by the microphthalmia-associated transcription factor (MITF).[19][20]	6250	1006937	161.11	Up	0	0
UAP1L1	Udp-n-acetylglucosamine pyrophosphorylase 1 like 1	1414	4773	3.38	Up	0	0
UCHL1	Ubiquitin c-terminal hydrolase l1	3971	28609	7.20	Up	0	0
UGCG	Udp-glucose ceramide glucosyltransferase	1295	4650	3.59	Up	0	0
UMODL1	Uromodulin like 1	34	184	5.41	Up	1.06E-23	2.63E-14

UNC80	Unc-80 homolog. Nalcn channel complex subunit	3	305	101.67	Up	1.87E-85	7.97E-77
USP18	Ubiquitin specific peptidase 18	49	373	7.61	Up	4.01E-63	1.45E-53
UTP14A	Utp14a small subunit processome component	2767	5565	2.01	Up	2.86E-252	2.35E-240
UXS1	Udp-glucuronate decarboxylase 1	1765	7132	4.04	Up	0	0
VAT1	Vesicle amine transport 1	13226	29902	2.26	Up	0	0
VAT1L	Vesicle amine transport 1 like	27	1178	43.63	Up	0	0
VLDLR	Very low density lipoprotein receptor	2055	3850	1.87	Up	1.51E-145	8.65E-137
VNN2	Vanin 2	395	1640	4.15	Up	2.21E-194	1.54E-184
VPS33A	Vacuolar protein sorting-associated protein 33a	3041	6032	1.98	Up	1.54E-263	1.31E-253
VWA5A	Von willebrand factor a domain containing 5a	2131	8179	3.84	Up	0	0
VWCE	Von willebrand factor c and egf domains	127	599	4.72	Up	9.85E-77	3.95E-67
WDR17	Wd repeat domain 17	114	277	2.43	Up	1.52E-14	3.26E-05
WDR44	Wd repeat domain 44	1320	3800	2.88	Up	0	0
WDR6	Wd repeat domain 6	3652	11777	3.22	Up	0	0
WDR66	Wd repeat domain 66	137	618	4.51	Up	1.67E-75	6.67E-67
WDR7	Wd repeat domain 7	1448	4505	3.11	Up	0	0
WDR96	Wd repeat domain 96	63	225	3.57	Up	2.73E-20	6.47E-11
WDTC1	Wd and tetratricopeptide repeats 1	4920	9620	1.96	Up	0	0
WNT8A	Wnt family member 8a	13	392	30.15	Up	1.68E-99	7.74E-90
WSCD1	Wsc domain containing 1	35	748	21.37	Up	1.48E-180	9.74E-172
XIRP2	Xin actin binding repeat containing 2	0	110	#DIV/0!	Up	1.46E-32	3.94E-21
ZBTB10	Zinc finger and btb domain containing 10	294	623	2.12	Up	2.39E-29	6.27E-19
ZBTB20	Zinc finger and btb domain containing 20	2011	4211	2.09	Up	4.10E-205	2.96E-195
ZBTB44	Zinc finger and btb domain containing 44	736	1663	2.26	Up	1.00E-90	4.40E-82
ZEB2	Zinc finger E-box-binding homeobox 2	2449	12127	4.95	Up	0	0
ZFHX3	Zinc finger homeobox protein 3	1320	2597	1.97	Up	9.48E-111	4.58E-100
ZFHX4	Zinc finger homeobox protein 4	221	1472	6.66	Up	1.76E-240	1.41E-231
ZFP106	Zinc finger protein 106	1516	6840	4.51	Up	0	0
ZFYVE16	Zinc finger FYVE domain-containing protein 16	1553	4737	3.05	Up	0	0
ZFYVE27	Zinc finger FYVE domain-containing protein 27	2079	4049	1.95	Up	6.47E-169	4.07E-159
ZKSCAN2	Zinc finger with krab and scan domain 2	313	605	1.93	Up	4.69E-22	1.13E-12
ZKSCAN4	Zinc finger with krab and scan domain 4	245	544	2.22	Up	7.35E-27	1.89E-17
ZMYND15	Zinc finger mynd-type containing 15	47	168	3.57	Up	2.80E-14	5.96E-05
ZNF187	Zinc finger C2H2-type containing 187	196	554	2.83	Up	3.07E-41	9.27E-32
ZNF311	Zinc finger C2H2-type containing 311	89	423	4.75	Up	1.51E-52	5.11E-44
ZNF323	Zinc finger C2H2-type containing 323	121	302	2.50	Up	5.21E-17	1.17E-07
ZNF365	Zinc finger C2H2-type containing 365	315	641	2.03	Up	1.01E-25	2.59E-17
ZNF536	Zinc finger C2H2-type containing 536	57	403	7.07	Up	6.72E-66	2.49E-56
ZNF546	Zinc finger C2H2-type containing 546	91	263	2.89	Up	2.04E-18	4.69E-09
ZNF568	Zinc finger C2H2-type containing 568	245	487	1.99	Up	2.18E-18	5.00E-10
ZNF729	Zinc finger C2H2-type containing 729	262	584	2.23	Up	2.62E-29	6.95E-20
ZNF770	Zinc finger C2H2-type containing 770	613	1145	1.87	Up	4.73E-41	1.43E-31
ZSCAN16	Zinc finger and scan domain containing 16	161	542	3.37	Up	2.84E-50	9.31E-41
ZSCAN2	Zinc finger and scan domain containing 2	150	352	2.35	Up	3.71E-18	8.51E-09
ZSCAN23	Zinc finger and scan domain containing 23	10	108	10.80	Up	1.01E-17	2.32E-09
ZSWIM1	Zinc finger swim-type containing 1	422	800	1.90	Up	8.67E-29	2.29E-19
