# Supplementary material for: Gray-Horse Melanoma—A Wolf in Sheep’s Clothing
Source: Int J Mol Sci. 2025 Jul 10;26(14):6620. doi: 10.3390/ijms26146620 (PMC12295847; doi:10.3390/ijms26146620)
Supplement: Supplementary file 1 [file ijms-26-06620-s001.zip › File S2-RNAseq-Downregulated.pdf]

Table S2: Genes with downregulated transcription in ghM compared to intact skin

Gene ID	Definition	Transcription		ghM sum/ skin sum	Regulation	P-value	FDR
		skin sum	ghM sum				
5HTT	Solute carrier family 6 member 4	537	199	0.37	Down	6.94E-27	1.79E-18
AADAC	Arylacetamide deacetylase	575	167	0.29	Down	2.12E-42	6.48E-33
ABCA1	Atp binding cassette subfamily a member 1	1218	307	0.25	Down	4.81E-110	2.33E-100
ABCA12	Atp binding cassette subfamily a member 12	5425	1433	0.26	Down	0	0
ABCB11	Atp binding cassette subfamily b member 11	306	74	0.24	Down	2.28E-26	5.84E-18
ABCB6	Atp binding cassette subfamily b member 6	3446	1254	0.36	Down	6.48E-196	4.55E-188
ABCD3	Atp binding cassette subfamily d member 3	10596	2902	0.27	Down	0	0
ABCG1	Atp binding cassette subfamily g member 1	1008	356	0.35	Down	6.25E-58	2.18E-48
ABHD2	Abhydrolase domain containing 2 acylglycerol lipase	12851	1217	0.09	Down	0	0
ABHD5	Abhydrolase domain containing 5 acylglycerol lipase	8188	2087	0.25	Down	0	0
ABHD6	Abhydrolase domain containing 6 acylglycerol lipase	9004	1211	0.13	Down	0	0
ABLIM3	Actin binding lim protein family member 3	1531	210	0.14	Down	3.60E-223	2.74E-213
ACAA1	Acetyl-coa Acyltransferase 1	10864	3825	0.35	Down	0	0
ACAA2	Acetyl-coa Acyltransferase 2	41945	18112	0.43	Down	0	0
ACACB	Acetyl-coa Carboxylase Beta	85177	4885	0.06	Down	0	0
ACAD10	Acyl-coa Dehydrogenase Family Member 10	3119	1387	0.44	Down	2.60E-120	1.33E-110
ACAN	Aggrecan	1108	83	0.07	Down	1.99E-209	1.46E-199
ACAP1	Arfgap With Coiled-Coil. Ankyrin Repeat And PH Domains 1	1929	650	0.34	Down	2.40E-122	1.24E-113
ACCSL	1-aminocyclopropane-1-carboxylate synthase homolog (inactive) like	7560	2345	0.31	Down	0	0
ACLY	Atp citrate lyase	40230	15425	0.38	Down	0	0
ACOX1	Acyl-coa Oxidase 1	8657	2643	0.31	Down	0	0
ACP6	Acid phosphatase 6. lysophosphatidic	2283	764	0.33	Down	7.37E-148	4.24E-137
ACPP	Acid phosphatase 3	5777	1224	0.21	Down	0	0
ACSL3	Acyl-coa Synthetase Long Chain Family Member 3	10781	3976	0.37	Down	0	0
ACSL5	Acyl-coa Synthetase Long Chain Family Member 5	10046	4061	0.40	Down	0	0
ACSM3	Acyl-coa Synthetase Medium Chain Family Member 3	4013	974	0.24	Down	0	0
ACSS2	Acyl-coa Synthetase Short Chain Family Member 2	4288	1858	0.43	Down	2.85E-177	1.85E-166
ACSS3	Acyl-coa Synthetase Short Chain Family Member 3	13102	1099	0.08	Down	0	0
ACTA2	Actin alpha 2. smooth muscle	22837	5942	0.26	Down	0	0
ACTC1	Actin alpha cardiac muscle 1	1347	194	0.14	Down	1.67E-189	1.15E-180
ACTG2	Actin gamma 2. smooth muscle	7647	743	0.10	Down	0	0
ACVR1B	Activin a receptor type 1b	3811	955	0.25	Down	0	0
ADA	Adenosine deaminase	8364	2091	0.25	Down	0	0
ADAM12	Adam metallopeptidase domain 12	1662	277	0.17	Down	6.89E-215	5.12E-205
ADAM15	Adam metallopeptidase domain 15	11003	2374	0.22	Down	0	0
ADAMTS1	Adam metallopeptidase with thrombospondin type 1 motif 1	6568	1275	0.19	Down	0	0
ADAMTS14	Adam metallopeptidase with thrombospondin type 1 motif 14	211	19	0.09	Down	1.51E-33	4.27E-25
ADAMTS18	Adam metallopeptidase with thrombospondin type 1 motif 18	155	4	0.03	Down	6.96E-35	1.95E-24
ADAMTS4	Adam metallopeptidase with thrombospondin type 1 motif 4	384	29	0.08	Down	3.45E-71	1.32E-60
ADAMTS5	Adam metallopeptidase with thrombospondin type 1 motif 5	511	232	0.45	Down	5.02E-17	1.11E-06
ADAMTS6	Adam metallopeptidase with thrombospondin type 1 motif 6	358	134	0.37	Down	1.90E-15	4.24E-08

ADAMTS9	Adam metallopeptidase with thrombospondin type 1 motif 9	878	50	0.06	Down	2.49E-179	1.63E-169
ADAMTSL1	Adamts like 1	658	268	0.41	Down	5.82E-28	1.52E-18
ADAMTSL2	Adamts like 2	704	267	0.38	Down	9.11E-35	2.57E-25
ADCK1	Aarf Domain Containing Kinase 1	647	297	0.46	Down	1.37E-19	3.26E-11
ADCK5	Aarf Domain Containing Kinase 5	2216	995	0.45	Down	9.95E-83	4.14E-73
ADCY4	Adenylate cyclase 4	262	59	0.23	Down	7.44E-24	1.85E-14
ADCY9	Adenylate cyclase 9	1968	887	0.45	Down	1.79E-71	6.97E-63
ADRB2	Adrenoceptor beta 2	737	215	0.29	Down	5.83E-55	1.99E-45
AFAP1	Actin filament associated protein 1	944	411	0.44	Down	6.29E-36	1.80E-26
AGPAT3	1-acylglycerol-3-phosphate o-acyltransferase 3	6934	781	0.11	Down	0	0
AHCYL2	Adenosylhomocysteinase like 2	1187	474	0.40	Down	3.13E-55	1.07E-45
AHR	Aryl hydrocarbon receptor	2928	1153	0.39	Down	1.37E-143	7.77E-135
AIM1	Crystallin beta-gamma domain containing 1	14603	2647	0.18	Down	0	0
ALDH18A1	Aldehyde dehydrogenase 18 family member a1	3901	1789	0.46	Down	6.88E-141	3.87E-131
ALDH1A3	Aldehyde dehydrogenase 1 family member a3	751	131	0.17	Down	2.48E-92	1.09E-82
ALDH1L2	Aldehyde dehydrogenase 1 family member l2	585	137	0.23	Down	2.81E-55	9.63E-46
ALDOC	Aldolase. fructose-bisphosphate c	21168	4064	0.19	Down	0	0
ALK	Alk receptor tyrosine kinase	121	13	0.11	Down	9.56E-17	2.14E-07
ALOX12	Arachidonate 12-lipoxygenase. 12s type	1389	292	0.21	Down	8.63E-151	5.04E-141
ALOX12B	Arachidonate 12-lipoxygenase. 12r type	5484	1476	0.27	Down	0	0
ALOX15B	Arachidonate 15-lipoxygenase type b	10088	1069	0.11	Down	0	0
ALOXE3	Arachidonate lipoxygenase 3	3535	1345	0.38	Down	2.22E-186	1.50E-176
AMMECR1L	Ammecr1 like	1090	487	0.45	Down	1.59E-38	4.71E-30
ANK3	Ankyrin 3	6852	2189	0.32	Down	0	0
ANKK1	Ankyrin repeat and kinase domain containing 1	2135	269	0.13	Down	0	0
ANKRD13A	Ankyrin repeat domain 13a	3881	1470	0.38	Down	1.56E-205	1.13E-196
ANKRD24	Ankyrin repeat domain 24	287	74	0.26	Down	9.38E-23	2.29E-13
ANKRD29	Ankyrin repeat domain 29	476	187	0.39	Down	7.31E-21	1.74E-11
ANKRD35	Ankyrin repeat domain 35	9991	1623	0.16	Down	0	0
ANO1	Anoctamin 1	4483	1353	0.30	Down	0	0
ANO9	Anoctamin 9	4030	485	0.12	Down	0	0
ANXA1	Annexin A1	81924	34365	0.42	Down	0	0
ANXA8L1	Annexin a8 like 1	19273	2558	0.13	Down	0	0
APBA2	Amyloid beta precursor protein binding family a member 2	427	51	0.12	Down	1.24E-63	4.55E-55
APOBEC3Z1B	Apolipoprotein B mrna-editing enzyme-catalytic polypeptide-like 3Z1b [(horse)	885	140	0.16	Down	5.45E-117	2.73E-107
AQP3	Aquaporin 3 (gill blood group)	50716	8057	0.16	Down	0	0
AR	Androgen receptor	250	45	0.18	Down	8.88E-29	2.31E-19
ARAP2	Arfgap With rhogap Domain. Ankyrin Repeat And PH Domain 2	1640	283	0.17	Down	5.88E-208	4.28E-197
ARFGEF2	Adp ribosylation factor guanine nucleotide exchange factor 2	2564	1029	0.40	Down	8.53E-122	4.39E-112
ARG2	Arginase 2	748	341	0.46	Down	8.93E-25	2.24E-15
ARHGAP11A	Rho gtpase Activating Protein 11A	1414	442	0.31	Down	5.33E-99	2.44E-89
ARHGAP27	Rho gtpase Activating Protein 27	2898	1213	0.42	Down	4.67E-127	2.47E-117
ARHGAP28	Rho gtpase Activating Protein 28	305	80	0.26	Down	6.78E-25	1.68E-14
ARHGAP33	Rho gtpase Activating Protein 33	1054	279	0.26	Down	7.54E-90	3.28E-80
ARHGEF15	Rho gtpase Activating Protein 15	1479	335	0.23	Down	2.45E-149	1.43E-139
ARHGEF16	Rho gtpase Activating Protein 16	758	150	0.20	Down	1.51E-83	6.34E-75

ARHGEF19	Rho gtpase Activating Protein 19	4396	750	0.17	Down	0	0
ARHGEF3	Rho gtpase Activating Protein 3	2288	823	0.36	Down	1.48E-130	8.02E-122
ARHGEF4	Rho gtpase Activating Protein 4	1429	465	0.33	Down	1.39E-93	6.18E-85
ARNTL	Basic helix-loop-helix arnt like 1	2031	632	0.31	Down	1.97E-143	1.12E-134
ARRB1	Arrestin beta 1	1363	633	0.46	Down	1.04E-44	3.28E-37
ARRDC4	Arrestin domain containing 4	841	321	0.38	Down	1.12E-40	3.39E-32
ARSF	Arylsulfatase F	18385	4821	0.26	Down	0	0
ASXL2	Asxl transcriptional regulator 2	2087	848	0.41	Down	3.24E-96	1.46E-86
ATAD2	Atpase Family AAA Domain Containing 2	2065	832	0.40	Down	9.41E-97	4.24E-87
ATF6	Activating transcription factor 6	4375	2035	0.47	Down	4.18E-153	2.47E-143
ATG4D	Autophagy related 4d cysteine peptidase	2989	1149	0.38	Down	2.73E-154	1.62E-144
ATG9B	Autophagy related 9b cysteine peptidase	4928	1264	0.26	Down	0	0
ATP10A	Atpase Phospholipid Transporting 10A	1151	389	0.34	Down	2.08E-71	8.06E-62
ATP12A	Atpase Phospholipid Transporting 12A	1347	278	0.21	Down	1.77E-146	1.02E-138
ATP13A5	Atpase 13A5	1446	249	0.17	Down	2.22E-183	1.47E-172
ATP2A2	Atpase Sarcoplasmic/Endoplasmic Reticulum Ca2+ Transporting 2	12207	4579	0.38	Down	0	0
ATP2A3	Atpase Sarcoplasmic/Endoplasmic Reticulum Ca2+ Transporting 3	3679	1078	0.29	Down	2.54E-285	2.29E-275
ATP2B4	Atpase Plasma Membrane Ca2+ Transporting 4	8608	3478	0.40	Down	0	0
ATP6V0A2	Atpase H+ Transporting V0 Subunit A2	5920	2228	0.38	Down	0	0
ATP6V0A4	Atpase H+ Transporting V0 Subunit A4	1394	144	0.10	Down	8.83E-235	6.93E-224
ATP6V1B1	Atpase H+ Transporting V1 Subunit B1	184	6	0.03	Down	1.96E-38	5.81E-30
ATP6V1C2	Atpase H+ Transporting V1 Subunit C2	6125	1517	0.25	Down	0	0
ATP8A2	Atpase Phospholipid Transporting 8A2	421	91	0.22	Down	4.95E-42	1.51E-32
ATP8B1	Atpase Phospholipid Transporting 8B1	1868	381	0.20	Down	9.25E-208	6.75E-198
ATP9A	Atpase Phospholipid Transporting 9A	3410	1330	0.39	Down	6.91E-172	4.40E-162
ATRIP	Atr interacting protein	1202	554	0.46	Down	1.05E-39	3.16E-31
ATXN7	Ataxin 7	1030	429	0.42	Down	1.45E-42	4.50E-35
AUTS2	Activator of transcription and developmental	10617	3324	0.31	Down	0	0
AVPR1A	Arginine vasopressin receptor 1a	646	141	0.22	Down	1.11E-64	4.10E-56
B4GALNT3	Beta-1.4-n-acetyl-galactosaminyltransferase 3	1120	189	0.17	Down	1.44E-141	8.18E-133
B4GALNT4	Beta-1.4-n-acetyl-galactosaminyltransferase 4	1032	131	0.13	Down	6.54E-156	3.90E-146
BAAT	Bile Acid-coa:Amino Acid N-Acyltransferase	708	43	0.06	Down	1.88E-141	1.06E-131
BACH1	Btb domain and cnc homolog 1	929	419	0.45	Down	1.93E-31	5.32E-23
BACH2	Btb domain and cnc homolog 2	258	68	0.26	Down	1.47E-18	3.44E-10
BAI2	Adhesion g protein-coupled receptor b2	534	216	0.40	Down	2.17E-22	5.26E-13
BBOX1	Gamma-butyrobetaine hydroxylase 1	906	198	0.22	Down	2.59E-95	1.15E-83
BCAM	Basal cell adhesion molecule (lutheran blood group)	19841	1828	0.09	Down	0	0
BCAT2	Branched chain amino acid transaminase 2	7740	2076	0.27	Down	0	0
BCL6B	Bcl6b transcription repressor	230	43	0.19	Down	1.73E-23	4.33E-15
BCL9L	Bcl9 like	10984	3670	0.33	Down	0	0
BD-1	Defensin beta 1	5116	230	0.04	Down	0	0
BEND7	Ben domain containing 7	692	303	0.44	Down	4.24E-25	1.07E-15
BICD2	Bicd cargo adaptor 2	5345	1862	0.35	Down	0	0
BIRC5	Baculoviral iap repeat containing 5	464	126	0.27	Down	1.41E-35	4.07E-27
BLMH	Bleomycin hydrolase	18217	7548	0.41	Down	0	0
BMP2	Bone morphogenetic protein 2	3402	1244	0.37	Down	7.83E-192	5.40E-183
BMP7	Bone morphogenetic protein 7	3159	1089	0.34	Down	1.84E-195	1.29E-185

BMPR1A	Bone morphogenetic protein receptor type 1a	2035	578	0.28	Down	1.38E-161	8.48E-154
BOC	Boc cell adhesion associated. oncogene regulated	11014	3407	0.31	Down	0	0
BPI	Bactericidal permeability increasing protein	300	42	0.14	Down	1.49E-39	4.46E-31
BPIL2	Bpi fold containing family c	7663	2002	0.26	Down	0	0
BRAF	B-raf proto-oncogene. serine/threonine kinase	1707	539	0.32	Down	1.65E-118	8.38E-109
BRD1	Bromodomain containing 1	4406	1841	0.42	Down	2.02E-195	1.41E-185
BRWD3	Bromodomain and wd repeat domain containing 3	1663	672	0.40	Down	7.28E-77	2.93E-67
BTBD11	Ankyrin repeat and btb domain containing 3	1979	483	0.24	Down	7.95E-187	5.36E-177
BTC	Betacellulin	200	46	0.23	Down	5.55E-17	1.25E-07
BUB1	Mitotic checkpoint serine/threonine kinase	1196	275	0.23	Down	1.83E-117	9.25E-109
BUB1B	Bub1 mitotic checkpoint serine/threonine kinase b	1706	467	0.27	Down	7.30E-142	4.13E-132
C25H9orf100	Rho guanine nucleotide exchange factor 39	270	81	0.30	Down	2.96E-17	6.68E-08
C2CD2	C2 calcium dependent domain containing 2	3365	757	0.22	Down	0	0
C9H8orf34	Chromosome 9 c8orf34 homolog	223	14	0.06	Down	9.69E-42	2.94E-32
CA6	Carbonic anhydrase 6	7728	1845	0.24	Down	0	0
CACHD1	Cache domain containing 1	5430	1931	0.36	Down	0	0
CACNA1C	Calcium voltage-gated channel subunit alpha1 C	664	294	0.44	Down	2.69E-24	6.63E-14
CACNA1G	Calcium voltage-gated channel subunit alpha1 G	681	134	0.20	Down	5.52E-76	2.20E-66
CALCB	Calcitonin related polypeptide beta	1022	467	0.46	Down	7.19E-35	2.03E-25
CALCRL	Calcitonin receptor like receptor	260	82	0.32	Down	3.12E-15	6.77E-06
CAMK1D	Calcium/calmodulin dependent protein kinase ID	1239	368	0.30	Down	1.92E-91	8.45E-83
CAMKK1	Calcium/calmodulin dependent protein kinase kinase 1	1186	414	0.35	Down	4.88E-70	1.87E-60
CAPSL	Calcyphosine like	271	17	0.06	Down	1.66E-50	5.51E-42
CARD10	Caspase recruitment domain family member 10	2894	329	0.11	Down	0	0
CARD14	Caspase recruitment domain family member 14	1220	238	0.20	Down	1.26E-139	7.04E-130
CARNS1	Carnosine synthase 1	1459	422	0.29	Down	3.85E-113	1.90E-103
CASC5	Cancer susceptibility candidate 5	772	199	0.26	Down	6.44E-67	2.41E-57
CASK	Calcium/calmodulin dependent serine protein kinase	5609	2121	0.38	Down	1.00E-291	9.31E-291
CASKIN2	CASK interacting protein 2	2949	1044	0.35	Down	7.38E-175	4.76E-165
CASZ1	Castor zinc finger 1	9665	1591	0.16	Down	0	0
CBFA2T2	CBFA2/RUNX1 partner transcriptional co-repressor 2	475	199	0.42	Down	5.00E-18	1.14E-08
CCDC120	Coiled-coil domain containing 120	4037	853	0.21	Down	0	0
CCDC6	Coiled-coil domain containing 6	4640	1963	0.42	Down	8.21E-202	5.85E-191
CCNA2	Cyclin A2	1181	435	0.37	Down	9.25E-64	3.36E-54
CCNB2	Cyclin B2; up-regulated in mel-meta	1239	293	0.24	Down	1.55E-118	7.91E-110
CCNE1	Cyclin E1	852	213	0.25	Down	1.10E-75	4.40E-67
CCR2	C-c motif chemokine receptor 2	332	79	0.24	Down	2.06E-29	5.47E-20
CD101	Cluster of differentiation 101	633	225	0.36	Down	8.99E-35	2.54E-25
CD109	Cluster of differentiation 109. Activated T-Cell Marker	6572	1760	0.27	Down	0	0
CD79A	Cluster of differentiation 79A. Immunoglobulin-Associated Alpha	545	253	0.46	Down	2.80E-16	6.20E-07
CD93	Cluster of differentiation 93	383	79	0.21	Down	1.31E-38	3.90E-30
CDC20	Cell division cycle 20	3411	516	0.15	Down	0	0
CDC45	Cell division cycle 45	450	106	0.24	Down	1.67E-40	5.06E-32
CDCA2	Cell division cycle associated 2	440	117	0.27	Down	2.12E-35	6.02E-26
CDCA7	Cell division cycle 7	1092	109	0.10	Down	3.54E-185	2.37E-175
CDCA7L	Cell Division Cycle 7 like	1405	602	0.43	Down	4.71E-57	1.63E-47
CDCP1	Cub domain containing protein 1	2330	219	0.09	Down	0	0

CDH2	Cadherin 2	229	27	0.12	Down	2.74E-33	7.60E-24
CDH20	Cadherin 20	177	13	0.07	Down	9.59E-31	2.59E-21
CDH23	Cadherin 23	769	67	0.09	Down	9.07E-137	5.02E-127
CDH24	Cadherin 24	573	95	0.17	Down	2.99E-72	1.17E-62
CDK14	Cyclin-dependent kinase 14	726	332	0.46	Down	9.39E-25	2.33E-14
CDKN2B	Cyclin dependent kinase inhibitor 2B	396	103	0.26	Down	2.30E-32	6.34E-23
CDS1	CDP-diacylglycerol synthase 1	4243	806	0.19	Down	0	0
CDT1	Chromatin licensing and DNA replication factor 1	595	160	0.27	Down	2.09E-48	6.77E-39
CELSR2	Cadherin EGF LAG seven-pass G-type receptor 2	14868	2628	0.18	Down	0	0
CENPE	Centromere protein E	1628	491	0.30	Down	4.72E-120	2.41E-111
CEP55	Centrosomal protein 55	519	204	0.39	Down	4.97E-24	1.22E-14
CEP72	Centrosomal protein 72	632	99	0.16	Down	3.73E-84	1.56E-73
CFB	Complement factor B	678	107	0.16	Down	5.23E-90	2.25E-79
CFTR	CF transmembrane conductance regulator	1982	195	0.10	Down	0	0
CHFR	Checkpoint with forkhead and ring finger domains	4092	1630	0.40	Down	5.81E-199	4.12E-189
CHI3L1	Chitinase 3 like 1	1067	179	0.17	Down	3.17E-136	1.76E-134
CHL1	Cell adhesion molecule L1 like	2170	529	0.24	Down	2.17E-205	1.57E-195
CHML	CHM like Rab escort protein	1072	365	0.34	Down	1.57E-64	5.80E-56
CHRM3	Cholinergic receptor muscarinic 3	125	11	0.09	Down	3.49E-20	8.12E-10
CHRNA4	Cholinergic receptor nicotinic alpha 4 subunit	66	0	0.00	Down	1.37E-13	2.93E-05
CHST9	Carbohydrate sulfotransferase 9	80	2	0.03	Down	1.10E-14	2.41E-06
CIITA	Class II major histocompatibility complex transactivator	781	205	0.26	Down	2.24E-66	8.31E-58
CIT	Citron rho-interacting serine/threonine kinase	2358	550	0.23	Down	2.37E-233	1.85E-223
CKAP2	Cytoskeleton associated protein 2	1266	387	0.31	Down	5.06E-91	2.21E-81
CKAP4	Cytoskeleton associated protein 4	3627	1265	0.35	Down	7.44E-221	5.62E-211
CKMT1B	Creatine kinase. mitochondrial 1B	11249	1677	0.15	Down	0	0
CLCA1	Chloride channel accessory 1	8177	84	0.01	Down	0	0
CLCA2	Chloride channel accessory 2	37890	9126	0.24	Down	0	0
CLCA4	Chloride channel accessory 4	2150	590	0.27	Down	4.57E-179	2.98E-169
CLEC16A	C-type lectin domain containing 16A	5907	2576	0.44	Down	1.33E-240	1.06E-231
CLIP4	CAP-Gly domain containing linker protein family member	5586	595	0.11	Down	0	0
CLMP	CXADR like membrane protein	2458	918	0.37	Down	6.44E-133	3.50E-123
CLSPN	Claspin	719	273	0.38	Down	1.68E-35	4.78E-26
CLSTN1	Calsyntenin 1	23752	8135	0.34	Down	0	0
CNKSR1	Connector enhancer of kinase suppressor of Ras 1	2027	161	0.08	Down	0	0
CNOT6	CCR4-NOT transcription complex subunit 6	1215	561	0.46	Down	5.40E-41	1.63E-31
CNOT6L	CCR4-NOT transcription complex subunit 6 like	3791	1597	0.42	Down	5.77E-165	3.58E-162
CNST	Consortin. connexin sorting protein	1297	444	0.34	Down	2.74E-80	1.12E-69
COBL	Cordon-bleu WH2 repeat protein	4090	820	0.20	Down	0	0
COBLL1	Cordon-bleu WH2 repeat protein like 1	10543	3678	0.35	Down	0	0
COL12A1	Collagen type xii alpha 1 chain	16740	6566	0.39	Down	0	0
COL16A1	Collagen type xvi alpha 1 chain	15096	6538	0.43	Down	0	0
COL17A1	Collagen type xvii alpha 1 chain	59242	7375	0.12	Down	0	0
COL1A1	Collagen type i alpha 1 chain	1163414	174632	0.15	Down	0	0
COL1A2	Collagen type i alpha 2 chain	595262	142363	0.24	Down	0	0
COL28A1	Collagen type xxviii alpha 1 chain	534	243	0.46	Down	8.52E-18	1.91E-07
COL3A1	Collagen type iii alpha 1 chain	800707	202303	0.25	Down	0	0

COL5A2	Collagen type v alpha 2 chain	39352	17257	0.44	Down	0	0
COL6A5	Collagen type vi alpha 5 chain	3105	271	0.09	Down	0	0
COL6A6	Collagen type vi alpha 6 chain	2167	834	0.38	Down	8.79E-112	4.27E-101
COL7A1	Collagen type vii alpha 1 chain	10545	1125	0.11	Down	0	0
CORO2A	Coronin 2A	522	72	0.14	Down	1.02E-72	3.99E-64
CPA4	Carboxypeptidase A4	4127	771	0.19	Down	0	0
CPD	Carboxypeptidase D	5054	1828	0.36	Down	1.38E-290	1.27E-281
CPEB2	Cytoplasmic polyadenylation element binding protein 2	4934	2289	0.46	Down	3.81E-174	2.45E-164
CPM	Carboxypeptidase M	4135	1793	0.43	Down	1.30E-168	8.21E-160
CPT1A	Carnitine palmitoyltransferase 1A	2202	645	0.29	Down	1.20E-168	7.55E-160
CPT1B	Carnitine palmitoyltransferase 1B	606	87	0.14	Down	4.27E-84	1.79E-74
CRAT	Carnitine O-acetyltransferase	15803	4532	0.29	Down	0	0
CRISP2	Cysteine rich secretory protein 2	559	61	0.11	Down	1.61E-88	6.96E-80
CRISP3	Cysteine rich secretory protein 3	3643	390	0.11	Down	0	0
CRNN	Cornulin	62871	999	0.02	Down	0	0
CROT	Carnitine O-octanoyltransferase	3006	889	0.30	Down	6.55E-230	5.10E-220
CRTAC1	Cartilage acidic protein 1	226	57	0.25	Down	1.17E-16	2.67E-08
CRYBG3	Crystallin beta-gamma domain containing 3	1637	709	0.43	Down	1.92E-64	7.09E-56
CSAD	Cysteine sulfinic acid decarboxylase	3569	1170	0.33	Down	2.78E-238	2.21E-228
CSF3R	Colony stimulating factor 3 receptor	212	48	0.23	Down	1.58E-18	3.64E-10
CSGALNACT1	Chondroitin sulfate N-acetylgalactosaminyltransferase 1	512	132	0.26	Down	2.90E-43	8.96E-34
CSPG4	Chondroitin sulfate proteoglycan 4	7393	1045	0.14	Down	0	0
CTNND2	Catenin delta 2	1666	181	0.11	Down	9.25E-265	8.10E-264
CX43	Gap junction protein alpha 1	48026	7498	0.16	Down	0	0
CYB5A	Cytochrome b5 type A	11129	3355	0.30	Down	0	0
CYFIP2	Cytoplasmic FMR1 interacting protein 2	3150	591	0.19	Down	0	0
CYP19A1	Cytochrome P450 family 19 subfamily A member 1	4719	1502	0.32	Down	0	0
CYP1A1	Cytochrome P450 family 1 subfamily A member 1	160	7	0.04	Down	7.43E-33	2.03E-22
CYP2D50	Cytochrome P450 family 2 subfamily D member 50	195	40	0.21	Down	1.45E-17	3.34E-09
CYP3A93	Cytochrome P450 family 3 subfamily A member 93	1251	286	0.23	Down	9.77E-126	5.11E-115
CYR61	Cellular communication network factor 1	8034	2867	0.36	Down	0	0
DACH1	Dachshund family transcription factor 1	775	95	0.12	Down	1.95E-117	9.88E-109
DBC1	BMP/retinoic acid inducible neural specific 1	254	13	0.05	Down	8.13E-51	2.68E-41
DBI	Diazepam binding inhibitor. acyl-coa binding protein	37565	11324	0.30	Down	0	0
DBT	Dihydrolipoamide branched chain transacylase E2	1660	758	0.46	Down	9.02E-59	3.16E-49
DCBLD1	Discoidin. CUB and LCCL domain containing 1	3316	1180	0.36	Down	2.23E-196	1.56E-185
DCST1	DC-STAMP domain containing 1	181	28	0.15	Down	1.49E-20	3.59E-12
DDC	Dopa decarboxylase	442	119	0.27	Down	4.76E-35	1.35E-25
DEFA35L	Paneth cell-specific alpha-defensin 35L	207	11	0.05	Down	2.39E-40	7.15E-31
DENND2C	DENN domain containing 2C	1705	411	0.24	Down	1.49E-161	9.16E-153
DENND2D	DENN domain containing 2D	3104	819	0.26	Down	1.46E-270	1.27E-261
DENND4A	DENN domain containing 4A	2486	1126	0.45	Down	2.55E-92	1.12E-81
DEPDC1	DEP domain containing 1	391	90	0.23	Down	1.77E-35	5.11E-27
DEPDC1B	DEP domain containing 1B	761	198	0.26	Down	3.08E-65	1.14E-55
DHCR24	24-dehydrocholesterol reductase	61618	22388	0.36	Down	0	0
DHTKD1	Dehydrogenase E1 and transketolase domain containing 1	326	89	0.27	Down	1.61E-23	4.02E-15
DIAPH3	Diaphanous related formin 3	847	237	0.28	Down	4.74E-67	1.78E-57

DIO2	Iodothyronine deiodinase 2	4568	1842	0.40	Down	2.30E-217	1.72E-207
DIO3	Iodothyronine deiodinase 3	229	46	0.20	Down	7.36E-23	1.80E-13
DIP2B	Disco interacting protein 2 homolog B	2715	1112	0.41	Down	3.86E-125	2.02E-114
DLG5	Discs large MAGUK scaffold protein 5	6810	2460	0.36	Down	0	0
DLK2	Delta like non-canonical Notch ligand 2	1093	90	0.08	Down	6.83E-200	4.85E-190
DLL1	Delta like canonical Notch ligand 1	861	123	0.14	Down	3.28E-121	1.68E-111
DLL4	Delta like canonical Notch ligand 4	500	96	0.19	Down	4.87E-56	1.68E-46
DMD	Dystrophin	1048	220	0.21	Down	2.19E-112	1.07E-102
DNAH10	Dynein axonemal heavy chain 10	753	305	0.41	Down	7.34E-33	2.03E-23
DNAH2	Dynein axonemal heavy chain 2	350	122	0.35	Down	2.35E-18	5.41E-09
DNAH6	Dynein axonemal heavy chain 6	285	11	0.04	Down	3.50E-61	1.25E-51
DNMBP	Dynamin binding protein	5606	1890	0.34	Down	0	0
DOCK3	Dedicator of cytokinesis 3	705	123	0.17	Down	1.83E-85	7.78E-77
DOCK9	Dedicator of cytokinesis 9	5559	2289	0.41	Down	1.23E-253	1.02E-244
DOK4	Docking protein 4	487	225	0.46	Down	2.23E-14	4.76E-05
DOT1L	DOT1 like histone lysine methyltransferase	5705	2453	0.43	Down	2.25E-239	1.79E-229
DPEP1	Dipeptidase 1	642	92	0.14	Down	2.19E-89	9.48E-80
DPEP2	Dipeptidase 2	329	121	0.37	Down	2.24E-15	4.88E-07
DPH1	Diphthamide biosynthesis 1	1358	564	0.42	Down	8.09E-60	2.83E-49
DQX1	DEAQ-box RNA dependent atpase 1	739	108	0.15	Down	3.65E-102	1.70E-92
DSC1	Desmocollin 1	39489	9811	0.25	Down	0	0
DSC2	Desmocollin 2	28020	992	0.04	Down	0	0
DSEL	Dermatan sulfate epimerase like	512	200	0.39	Down	5.75E-23	1.41E-13
DSG2	Desmoglein 2	4501	264	0.06	Down	0	0
DSG4	Desmoglein 4	2243	487	0.22	Down	5.40E-238	4.27E-227
DSP	Desmoplakin	258095	31894	0.12	Down	0	0
DTL	Denticleless E3 ubiquitin protein ligase	409	127	0.31	Down	1.49E-25	3.81E-17
DTX2	Deltex E3 ubiquitin ligase 2	8528	2717	0.32	Down	0	0
DUSP10	Dual specificity phosphatase 10	1983	832	0.42	Down	1.54E-84	6.51E-76
DUSP16	Dual specificity phosphatase 16	2752	557	0.20	Down	0	0
DZIP1L	DAZ interacting zinc finger protein 1 like	647	210	0.32	Down	3.28E-41	9.92E-32
E2F2	E2f transcription factor 2	456	80	0.18	Down	1.56E-53	5.30E-46
E2F7	E2f transcription factor 7	229	63	0.28	Down	5.07E-17	1.12E-06
E2F8	E2f transcription factor 8	473	118	0.25	Down	3.32E-41	1.00E-31
ECT2	Epithelial cell transforming 2	1289	522	0.40	Down	1.32E-57	4.60E-49
EDEM1	Er degradation enhancing alpha-mannosidase like protein 1	2995	1115	0.37	Down	1.66E-162	1.02E-153
EDEM3	Er degradation enhancing alpha-mannosidase like protein 3	1412	388	0.27	Down	2.31E-116	1.16E-106
EDN1	Endothelin 1	716	172	0.24	Down	1.18E-65	4.38E-57
EDN2	Endothelin 2	398	25	0.06	Down	2.91E-77	1.17E-67
EDN3	Endothelin 3	154	28	0.18	Down	1.29E-14	2.82E-06
EFNB2	Ephrin B2	2836	365	0.13	Down	0	0
EGFR	Epidermal growth factor receptor	4218	639	0.15	Down	0	0
EGR1	Early growth response 1	33274	9333	0.28	Down	0	0
EGR2	Early growth response 2	6227	1825	0.29	Down	0	0
EGR3	Early growth response 3	8415	1097	0.13	Down	0	0
EHHADH	Enoyl-coa hydratase and 3-hydroxyacyl coa	2679	644	0.24	Down	7.58E-258	6.34E-248
EIF2C4	Argonaute RISC component 4	2481	672	0.27	Down	3.25E-210	2.38E-200

ELL2	Elongation factor for RNA polymerase II 2	4141	1096	0.26	Down	0	0
ELL3	Elongation factor for RNA polymerase II 3	318	110	0.35	Down	1.36E-16	3.05E-07
ELTD1	Adhesion G protein-coupled receptor L4	479	133	0.28	Down	7.68E-38	2.22E-27
EML1	Echinoderm microtubule associated protein like 1	1154	474	0.41	Down	1.19E-49	3.93E-41
ENC1	Ectodermal-neural cortex 1	1113	518	0.47	Down	1.59E-35	4.59E-27
ENDOD1	Endonuclease domain containing 1	2093	314	0.15	Down	1.74E-289	1.59E-281
ENDOU	Endonuclease. poly(U) specific	3474	766	0.22	Down	0	0
ENPP5	Ectonucleotide pyrophosphatase/phosphodiesterase family member 5	2615	792	0.30	Down	1.25E-192	8.65E-184
ENTPD1	Ectonucleoside triphosphate diphosphohydrolase 1	670	312	0.47	Down	1.28E-19	3.04E-12
EPAS1	Endothelial PAS domain protein 1	5065	1305	0.26	Down	0	0
EPB41L1	Erythrocyte membrane protein band 4.1 like 1	3042	732	0.24	Down	6.82E-293	6.25E-283
EPG5	Ectopic P-granules 5 autophagy tethering factor	1732	803	0.46	Down	3.28E-59	1.15E-50
EPHA2	Ephrin type-A receptor 2	5948	1724	0.29	Down	0	0
EPHA4	Ephrin type-A receptor 4	4529	1153	0.25	Down	0	0
EPHA7	Ephrin type-A receptor 7	395	65	0.16	Down	5.00E-49	1.63E-39
EPHB1	Ephrin type-B receptor 1	1122	95	0.08	Down	3.33E-203	2.39E-193
EPHB2	Ephrin type-B receptor 2	1193	309	0.26	Down	1.32E-103	6.20E-96
EPHB3	Ephrin type-B receptor 3	13897	4693	0.34	Down	0	0
EPHB6	Ephrin type-B receptor 6	17381	3518	0.20	Down	0	0
EPM2AIP1	EPM2A interacting protein 1	569	248	0.44	Down	2.89E-20	6.84E-11
EPN2	Epsin 2	2659	1046	0.39	Down	2.81E-131	1.52E-121
EPN3	Epsin 3	1915	347	0.18	Down	8.59E-234	6.74E-224
ERBB2	Erb-b2 receptor tyrosine kinase 2	14228	3919	0.28	Down	0	0
ERBB4	Erb-b2 receptor tyrosine kinase 4	166	2	0.01	Down	1.14E-40	3.37E-30
ERG	ETS transcription factor	652	259	0.40	Down	3.65E-29	9.67E-20
ERMP1	Endoplasmic reticulum metallopeptidase 1	1740	461	0.26	Down	3.24E-150	1.89E-140
ERN1	Endoplasmic reticulum to nucleus signaling 1	832	278	0.33	Down	1.26E-50	4.19E-42
ERRFI1	ERBB receptor feedback inhibitor 1	3493	1342	0.38	Down	5.44E-181	3.58E-171
ESPL1	Extra spindle pole bodies like 1. separase	962	142	0.15	Down	3.73E-133	2.03E-123
ESRP1	Epithelial splicing regulatory protein 1	22997	3254	0.14	Down	0	0
ESYT2	Extended synaptotagmin-like protein 2	4389	1781	0.41	Down	3.17E-207	2.30E-196
ESYT3	Extended synaptotagmin-like protein 3	1392	373	0.27	Down	4.88E-118	2.46E-108
EVPL	Envoplakin	29364	4391	0.15	Down	0	0
EXO1	Exonuclease 1	333	33	0.10	Down	2.59E-56	8.78E-45
EXOC6B	Exocyst complex component 6B	1665	701	0.42	Down	8.42E-73	3.24E-61
EXPH5	Exophilin 5	3027	482	0.16	Down	0	0
EYA2	EYA transcriptional coactivator and phosphatase 2	624	108	0.17	Down	1.34E-75	5.35E-67
EZH2	Enhancer of zeste 2 polycomb repressive complex 2	2628	1163	0.44	Down	5.35E-103	2.50E-92
EZR	Ezrin	42771	10678	0.25	Down	0	0
F13A1	Coagulation factor XIII. A1 subunit	4248	1179	0.28	Down	0	0
FAAH	Fatty acid amide hydrolase	963	169	0.18	Down	8.77E-119	4.45E-109
FABP3	Fatty acid binding protein 3	1713	350	0.20	Down	5.47E-190	3.74E-180
FANCD2	Fanconi anemia. complementation group D2	1886	339	0.18	Down	1.41E-231	1.10E-222
FANCM	Fanconi anemia. complementation group M	557	259	0.46	Down	1.23E-15	2.76E-07
FAR2	Fatty acyl coa reductase 2	6186	404	0.07	Down	0	0
FASN	Fatty acid synthase	92350	24024	0.26	Down	0	0
FAT1	FAT atypical cadherin 1	14694	6698	0.46	Down	0	0
FBN2	Fibrillin 2	566	157	0.28	Down	3.42E-44	1.06E-34

FBXL18	F-box and leucine rich repeat protein 18	893	392	0.44	Down	3.66E-33	1.02E-23
FBXL19	F-box and leucine rich repeat protein 19	1591	739	0.46	Down	7.06E-54	2.39E-44
FBXL20	F-box and leucine rich repeat protein 20	923	398	0.43	Down	7.68E-36	2.20E-26
FBXL6	F-box and leucine rich repeat protein 6	2166	928	0.43	Down	1.10E-88	4.75E-84
FBXO42	F-box protein 42	2383	1019	0.43	Down	2.16E-100	9.93E-90
FCER1A	Fc epsilon receptor Ia	1497	454	0.30	Down	2.41E-109	1.16E-99
FCER2	Fc epsilon receptor II	872	218	0.25	Down	1.46E-77	5.92E-69
FDXR	Ferredoxin reductase	1842	519	0.28	Down	2.68E-148	1.55E-138
FERMT1	FERM domain containing kindlin 1	1289	163	0.13	Down	6.41E-196	4.50E-186
FGD6	FYVE. rhogef and PH domain containing 6	727	248	0.34	Down	2.91E-43	8.98E-34
FGFR2	Fibroblast growth factor receptor 2	11793	1670	0.14	Down	0	0
FGFR3	Fibroblast growth factor receptor 3	6371	1009	0.16	Down	0	0
FHDC1	FH2 domain containing 1	2234	356	0.16	Down	1.00E-289	9.26E-289
FHOD3	Formin homology 2 domain containing 3	2566	756	0.29	Down	9.32E-188	6.57E-187
FILIP1L	Filamin A interacting protein 1 like	10218	2478	0.24	Down	0	0
FLNB	Filamin B	23912	8968	0.38	Down	0	0
FLRT2	Fibronectin leucine rich transmembrane protein 2	1273	497	0.39	Down	4.25E-62	1.53E-52
FLT1	FMS-like tyrosine kinase 1	544	152	0.28	Down	5.78E-43	1.76E-32
FMO5	Flavin containing dimethylaniline monoxygenase 5	2047	694	0.34	Down	7.94E-129	4.24E-119
FN1	Fibronectin 1	92625	37888	0.41	Down	0	0
FNDC1	Fibronectin type III domain containing 1	34506	8273	0.24	Down	0	0
FOSL2	FOS like 2. AP-1 transcription factor subunit	5334	1269	0.24	Down	0	0
FOXC2	Forkhead box C2	144	18	0.13	Down	5.98E-19	1.39E-09
FOXJ1	Forkhead box J1	152	10	0.07	Down	6.93E-28	1.79E-17
FOXM1	Forkhead box M1	690	174	0.25	Down	6.46E-61	2.30E-51
FOXN1	Forkhead box N1	2523	768	0.30	Down	1.72E-184	1.16E-175
FRAS1	Fraser extracellular matrix complex subunit 1	362	39	0.11	Down	3.98E-57	1.38E-47
FREM1	FRAS1 related extracellular matrix 1	461	203	0.44	Down	2.38E-15	5.18E-06
FRMD6	FERM domain containing 6	3732	1484	0.40	Down	8.59E-182	5.68E-172
FRMD7	FERM domain containing 7	161	31	0.19	Down	1.19E-14	2.61E-06
FRMPD1	FERM and PDZ domain containing 1	1178	290	0.25	Down	5.54E-109	2.67E-99
FRRS1	Ferric chelate reductase 1	1858	169	0.09	Down	0	0
FRS2	Fibroblast growth factor receptor substrate 2	959	345	0.36	Down	2.74E-53	9.20E-44
FRY	FRY microtubule binding protein	3959	1539	0.39	Down	3.00E-201	2.14E-191
FSD2	Fibronectin type III and SPRY domain containing 2	1386	269	0.19	Down	1.06E-158	6.38E-151
FST	Follistatin	2663	494	0.19	Down	0	0
FZD4	Frizzled class receptor 4	623	212	0.34	Down	9.56E-37	2.76E-27
GABRP	Gamma-aminobutyric acid type A receptor subunit pi	8128	1063	0.13	Down	0	0
GALNT4	Polypeptide N-acetylgalactosaminyltransferase 4	355	131	0.37	Down	9.39E-17	2.11E-07
GALNT5	Polypeptide N-acetylgalactosaminyltransferase 5	115	13	0.11	Down	2.69E-15	5.86E-06
GALNT6	Polypeptide N-acetylgalactosaminyltransferase 6	2431	900	0.37	Down	2.08E-133	1.14E-123
GAN	Gigaxonin	808	258	0.32	Down	1.50E-52	5.06E-44
GATA2	GATA binding protein 2	591	114	0.19	Down	1.37E-65	5.11E-57
GCAT	Glycine C-acetyltransferase	1029	328	0.32	Down	2.98E-69	1.14E-60
GCNT2	N-acetyllactosaminide beta-1.6-N-acetylglucosaminyl-transferase	240	70	0.29	Down	1.33E-14	2.90E-06
GDPD2	Glycerophosphodiester phosphodiesterase domain containing 2	3018	512	0.17	Down	0	0
GGT1	Gamma-glutamyltransferase 1	509	54	0.11	Down	2.76E-83	1.15E-72

GGT6	Gamma-glutamyltransferase 6	9156	748	0.08	Down	0	0
GJD3	Gap junction protein delta 3	299	81	0.27	Down	1.99E-21	4.84E-13
GLB1L2	Galactosidase beta 1 like 2	986	157	0.16	Down	3.79E-131	2.04E-120
GLI1	GLI family zinc finger 1	2334	388	0.17	Down	0	0
GLI3	GLI family zinc finger 3	1836	674	0.37	Down	1.89E-100	8.76E-93
GLUL	Glutamate-ammonia ligase	19142	6707	0.35	Down	0	0
GPAM	Glycerol-3-phosphate acyltransferase. mitochondrial	11406	1100	0.10	Down	0	0
GPC4	Glypican 4	3848	814	0.21	Down	0	0
GPD1	Glycerol-3-phosphate dehydrogenase 1	1680	470	0.28	Down	3.89E-136	2.15E-126
GPLD1	Glycosylphosphatidylinositol specific phospholipase D1	1498	648	0.43	Down	8.89E-60	3.14E-50
GPR111	G protein-coupled receptor 111	504	141	0.28	Down	1.42E-38	4.18E-30
GPR115	G protein-coupled receptor 115	6349	1081	0.17	Down	0	0
GPR116	G protein-coupled receptor 116	638	132	0.21	Down	5.11E-69	1.93E-58
GPR124	G protein-coupled receptor 124	5550	2551	0.46	Down	1.24E-199	8.82E-192
GPR126	G protein-coupled receptor 126	4284	261	0.06	Down	0	0
GPR183	G protein-coupled receptor 183	828	377	0.46	Down	7.12E-28	1.86E-18
GPR39	G protein-coupled receptor 39	94	5	0.05	Down	2.79E-16	6.18E-07
GPR87	G protein-coupled receptor 87	2983	567	0.19	Down	0	0
GPR98	G protein-coupled receptor 98	2736	453	0.17	Down	0	0
GPRC5C	G protein-coupled receptor class C group 5 member C	1939	232	0.12	Down	0	0
GPSM2	G protein signaling modulator 2	12587	3924	0.31	Down	0	0
GPT2	Glutamic--pyruvic transaminase 2	5393	1015	0.19	Down	0	0
GPX2	Glutathione peroxidase 2	7247	1059	0.15	Down	0	0
GPX3	Glutathione peroxidase 3	109181	18553	0.17	Down	0	0
GRAMD1C	GRAM domain containing 1C	838	233	0.28	Down	8.15E-67	3.04E-57
GRAMD3	GRAM domain containing 3	5778	1212	0.21	Down	0	0
GRB14	Growth factor receptor bound protein 14	1958	442	0.23	Down	2.55E-200	1.81E-189
GRB7	Growth factor receptor bound protein 7	3391	567	0.17	Down	0	0
GREB1	Growth regulating estrogen receptor binding 1	154	28	0.18	Down	1.29E-14	2.82E-06
GREB1L	GREB1 like retinoic acid receptor coactivator	419	114	0.27	Down	1.26E-31	3.48E-23
GRHL1	Grainyhead like transcription factor 1	11487	1529	0.13	Down	0	0
GRHL2	Grainyhead like transcription factor 2	2750	291	0.11	Down	0	0
GRHL3	Grainyhead like transcription factor 3	3530	612	0.17	Down	0	0
GRIN1	Glutamate ionotropic receptor NMDA type subunit 1	89	6	0.07	Down	4.53E-14	9.62E-05
GRIP1	Glutamate receptor interacting protein 1	1158	129	0.11	Down	3.89E-188	2.63E-177
GSDMA	Gasdermin A	9137	1680	0.18	Down	0	0
GTSE1	G2 and S-phase expressed 1	1407	311	0.22	Down	3.71E-145	2.12E-135
GXYLT2	Glucoside xylosyltransferase 2	357	124	0.35	Down	6.54E-19	1.52E-09
GYLTL1B	LARGE xylosyl- and glucuronyltransferase 2	7658	666	0.09	Down	0	0
GYS2	Glycogen synthase 2	896	77	0.09	Down	9.95E-161	6.03E-151
H19	H19. imprinted maternally expressed transcript (non-protein coding)	9211	1531	0.17	Down	0	0
HAPLN1	Hyaluronan and proteoglycan link protein 1	108	2	0.02	Down	1.92E-22	4.73E-14
HAS2	Hyaluronan synthase 2	718	317	0.44	Down	1.20E-24	3.06E-16
HAUS7	HAUS augmin like complex subunit 7	262	85	0.32	Down	1.16E-13	2.48E-05
HELB	DNA helicase B	476	219	0.46	Down	3.81E-15	8.10E-05
HEPHL1	Hephaestin like 1	3777	512	0.14	Down	0	0
HERC6	HECT and RLD domain containing E3 ubiquitin protein ligase family member 6	1687	654	0.39	Down	2.01E-85	8.45E-75

HHIP	Hedgehog interacting protein	115	2	0.02	Down	2.15E-25	5.46E-16
HIP1R	Huntingtin interacting protein 1 related	10401	3067	0.29	Down	0	0
HJURP	Holliday junction recognition protein	677	111	0.16	Down	1.27E-85	5.44E-77
HK2	Hexokinase 2	2038	888	0.44	Down	3.60E-81	1.49E-71
HLCS	Holocarboxylase synthetase	1726	787	0.46	Down	1.90E-60	6.79E-53
HMCN1	Hemicentin 1	5171	1340	0.26	Down	0	0
HMGCR	3-hydroxy-3-methylglutaryl-coa reductase	10720	3798	0.35	Down	0	0
HMGCS1	3-hydroxy-3-methylglutaryl-coa synthase 1	33954	7561	0.22	Down	0	0
HOMER2	Homer scaffold protein 2	5502	855	0.16	Down	0	0
HOOK1	Hook microtubule tethering protein 1	2302	344	0.15	Down	0	0
HOOK2	Hook microtubule tethering protein 2	3121	645	0.21	Down	0	0
HPGD	15-hydroxyprostaglandin dehydrogenase	1632	446	0.27	Down	7.75E-138	4.28E-126
HR	HR lysine demethylase and nuclear receptor corepressor	24921	3328	0.13	Down	0	0
HS6ST2	Heparan sulfate 6-O-sulfotransferase 2	352	88	0.25	Down	9.96E-30	2.65E-20
HSD11B2	Hydroxysteroid 11-beta dehydrogenase 2	1966	126	0.06	Down	0	0
HSPA1A	Heat shock protein family A (Hsp70) member 1B	5325	1532	0.29	Down	0	0
HSPG2	Heparan sulfate proteoglycan 2	14012	4110	0.29	Down	0	0
HTR3A	5-hydroxytryptamine receptor 3A	149	17	0.11	Down	8.64E-21	2.06E-11
IDE	Insulin degrading enzyme	6656	2664	0.40	Down	0	0
IDH1	Isocitrate dehydrogenase (NADP(+)) 1	9619	4040	0.42	Down	0	0
IGF1R	Insulin like growth factor 1 receptor	2113	863	0.41	Down	1.81E-95	8.17E-87
IGFBP3	Insulin like growth factor binding protein 3	5830	1198	0.21	Down	0	0
IGFBP2	Insulin like growth factor binding protein 2	3926	1529	0.39	Down	7.04E-199	4.98E-189
IGHC1	Immunogobulin gamma 1 heavy chain constant region	23223	609	0.03	Down	0	0
IGSF3	Immunoglobulin superfamily member 3	4505	1442	0.32	Down	0	0
IL-1RII	Interleukin 1 receptor type 2	14874	1663	0.11	Down	0	0
IL17RC	Interleukin 17 receptor c	4577	1153	0.25	Down	0	0
IL1R1	Interleukin 1 receptor type 1	2208	609	0.28	Down	6.69E-185	4.45E-173
IL1RAP	Interleukin 1 receptor accessory protein	866	350	0.40	Down	1.84E-37	5.39E-29
IL1RN	Interleukin 1 receptor antagonist	2851	515	0.18	Down	0	0
IL22RA1	Interleukin 22 receptor subunit alpha 1	1315	307	0.23	Down	1.15E-127	6.14E-119
IL31RA	Interleukin 31 receptor a	213	24	0.11	Down	1.85E-30	5.04E-22
INADL	Patj crumbs cell polarity complex component	3710	1079	0.29	Down	2.05E-290	1.87E-280
INCENP	Inner centromere protein	2513	1142	0.45	Down	1.25E-90	5.46E-82
INHA	Inhibin subunit alpha	207	33	0.16	Down	1.28E-23	3.21E-16
INPP1	Inositol polyphosphate-1-phosphatase	2063	732	0.35	Down	5.73E-121	2.94E-111
INPP4B	Inositol polyphosphate-4-phosphatase type ii b	351	66	0.19	Down	5.28E-39	1.56E-29
INPP5J	Inositol polyphosphate-5-phosphatase j	187	26	0.14	Down	4.57E-24	1.14E-14
INSR	Insulin receptor	1454	568	0.39	Down	2.65E-71	1.03E-61
IPMK	Inositol polyphosphate multikinase	314	100	0.32	Down	7.97E-19	1.85E-09
IQGAP3	IQ Motif Containing gtpase Activating Protein 3	788	148	0.19	Down	8.62E-92	3.79E-83
IRF6	Interferon regulatory factor 6	16700	2110	0.13	Down	0	0
IRX4	Iroquois homeobox 4	1936	224	0.12	Down	0	0
ITGA3	Integrin subunit alpha 3	6022	2618	0.43	Down	5.47E-247	4.45E-237
ITGA6	Integrin subunit alpha 6	9658	1874	0.19	Down	0	0
ITGA7	Integrin subunit alpha 7	2887	389	0.13	Down	0	0
ITGAV	Integrin subunit alpha v	3746	1488	0.40	Down	7.60E-183	5.04E-173
ITGB4	Integrin subunit beta 4	38678	12985	0.34	Down	0	0

ITGB6	Integrin subunit beta 6	820	73	0.09	Down	7.47E-145	4.26E-135
ITGB7	Integrin subunit beta 7	1997	500	0.25	Down	1.11E-182	7.40E-174
ITIH4	Inter-alpha-trypsin inhibitor heavy chain 4	1043	152	0.15	Down	9.22E-146	5.27E-136
ITPKC	Inositol-trisphosphate 3-kinase c	2362	1067	0.45	Down	3.78E-88	1.62E-77
ITPR2	Inositol 1.4.5-trisphosphate receptor type 2	6552	1495	0.23	Down	0	0
ITPR3	Inositol 1.4.5-trisphosphate receptor type 3	11299	5040	0.45	Down	0	0
JAG1	Jagged canonical notch ligand 1	12893	3438	0.27	Down	0	0
JHDM1D	Lysine demethylase 7a	791	284	0.36	Down	1.58E-43	4.88E-34
KAL1	Aka anos1 anosmin 1	531	62	0.12	Down	3.96E-83	1.64E-72
KALRN	Kalirin rhogef Kinase	488	65	0.13	Down	4.24E-70	1.62E-60
KANK1	Kn motif and ankyrin repeat domains 1	10238	1834	0.18	Down	0	0
KCNC4	Potassium voltage-gated channel subfamily c member 4	274	77	0.28	Down	2.71E-19	6.33E-11
KCNJ15	Potassium inwardly rectifying channel subfamily j member 15	1422	147	0.10	Down	1.84E-238	1.46E-228
KCNK2	Potassium two pore domain channel subfamily k member 2	534	222	0.42	Down	4.60E-21	1.10E-11
KCNK5	Potassium two pore domain channel subfamily k member 5	682	67	0.10	Down	3.27E-115	1.63E-105
KCNU1	Potassium calcium-activated channel subfamily u member 1	262	28	0.11	Down	1.52E-39	4.55E-39
KDM1B	Lysine demethylase 1b	1139	398	0.35	Down	5.02E-67	1.88E-57
KDM2B	Lysine demethylase 2b	6135	2585	0.42	Down	1.15E-267	9.91E-259
KDM5B	Lysine demethylase 5b	12527	4739	0.38	Down	0	0
KIF11	Kinesin superfamily protein 11	1691	320	0.19	Down	1.95E-198	1.39E-189
KIF12	Kinesin superfamily protein	369	15	0.04	Down	1.90E-79	7.75E-70
KIF13A	Kinesin superfamily protein	5035	2093	0.42	Down	4.57E-226	3.52E-216
KIF13B	Kinesin superfamily protein	2369	745	0.31	Down	1.41E-165	8.78E-157
KIF15	Kinesin superfamily protein	1188	291	0.24	Down	1.67E-109	8.11E-102
KIF18B	Kinesin superfamily protein	521	139	0.27	Down	2.28E-42	6.98E-33
KIF1B	Kinesin superfamily protein	5271	2265	0.43	Down	3.15E-221	2.38E-211
KIF20A	Kinesin superfamily protein	1612	342	0.21	Down	5.90E-173	3.77E-163
KIF21A	Kinesin superfamily protein	7856	2017	0.26	Down	0	0
KIF23	Kinesin superfamily protein	1145	282	0.25	Down	8.57E-106	4.06E-96
KIF26A	Kinesin superfamily protein	2103	371	0.18	Down	4.23E-263	3.59E-252
KIF2C	Kinesin superfamily protein	1042	182	0.17	Down	3.14E-129	1.68E-119
KIF4A	Kinesin superfamily protein	856	163	0.19	Down	6.47E-99	2.96E-89
KIFC1	Kinesin superfamily protein	1522	505	0.33	Down	4.09E-98	1.86E-88
KITLG	Kit ligand	441	117	0.27	Down	1.38E-35	3.95E-26
KLF10	Klf transcription factor 10	7937	2871	0.36	Down	0	0
KLF4	Klf transcription factor 4	20447	3992	0.20	Down	0	0
KLF5	Klf transcription factor 5	14558	2394	0.16	Down	0	0
KLHDC7A	Kelch domain containing 7a	444	31	0.07	Down	7.69E-85	3.22E-74
KLHDC8A	Kelch domain containing 8a	337	41	0.12	Down	8.36E-51	2.73E-40
KLK1E1	Glandular kallikrein 1. horse	530	215	0.41	Down	4.66E-23	1.13E-12
KNTC1	Kinetochore associated 1	922	369	0.40	Down	5.50E-42	1.68E-32
KREMEN1	Kringle containing transmembrane protein 1	3186	1030	0.32	Down	1.24E-215	9.28E-207
KRT23	Keratin 23	10465	2133	0.20	Down	0	0
KRT25	Keratin 25	44412	6805	0.15	Down	0	0
KRT26	Keratin 26	4550	915	0.20	Down	0	0
KRT28	Keratin 28	7524	694	0.09	Down	0	0

KRT36	Keratin 36	480	212	0.44	Down	5.35E-16	1.18E-06
KRT37	Keratin 37	102	3	0.03	Down	1.54E-20	3.65E-11
KRT4	Keratin 4	105756	1184	0.01	Down	0	0
KRT40	Keratin 40	2454	559	0.23	Down	1.77E-247	1.45E-239
KRT5	Keratin 5	544061	50334	0.09	Down	0	0
KRT7	Keratin 7	7312	915	0.13	Down	0	0
KRT71	Keratin 71	39378	7015	0.18	Down	0	0
KRT73	Keratin 73	6430	742	0.12	Down	0	0
KRT77	Keratin 77	34998	8203	0.23	Down	0	0
KRT79	Keratin 79	114073	5958	0.05	Down	0	0
KRT80	Keratin 80	9817	2083	0.21	Down	0	0
KRT82	Keratin 82	2637	506	0.19	Down	0	0
KSR1	Kinase suppressor of ras 1	1401	591	0.42	Down	7.96E-59	2.79E-50
LAMA3	Laminin subunit alpha 3	8883	1566	0.18	Down	0	0
LAMA5	Laminin subunit alpha 5	17230	2458	0.14	Down	0	0
LAMB4	Laminin subunit beta 4	1582	197	0.12	Down	4.20E-243	3.38E-233
LAMC2	Laminin subunit gamma 2	4823	546	0.11	Down	0	0
LARGE	Large xylosyl- and glucuronyltransferase	1119	472	0.42	Down	2.42E-47	7.69E-37
LARP1B	La ribonucleoprotein 1b	1243	393	0.32	Down	2.75E-86	1.16E-75
LASS6	Ceramide synthase 6	845	305	0.36	Down	2.65E-46	8.42E-38
LATH	Bpi fold containing family a member 4. pseudogene	1109772	81634	0.07	Down	0	0
LBR	Lamin b receptor	1536	613	0.40	Down	1.71E-71	6.66E-63
LCLAT1	Lysocardiolipin acyltransferase 1	408	149	0.37	Down	3.61E-20	8.54E-11
LEPREL1	Prolyl 3-hydroxylase 2	3733	482	0.13	Down	0	0
LGI2	Leucine rich repeat lgi family member 2	409	143	0.35	Down	6.55E-23	1.58E-12
LGR4	Leucine-rich repeat-containing G-protein coupled receptor 4	7301	1695	0.23	Down	0	0
LGR5	Leucine-rich repeat-containing G-protein coupled receptor 5	805	229	0.28	Down	2.77E-62	9.97E-53
LGR6	Leucine-rich repeat-containing G-protein coupled receptor 6	957	60	0.06	Down	1.19E-189	8.18E-181
LHB	Luteinizing hormone subunit beta	225	42	0.19	Down	6.52E-24	1.62E-14
LIG4	Dna ligase 4	1345	524	0.39	Down	4.71E-67	1.75E-56
LIMK2	Lim domain kinase 2	16490	3107	0.19	Down	0	0
LINGO1	Leucine rich repeat and ig domain containing 1	1551	229	0.15	Down	1.42E-217	1.06E-206
LIPG	Lipase g. endothelial type	457	53	0.12	Down	1.87E-69	7.19E-61
LIPH	Lipase H	490	106	0.22	Down	1.97E-48	6.41E-40
LIPK	Lipase family member k	1641	400	0.24	Down	1.36E-154	8.07E-145
LIPM	Lipase family member m	1066	341	0.32	Down	1.70E-70	6.60E-62
LIPN	Lipase family member n	1787	714	0.40	Down	1.15E-83	4.82E-75
LLGL1	Llgl scribble cell polarity complex component 1	7523	2624	0.35	Down	0	0
LMBR1	Limb development membrane protein 1	3044	931	0.31	Down	5.05E-223	3.84E-213
LMBRD2	Lmbr1 domain containing 2	1065	429	0.40	Down	2.24E-48	7.24E-39
LMNB1	Lamin B1	2624	978	0.37	Down	1.34E-141	7.60E-133
LMO7	LIM domain 7	5268	813	0.15	Down	0	0
LMOD1	Leiomodin 1	2302	959	0.42	Down	2.88E-102	1.34E-91
LMTK2	Lemur tyrosine kinase 2	4217	1082	0.26	Down	0	0
LNPEP	Leucyl and cystinyl aminopeptidase	523	200	0.38	Down	1.46E-24	3.65E-15
LNX2	Ligand of numb-protein X 2	901	311	0.35	Down	2.16E-53	7.27E-44
LONRF1	LON peptidase N-terminal domain and ring finger 1	4538	1697	0.37	Down	2.18E-249	1.78E-237

LPCAT3	Lysophosphatidylcholine acyltransferase 3	16137	3639	0.23	Down	0	0
LPGAT1	Lysophosphatidylglycerol acyltransferase 1	8586	857	0.10	Down	0	0
LPHN2	Adhesion G protein-coupled receptor L2	9094	3218	0.35	Down	0	0
LPIN1	Lipin 1	1037	440	0.42	Down	3.68E-43	1.12E-32
LPIN3	Lipin 3	2473	558	0.23	Down	1.20E-251	9.93E-251
LPO	Lactoperoxidase	5994	653	0.11	Down	0	0
LRIG1	Leucine rich repeats and immunoglobulin like domains 1	8171	2459	0.30	Down	0	0
LRIG3	Leucine rich repeats and immunoglobulin like domains 3	8134	2385	0.29	Down	0	0
LRP4	LDL receptor related protein 4	3059	826	0.27	Down	8.64E-261	7.29E-251
LRP5	LDL receptor related protein 5	5344	1809	0.34	Down	0	0
LRRC1	Leucine rich repeat containing 1	3077	774	0.25	Down	2.88E-283	2.58E-273
LRRC15	Leucine rich repeat containing 15	4309	1839	0.43	Down	6.36E-183	4.23E-173
LRRC17	Leucine rich repeat containing 17	1965	722	0.37	Down	1.24E-107	5.95E-99
LRRC8A	Leucine rich repeat containing 8A	4289	1934	0.45	Down	2.05E-161	1.24E-151
LRRC8B	Leucine rich repeat containing 8B	1761	503	0.29	Down	2.50E-140	1.40E-129
LRRC8E	Leucine rich repeat containing 8E	1334	186	0.14	Down	3.03E-192	2.10E-182
LSR	Lipolysis stimulated lipoprotein receptor	3690	510	0.14	Down	0	0
LSS	Lanosterol synthase	5104	1560	0.31	Down	0	0
LTBP1	Latent transforming growth factor beta binding protein 1	3686	1556	0.42	Down	1.25E-159	7.55E-150
LTF	Lactotransferrin	1996	313	0.16	Down	2.67E-269	2.31E-259
LYG2	Lysozyme g-like protein 2	667	138	0.21	Down	2.94E-71	1.13E-61
LZTS1	Leucine zipper tumor suppressor 1	540	148	0.27	Down	1.23E-41	3.79E-33
MAGI3	Membrane associated guanylate kinase. WW and PDZ domain containing 3	1498	459	0.31	Down	6.13E-108	2.93E-99
MAML3	Mastermind like transcriptional coactivator 3	1331	416	0.31	Down	5.08E-93	2.24E-84
MAN2A1	Mannosidase alpha class 2A member 1	1401	437	0.31	Down	2.39E-99	1.09E-88
MAP2	Microtubule associated protein 2	2426	503	0.21	Down	5.04E-268	4.33E-257
MAP3K1	Mitogen-activated protein kinase 1	3204	1098	0.34	Down	4.39E-200	3.12E-191
MAP3K8	Mitogen-activated protein kinase 8	1032	362	0.35	Down	4.73E-60	1.67E-51
MAP3K9	Mitogen-activated protein kinase 9	547	143	0.26	Down	1.02E-44	3.23E-36
MAP6	Microtubule associated protein 6	1347	410	0.30	Down	1.33E-96	6.02E-88
MAP7	Microtubule associated protein 7	3790	807	0.21	Down	0	0
MAPKBP1	Mitogen-activated protein kinase binding protein 1	2886	1302	0.45	Down	1.69E-107	8.08E-98
MARK4	Microtubule affinity regulating kinase 4	3046	1343	0.44	Down	1.08E-118	5.48E-110
MARVELD2	MARVEL domain containing 2	976	119	0.12	Down	2.11E-150	1.23E-140
MASP1	MBL associated serine protease 1	435	183	0.42	Down	4.67E-16	1.03E-06
MAST4	Microtubule associated serine/threonine kinase family member 4	5186	1066	0.21	Down	0	0
MASTL	Microtubule associated serine/threonine kinase like	453	126	0.28	Down	1.39E-33	3.92E-25
MATN4	Matrilin 4	418	67	0.16	Down	4.88E-53	1.64E-43
MBTD1	Mbt domain containing 1	663	259	0.39	Down	9.87E-31	2.67E-22
MCAM	Melanoma cell adhesion molecule; cd146; muc18	1812	323	0.18	Down	1.00E-222	7.65E-214
MCM10	Minichromosome maintenance 10 replication initiation factor	284	42	0.15	Down	6.66E-37	1.93E-27
MCM5	Minichromosome maintenance 5 replication initiation factor	5979	2759	0.46	Down	2.27E-214	1.69E-204
MCM9	Minichromosome maintenance 9 replication initiation factor	847	380	0.45	Down	1.14E-28	3.02E-20
MCTP2	Multiple C2 and transmembrane domain containing 2	846	261	0.31	Down	5.15E-61	1.81E-49
ME1	Malic enzyme 1	16806	4044	0.24	Down	0	0

MECOM	MDS1 and EVI1 complex locus	793	121	0.15	Down	5.86E-108	2.79E-97
MEGF6	Multiple EGF like domains 6	3950	730	0.18	Down	0	0
MEIS2	Meis homeobox 2	596	204	0.34	Down	1.12E-33	3.16E-25
MICAL2	Microtubule associated monooxygenase. calponin and LIM domain containing 2	2151	620	0.29	Down	5.56E-169	3.50E-159
MICALCL	Microtubule associated monooxygenase. calponin and LIM domain containing 2	591	151	0.26	Down	5.81E-52	1.92E-41
MICALL1	MICAL like 1	10070	3308	0.33	Down	0	0
MID2	Midline 2	2846	1306	0.46	Down	8.31E-102	3.87E-93
MINK1	Misshapen like kinase 1	25039	9667	0.39	Down	0	0
MKI67	Marker of proliferation Ki-67	4744	756	0.16	Down	0	0
MKL2	Myocardin related transcription factor B	1787	548	0.31	Down	3.20E-129	1.71E-119
MLN	Motilin	106	0	0.00	Down	4.94E-26	1.26E-16
MLXIPL	MLX interacting protein like	327	39	0.12	Down	9.84E-49	3.19E-39
MMEL1	Membrane metalloendopeptidase like 1	211	29	0.14	Down	8.24E-30	2.15E-18
MMP13	Matrix metalloproteinase 13	231	0	0.00	Down	8.65E-62	3.11E-52
MMP28	Matrix metalloproteinase 28	924	157	0.17	Down	1.69E-115	8.42E-107
MMRN1	Multimerin 1	2536	1073	0.42	Down	2.33E-108	1.12E-98
MPP2	MAGUK p55 scaffold protein 2	1355	438	0.32	Down	2.20E-90	9.58E-81
MPP7	MAGUK p55 scaffold protein 7	2886	539	0.19	Down	0	0
MRGPRF	MAS related GPR family member F	4725	1655	0.35	Down	1.07E-285	9.72E-278
MRVI1	Inositol 1.4.5-triphosphate receptor associated 1	428	52	0.12	Down	3.22E-65	1.17E-54
MST1R	Macrophage stimulating 1 receptor	5733	1054	0.18	Down	0	0
MST4	Serine/threonine kinase 26	1178	381	0.32	Down	4.23E-78	1.71E-68
MTA3	Metastasis associated 1 family member 3	7563	1955	0.26	Down	0	0
MTMR10	Myotubularin related protein 10	949	355	0.37	Down	3.15E-49	1.03E-39
MTMR4	Myotubularin related protein 4	1255	560	0.45	Down	3.82E-46	1.21E-36
MTMR7	Myotubularin related protein 7	136	21	0.15	Down	2.52E-15	5.47E-06
MTSS1	MTSS I-BAR domain containing 1	5895	1610	0.27	Down	0	0
MVD	Mevalonate diphosphate decarboxylase	4150	1568	0.38	Down	5.24E-222	3.97E-212
MXRA5	Matrix remodeling associated 5	34084	9953	0.29	Down	0	0
MYB	MYB proto-oncogene. transcription factor	447	48	0.11	Down	1.89E-70	7.33E-62
MYBL2	MYB proto-oncogene like 2	3129	502	0.16	Down	0	0
MYH11	Myosin heavy chain 11	20000	2391	0.12	Down	0	0
MYH6	Myosin heavy chain 6	181	43	0.24	Down	1.78E-13	3.82E-05
MYH9	Myosin heavy chain 9	44458	13939	0.31	Down	0	0
MYLIP	Myosin regulatory light chain interacting protein	1272	564	0.44	Down	1.63E-46	5.22E-38
MYLK	Myosin light chain kinase	4068	1330	0.33	Down	4.73E-273	4.13E-263
MYO15A	Myosin XVA	484	145	0.30	Down	1.03E-32	2.87E-24
MYO18A	Myosin XVIIIA	23706	9069	0.38	Down	0	0
MYO5C	Myosin VC	1972	246	0.12	Down	0	0
MYO6	Myosin VI	9952	4436	0.45	Down	0	0
MYOF	Myosin F	12782	5295	0.41	Down	0	0
MYOM3	Myomesin 3	278	55	0.20	Down	6.95E-29	1.83E-19
N4BP2	NEDD4 binding protein 2	1483	480	0.32	Down	4.92E-99	2.25E-89
N4BP3	NEDD4 binding protein 3	2034	193	0.09	Down	0	0
NAALADL2	N-acetylated alpha-linked acidic dipeptidase like 2	597	178	0.30	Down	1.22E-42	3.74E-33
NAB1	NGFI-A binding protein 1	1480	659	0.45	Down	2.86E-55	9.79E-46
NAB2	NGFI-A binding protein 2	4907	1650	0.34	Down	0	0

NAPSA	Napsin A aspartic peptidase	465	125	0.27	Down	4.07E-37	1.18E-27
NBEAL2	Neurobeachin like 2	10096	2499	0.25	Down	0	0
NCAPD2	Non-SMC condensin I complex subunit D2	3388	1063	0.31	Down	2.90E-241	2.32E-230
NCAPG	Non-SMC condensin I complex subunit G	1947	572	0.29	Down	4.28E-149	2.49E-140
NCAPG2	Non-SMC condensin I complex subunit G2	3304	1514	0.46	Down	4.34E-119	2.20E-109
NCAPH	Non-SMC condensin I complex subunit H	1250	296	0.24	Down	1.83E-119	9.36E-111
NCK2	NCK adaptor protein 2	4972	2313	0.47	Down	2.39E-174	1.54E-164
NCKAP5	NCK associated protein 5	987	304	0.31	Down	1.52E-68	5.81E-60
NCOA2	Nuclear receptor coactivator 2	4398	1558	0.35	Down	5.88E-262	4.98E-252
NCOA7	Nuclear receptor coactivator 7	741	287	0.39	Down	2.52E-36	7.15E-26
NDC80	NDC80 kinetochore complex component	1003	303	0.30	Down	1.36E-72	5.31E-63
NEBL	Nebulette	1881	640	0.34	Down	1.72E-116	8.63E-108
NEIL3	Nei like DNA glycosylase 3	172	36	0.21	Down	1.23E-14	2.70E-06
NEK2	NIMA related kinase 2	499	99	0.20	Down	2.03E-54	6.91E-45
NEK5	NIMA related kinase 5	195	49	0.25	Down	8.04E-15	1.73E-05
NET1	Neuroepithelial cell transforming 1	13658	3247	0.24	Down	0	0
NFE2L3	NFE2 like bzip transcription factor 3	6458	1177	0.18	Down	0	0
NFKBIZ	NFKB inhibitor zeta	6136	1183	0.19	Down	0	0
NGEF	Neuronal guanine nucleotide exchange factor	1121	212	0.19	Down	3.16E-132	1.71E-121
NHS	NHS actin remodeling regulator	957	337	0.35	Down	4.23E-55	1.44E-45
NKD1	NKD inhibitor of WNT signaling pathway 1	1221	364	0.30	Down	1.28E-89	5.58E-82
NLRP10	NLR family pyrin domain containing 10	497	100	0.20	Down	1.53E-52	5.17E-44
NLRX1	NLR family member X1	3752	1422	0.38	Down	2.32E-199	1.65E-189
NOD1	Nucleotide binding oligomerization domain containing 1	2403	545	0.23	Down	3.05E-244	2.46E-234
NOS1	Nitric oxide synthase 1	217	18	0.08	Down	7.97E-37	2.30E-27
NOS3	Nitric oxide synthase 3	700	116	0.17	Down	5.08E-89	2.19E-79
NOTCH1	Neurogenic locus notch homolog protein 1	15478	3307	0.21	Down	0	0
NOTCH2	Neurogenic locus notch homolog protein 2	6180	1967	0.32	Down	0	0
NPY1R	Neuropeptide Y receptor Y1	164	34	0.21	Down	6.89E-15	1.49E-05
NR4A2	Nuclear receptor subfamily 4 group a member 2	1711	412	0.24	Down	2.60E-163	1.60E-153
NR5A2	Nuclear receptor subfamily 5 group a member 2	155	23	0.15	Down	1.71E-17	3.93E-09
NRG1	Neuregulin 1	525	77	0.15	Down	1.81E-71	7.02E-62
NRP1	Neuropilin 1	1901	848	0.45	Down	1.34E-70	5.21E-62
NUF2	NUF2 component of NDC80 kinetochore complex	818	369	0.45	Down	3.74E-28	9.78E-20
NUSAP1	Nucleolar and spindle associated protein 1	2244	689	0.31	Down	8.80E-164	5.41E-153
OCLN	Ocludin	2228	216	0.10	Down	0	0
ODZ2	Teneurin transmembrane protein 2	10962	893	0.08	Down	0	0
ODZ4	Teneurin transmembrane protein 4	2252	742	0.33	Down	3.68E-148	2.13E-138
OGDHL	Oxoglutarate dehydrogenase L	929	80	0.09	Down	1.22E-165	7.62E-157
OSBPL10	Oxysterol binding protein like 10	877	346	0.39	Down	6.32E-41	1.90E-31
OSR2	Odd-skipped related transciption factor 2	3357	990	0.29	Down	5.15E-258	4.31E-248
OTOF	Otoferlin	226	46	0.20	Down	3.08E-23	7.46E-13
P2RX7	Purinergic receptor P2X 7	870	341	0.39	Down	4.27E-42	1.29E-31
P2RY11	Purinergic receptor P2Y11	486	128	0.26	Down	6.69E-40	2.00E-30
PABPC1L	Poly(A) binding protein cytoplasmic 1 like	223	39	0.17	Down	5.62E-25	1.42E-15
PADI1	Peptidyl arginine deiminase 1	1134	136	0.12	Down	9.74E-177	6.32E-167
PADI3	Peptidyl arginine deiminase 3	1252	283	0.23	Down	3.51E-128	1.85E-116
PAFAH2	Platelet activating factor acetylhydrolase 2	414	156	0.38	Down	2.67E-20	6.22E-10

PAM	Peptidylglycine alpha-amidating monooxygenase	38990	15330	0.39	Down	0	0
PARD3	Par-3 family cell polarity regulator	5893	2076	0.35	Down	0	0
PARD6B	Par-6 family cell polarity regulator beta	489	115	0.24	Down	1.82E-44	5.75E-36
PATL2	PAT1 homolog 2	332	46	0.14	Down	1.56E-44	4.94E-37
PC	Pyruvate carboxylase	6331	2070	0.33	Down	0	0
PCDH1	Protocadherin 1	13146	2034	0.15	Down	0	0
PCDH12	Protocadherin 12	476	79	0.17	Down	2.05E-59	7.24E-50
PCDH7	Protocadherin 7	4514	763	0.17	Down	0	0
PCK2	Phosphoenolpyruvate carboxykinase 2. mitochondrial	7584	1450	0.19	Down	0	0
PCSK5	Proprotein convertase subtilisin/kexin type 5	873	390	0.45	Down	5.23E-31	1.42E-26
PCSK6	Proprotein convertase subtilisin/kexin type 6	3159	680	0.22	Down	0	0
PDE3A	Phosphodiesterase 3A	457	174	0.38	Down	3.22E-21	7.72E-12
PDLIM5	PDZ and LIM domain 5	2411	879	0.36	Down	9.56E-137	5.27E-126
PDZD2	PDZ domain containing 2	2623	561	0.21	Down	2.38E-281	2.13E-271
PDZD3	PDZ domain containing 3	90	7	0.08	Down	1.71E-13	3.58E-05
PDZD4	PDZ domain containing 4	434	159	0.37	Down	1.53E-21	3.68E-12
PDZK1	PDZ domain containing 1	4554	518	0.11	Down	0	0
PECAM1	Platelet and endothelial cell adhesion molecule 1	6095	1403	0.23	Down	0	0
PEG10	Paternally expressed 10	963	280	0.29	Down	4.09E-73	1.60E-63
PER1	Period circadian regulator 1	6951	2505	0.36	Down	0	0
PER2	Period circadian regulator 2	2324	819	0.35	Down	3.72E-138	2.07E-128
PERP	P53 apoptosis effector related to PMP22	95222	13288	0.14	Down	0	0
PHEX	Phosphate regulating endopeptidase X-linked	97	4	0.04	Down	4.46E-18	1.02E-08
PHF17	PHD finger protein 17	1124	508	0.45	Down	9.67E-40	2.88E-30
PHF19	PHD finger protein 19	469	143	0.30	Down	9.04E-32	2.47E-22
PHF7	PHD finger protein 7	438	138	0.32	Down	4.59E-28	1.20E-18
PHKA1	Phosphorylase kinase regulatory subunit alpha 1	2220	976	0.44	Down	5.90E-88	2.53E-77
PHLDB2	Pleckstrin homology like domain family B member 2	2930	999	0.34	Down	3.92E-185	2.62E-174
PHLPP2	PH domain and leucine rich repeat protein phosphatase 2	410	139	0.34	Down	3.56E-23	8.76E-15
PHYHIPL	Phytanoyl-coa 2-hydroxylase interacting protein like	1167	254	0.22	Down	1.48E-120	7.59E-112
PIGR	Polymeric immunoglobulin receptor	1764	153	0.09	Down	0	0
PIK3C2B	Phosphatidylinositol-4-phosphate 3-kinase catalytic subunit type 2 beta	6763	2127	0.31	Down	0	0
PIK3C2G	Phosphatidylinositol-4-phosphate 3-kinase catalytic subunit type 2 gamma	467	103	0.22	Down	4.17E-46	1.32E-36
PIM1	Pim-1 proto-oncogene. serine/threonine kinase	5431	1854	0.34	Down	0	0
PITPNM2	PITPNM family member 2	3616	1208	0.33	Down	5.95E-235	4.68E-225
PITPNM3	PITPNM family member 3	1296	180	0.14	Down	4.25E-187	2.88E-177
PKMYT1	Protein kinase. membrane associated tyrosine/threonine 1	658	244	0.37	Down	1.17E-32	3.26E-24
PKP1	Plakophilin 1	80120	10516	0.13	Down	0	0
PKP2	Plakophilin 2	1308	115	0.09	Down	4.29E-234	3.37E-224
PLA2G3	Phospholipase A2 group III	1844	599	0.32	Down	2.90E-123	1.51E-113
PLA2G4B	Phospholipase A2 group IVB	3898	1334	0.34	Down	1.43E-243	1.16E-234
PLA2G4D	Phospholipase A2 group IVD	508	171	0.34	Down	7.18E-30	1.92E-20
PLA2G4E	Phospholipase A2 group IVE	4368	1888	0.43	Down	1.45E-179	9.52E-171
PLA2G4F	Phospholipase A2 group IVF	2143	512	0.24	Down	6.54E-208	4.75E-197
PLAGL2	PLAG1 like zinc finger 2	865	379	0.44	Down	3.68E-32	1.01E-22
PLAUR	Plasminogen activator. urokinase receptor	3333	958	0.29	Down	2.20E-264	1.88E-254

PLB1	Phospholipase B1	1006	254	0.25	Down	3.57E-90	1.55E-80
PLBD1	Phospholipase B domain containing 1	12686	5117	0.40	Down	0	0
PLCB3	Phospholipase C beta 3	7195	2457	0.34	Down	0	0
PLCD1	Phospholipase C delta 1	17980	3663	0.20	Down	0	0
PLCE1	Phospholipase C epsilon 1	754	348	0.46	Down	3.33E-25	8.31E-15
PLCH1	Phospholipase C eta 1	697	57	0.08	Down	1.34E-125	7.09E-117
PLD2	Phospholipase D2	4514	1910	0.42	Down	3.25E-195	2.27E-185
PLEK2	Pleckstrin 2	1120	229	0.20	Down	4.55E-123	2.36E-113
PLEKHA4	Pleckstrin homology. myth4 and FERM domain containing A4	4835	2244	0.46	Down	2.02E-170	1.28E-161
PLEKHA7	Pleckstrin homology. myth4 and FERM domain containing A7	3662	541	0.15	Down	0	0
PLEKHD1	Pleckstrin homology. myth4 and FERM domain containing D1	194	48	0.25	Down	5.14E-15	1.11E-05
PLEKHG1	Pleckstrin homology. myth4 and FERM domain containing G1	1952	460	0.24	Down	9.45E-192	6.50E-181
PLEKHG4	Pleckstrin homology. myth4 and FERM domain containing G4	545	46	0.08	Down	4.02E-97	1.81E-87
PLEKHG5	Pleckstrin homology. myth4 and FERM domain containing G5	3772	958	0.25	Down	0	0
PLEKHG6	Pleckstrin homology. myth4 and FERM domain containing G6	2833	368	0.13	Down	0	0
PLEKHH1	Pleckstrin homology. myth4 and FERM domain containing H1	523	180	0.34	Down	9.55E-30	2.55E-20
PLEKHH3	Pleckstrin homology. myth4 and FERM domain containing H3	2581	824	0.32	Down	5.01E-178	3.26E-169
PLK3	Polo like kinase 3	2971	1032	0.35	Down	1.63E-180	1.07E-171
PLK4	Polo like kinase 4	1031	406	0.39	Down	7.30E-49	2.37E-39
PLS1	Plastin 1	520	145	0.28	Down	4.56E-40	1.36E-30
PLXNA2	Plexin A2	6018	1323	0.22	Down	0	0
PLXNA4	Plexin A4	185	45	0.24	Down	1.86E-13	3.98E-05
PLXNB2	Plexin B2	26994	11503	0.43	Down	0	0
PNLIPRP3	Pancreatic lipase related protein 3	404	71	0.18	Down	7.21E-48	2.32E-38
PNPLA1	Patatin like phospholipase domain containing 1	1444	240	0.17	Down	1.19E-185	8.04E-177
POC1B	POC1 centriolar protein B	777	352	0.45	Down	2.74E-26	7.01E-17
POR	Cytochrome p450 oxidoreductase	20553	5801	0.28	Down	0	0
PPARA	Peroxisome proliferator activated receptor alpha	711	121	0.17	Down	8.43E-89	3.63E-79
PPFIA1	PTPRF interacting protein alpha 1	11684	4327	0.37	Down	0	0
PPL	Periplakin	53331	15013	0.28	Down	0	0
PPP1R16B	Protein phosphatase 1 regulatory subunit 16B	277	88	0.32	Down	2.91E-16	6.45E-07
PPP2R2C	Protein phosphatase 2 regulatory subunit Bgamma	195	37	0.19	Down	5.77E-20	1.36E-10
PPP4R4	Protein phosphatase 4 regulatory subunit 4	1102	351	0.32	Down	1.79E-73	7.08E-65
PRC1	Protein regulator of cytokinesis 1	2072	462	0.22	Down	1.64E-212	1.21E-203
PRDM1	PR/SET domain 1	2161	523	0.24	Down	5.08E-206	3.68E-196
PREX2	Phosphatidylinositol-3.4.5-trisphosphate dependent Rac exchange factor 2	637	82	0.13	Down	2.81E-94	1.25E-84
PRICKLE1	Prickle planar cell polarity protein 1	2690	911	0.34	Down	1.58E-169	1.00E-160
PRKCH	Protein kinase C eta	5095	1345	0.26	Down	0	0
PRKCI	Protein kinase C iota	2678	696	0.26	Down	9.03E-238	7.15E-228
PRKCQ	Protein kinase C theta	262	66	0.25	Down	4.68E-21	1.12E-11
PRKD1	Protein kinase D1	1083	368	0.34	Down	1.92E-65	7.13E-57
PRLR	Prolactin receptor	1741	300	0.17	Down	4.11E-220	3.09E-210
PRND	Prion like protein doppel	188	7	0.04	Down	2.16E-39	6.41E-30
PRNP	Prion protein (Kanno blood group)	11620	2489	0.21	Down	0	0

PROC	Protein C. inactivator of coagulation factors Va and viiia	337	57	0.17	Down	1.63E-39	4.89E-31
PRODH	Proline dehydrogenase 1	1014	385	0.38	Down	2.03E-51	6.73E-42
PROKR2	Prokineticin receptor 2	880	88	0.10	Down	1.74E-147	1.01E-138
PRSS35	Serine protease 35	1453	59	0.04	Down	0	0
PRSS36	Serine protease 36	3561	775	0.22	Down	0	0
PRSS53	Serine protease 53	511	128	0.25	Down	1.39E-43	4.33E-35
PSD4	Pleckstrin and Sec7 domain containing 4	7417	2220	0.30	Down	0	0
PSP	Persephin	411	14	0.03	Down	6.46E-93	2.84E-82
PTCH1	Patched 1	2227	781	0.35	Down	2.75E-133	1.50E-124
PTCH2	Patched 2	1884	266	0.14	Down	8.15E-272	7.07E-261
PTGFRN	Prostaglandin F2 receptor inhibitor	6891	1647	0.24	Down	0	0
PTH1R	Parathyroid hormone 1 receptor	1156	226	0.20	Down	6.56E-133	3.55E-128
PTHLH	Parathyroid hormone like hormone	346	114	0.33	Down	5.76E-20	1.36E-10
PTK6	Protein tyrosine kinase 6 (inactive)	834	152	0.18	Down	1.31E-99	6.01E-90
PTK7	Protein tyrosine kinase 7 (inactive)	14723	2915	0.20	Down	0	0
PTPN14	Protein tyrosine phosphatase non-receptor type 14	2098	605	0.29	Down	1.19E-163	7.37E-155
PTPN4	Protein tyrosine phosphatase non-receptor type 4	749	199	0.27	Down	1.19E-61	4.29E-53
PTPRF	Protein tyrosine phosphatase receptor type F	43427	6977	0.16	Down	0	0
PTPRK	Protein tyrosine phosphatase receptor type K	9932	1270	0.13	Down	0	0
PTPRN2	Protein tyrosine phosphatase receptor type N2	966	157	0.16	Down	1.02E-125	5.36E-116
PTPRZ1	Protein tyrosine phosphatase receptor type Z1	6547	1599	0.24	Down	0	0
PVRL2	Nectin cell adhesion molecule 2	2564	529	0.21	Down	1.48E-282	1.32E-273
PVRL4	Nectin cell adhesion molecule 4	4952	733	0.15	Down	0	0
RAB11FIP4	Rab11 family interacting protein 4	1663	404	0.24	Down	3.06E-157	1.83E-147
RACGAP1	Rac gtpase Activating Protein 1	1537	376	0.24	Down	4.51E-145	2.56E-134
RAD54B	Rad54 homolog b	578	104	0.18	Down	1.06E-67	4.01E-59
RALGAPA2	Ral gtpase Activating Protein Catalytic Subunit Alpha 2	2391	689	0.29	Down	3.12E-189	2.12E-178
RALGPS2	Ral gef with ph domain and sh3 binding motif 2	1818	844	0.46	Down	4.21E-62	1.51E-52
RAPGEF5	Rap guanine nucleotide exchange factor 5	373	55	0.15	Down	1.24E-48	4.04E-40
RAPGEFL1	Rap guanine nucleotide exchange factor like 1	3370	608	0.18	Down	0	0
RASA4	Ras p21 protein activator 4	751	269	0.36	Down	2.50E-42	7.56E-33
RASGRP1	Ras guanyl releasing protein 1	951	195	0.21	Down	9.08E-104	4.26E-94
RASSF9	Ras association domain family member 9	705	144	0.20	Down	2.40E-76	9.60E-67
RCC1	Regulator of chromosome condensation 1	4308	1301	0.30	Down	0	0
RECQL4	Recq Like Helicase 4	576	121	0.21	Down	2.84E-61	1.01E-50
RELN	Reelin	1078	467	0.43	Down	5.71E-42	1.74E-32
RET	Ret proto-oncogene	3932	849	0.22	Down	0	0
RFX2	Regulatory factor x2	981	333	0.34	Down	5.60E-60	1.98E-50
RGN	Regucalcin	1218	385	0.32	Down	1.54E-83	6.46E-74
RGS12	Regulator of g protein signaling 12	4654	1954	0.42	Down	1.39E-203	1.00E-194
RHBDL1	Rhomboid like 1	431	96	0.22	Down	6.80E-42	2.07E-32
RHBDL3	Rhomboid like 3	155	26	0.17	Down	7.46E-17	1.67E-07
RIN1	Ras and rab interactor 1	837	317	0.38	Down	4.55E-42	1.39E-32
RNF180	Ring finger protein 180	345	75	0.22	Down	1.20E-33	3.34E-25
RNF43	Ring finger protein 43	1347	234	0.17	Down	1.26E-167	7.89E-159
RNFT1	Ring finger protein. transmembrane 1	422	183	0.43	Down	2.49E-14	5.32E-05
ROBO2	Roundabout guidance receptor 2	1739	316	0.18	Down	1.39E-210	1.03E-201

ROD1	Polypyrimidine tract binding protein 3	2521	974	0.39	Down	1.75E-127	9.34E-119
ROR2	Receptor tyrosine kinase like orphan receptor 2	1627	518	0.32	Down	1.61E-110	7.83E-103
RORC	Rar related orphan receptor c	1963	190	0.10	Down	0	0
RREB1	Ras responsive element binding protein 1	5321	1890	0.36	Down	0	0
RRM2	Ribonucleotide reductase regulatory subunit m2	1337	354	0.26	Down	8.67E-115	4.30E-111
RRP12	Ribosomal rna processing 12 homolog	5001	2257	0.45	Down	2.47E-188	1.68E-178
RSPO3	R-spondin 3	207	42	0.20	Down	3.91E-20	9.23E-11
RTKN2	Rhotekin 2	238	65	0.27	Down	5.89E-19	1.32E-07
RTN4R	Reticulon 4 receptor	1855	819	0.44	Down	1.87E-71	7.26E-62
RUFY4	Run and fyve domain containing 4	230	53	0.23	Down	5.15E-21	1.21E-10
RUNDC3B	Run domain containing 3b	451	47	0.10	Down	4.04E-73	1.58E-63
RYR2	Ryanodine receptor 2	253	75	0.30	Down	3.06E-17	6.77E-07
S100A6	S100 calcium binding protein a6;	18079	6625	0.37	Down	0	0
S100A7	S100 calcium binding protein a7; psoriasin	54034	16742	0.31	Down	0	0
S1PR1	Sphingosine-1-phosphate receptor 1	766	194	0.25	Down	1.14E-67	4.30E-58
SAA1	Serum amyloid a1	341	107	0.31	Down	3.16E-21	7.58E-12
SAMD11	Sterile alpha motif domain containing 11	413	92	0.22	Down	5.33E-40	1.59E-30
SATB2	Satb homeobox 2	694	156	0.22	Down	7.00E-69	2.66E-59
SBF1	Set binding factor 1	17216	7563	0.44	Down	0	0
SCD	Stearoyl-coa Desaturase	286981	11735	0.04	Down	0	0
SCEL	Sciellin	12098	2140	0.18	Down	0	0
SCNN1B	Sodium channel epithelial 1 subunit beta	2081	342	0.16	Down	2.13E-272	1.86E-262
SCUBE3	Signal peptide. cub domain and egf like domain containing 3	450	73	0.16	Down	6.94E-57	2.40E-47
SEC14L4	Sec14 like lipid binding 4	2192	207	0.09	Down	0	0
SEC16B	Sec16 homolog b. endoplasmic reticulum export factor	342	65	0.19	Down	1.39E-36	4.05E-28
SECISBP2L	Secis binding protein 2 like	6935	2123	0.31	Down	0	0
SEL1L3	Sel1l family member 3	2659	831	0.31	Down	6.29E-190	4.28E-179
SELE	Selectin E	220	40	0.18	Down	7.94E-24	1.97E-14
SELI	Selenoprotein I	19498	8292	0.43	Down	0	0
SEMA3E	Semaphorin 3E	418	157	0.38	Down	1.20E-19	2.82E-10
SEMA3F	Semaphorin 3F	4554	674	0.15	Down	0	0
SEMA3G	Semaphorin 3G	550	82	0.15	Down	2.89E-74	1.14E-64
SEMA4F	Semaphorin 4F	2242	1009	0.45	Down	2.74E-83	1.14E-73
SEMA4G	Semaphorin 4G	943	138	0.15	Down	3.77E-131	2.04E-121
SEMA5A	Semaphorin 5A	1159	284	0.25	Down	1.09E-107	5.21E-98
SEPX1	Methionine sulfoxide reductase b1	2725	1216	0.45	Down	6.62E-105	3.11E-95
SERINC2	Serine incorporator 2	8271	956	0.12	Down	0	0
SERPINB10	Serpin = serin-proteinase-inhibitor b10	19974	4912	0.25	Down	0	0
SERPINB12	Serpin = serin-proteinase-inhibitor b12	4512	2043	0.45	Down	2.36E-168	1.48E-159
SERPINB13	Serpin = serin-proteinase-inhibitor b13	969	224	0.23	Down	6.90E-95	3.08E-85
SERPINB2	Serpin = serin-proteinase-inhibitor b2	12171	2459	0.20	Down	0	0
SERPINB5	Serpin = serin-proteinase-inhibitor b5	25039	5458	0.22	Down	0	0
SERPINB7	Serpin = serin-proteinase-inhibitor b7	6732	1982	0.29	Down	0	0
SETBP1	Set binding protein 1	3321	1212	0.36	Down	8.36E-189	5.67E-178
SGMS1	Sphingomyelin synthase 1	9119	3277	0.36	Down	0	0
SGPP2	Sphingosine-1-phosphate phosphatase 2	1853	532	0.29	Down	7.48E-146	4.29E-136
SGSM1	Small g protein signaling modulator 1	289	57	0.20	Down	3.02E-30	8.12E-21
SH2D3A	Sh2 domain containing 3a	626	134	0.21	Down	1.09E-63	3.99E-55

SH2D4A	Sh2 domain containing 4a	792	196	0.25	Down	7.92E-72	3.08E-63
SH3BP1	Sh3 domain binding protein 1	5804	2659	0.46	Down	1.41E-211	1.04E-201
SH3D19	Sh3 domain containing 19	4135	1580	0.38	Down	6.89E-217	5.15E-207
SH3GLB2	Sh3 domain containing grb2 like. endophilin b2	7920	2971	0.38	Down	0	0
SH3PXD2A	Sh3 and px domains 2a	3470	1205	0.35	Down	1.36E-211	1.01E-202
SH3RF2	Sh3 domain containing ring finger 2	4150	709	0.17	Down	0	0
SH3TC2	Sh3 domain and tetratricopeptide repeats 2	1013	372	0.37	Down	1.62E-53	5.51E-45
SHANK3	Sh3 and multiple ankyrin repeat domains 3	1693	436	0.26	Down	1.10E-149	6.44E-141
SHCBP1	Shc binding and spindle associated 1	514	128	0.25	Down	3.73E-46	1.17E-35
SHROOM3	Shroom family member 3	1620	192	0.12	Down	2.52E-256	2.10E-245
SIAH2	Siah e3 ubiquitin protein ligase 2	3881	1738	0.45	Down	4.37E-148	2.53E-138
SIK1	Salt inducible kinase 1	6761	2170	0.32	Down	0	0
SIK2	Salt inducible kinase 2	1927	693	0.36	Down	2.37E-110	1.15E-100
SIM2	Sim bhlh transcription factor 2	521	5	0.01	Down	8.43E-135	4.63E-126
SIPA1L1	Signal induced proliferation associated 1 like 1	7453	2042	0.27	Down	0	0
SIX4	Six homeobox 4	198	38	0.19	Down	4.04E-20	9.53E-11
SKA3	Spindle and kinetochore associated complex subunit 3	542	214	0.39	Down	5.62E-24	1.40E-14
SLC10A3	Solute carrier family 10 member 3	10413	2862	0.27	Down	0	0
SLC11A2	Solute carrier family 11 member a2	1061	442	0.42	Down	5.91E-45	1.85E-35
SLC16A10	Solute carrier family 16 member a10	341	34	0.10	Down	1.50E-54	5.14E-46
SLC16A14	Solute carrier family 16 member a14	348	122	0.35	Down	4.85E-18	1.11E-08
SLC16A5	Solute carrier family 16 member a5	3866	1783	0.46	Down	9.81E-138	5.46E-128
SLC16A6	Solute carrier family 16 member a6	657	106	0.16	Down	6.86E-85	2.89E-75
SLC16A7	Solute carrier family 16 member a7	463	106	0.23	Down	4.74E-44	1.47E-35
SLC16A9	Solute carrier family 16 member a9	653	124	0.19	Down	7.45E-76	2.95E-65
SLC1A3	Solute carrier family 1 member a3	553	59	0.11	Down	1.86E-88	8.06E-80
SLC22A15	Solute carrier family 22 member a15	566	166	0.29	Down	3.75E-41	1.13E-31
SLC22A23	Solute carrier family 22 member a23	3916	596	0.15	Down	0	0
SLC22A3	Solute carrier family 22 member a3	1240	100	0.08	Down	6.68E-229	5.19E-219
SLC22A5	Solute carrier family 22 member a5	2003	468	0.23	Down	1.93E-196	1.36E-187
SLC26A2	Solute carrier family 26 member a2	1070	231	0.22	Down	5.23E-112	2.56E-102
SLC26A7	Solute carrier family 26 member a7	554	78	0.14	Down	1.69E-76	6.82E-68
SLC26A9	Solute carrier family 26 member a9	616	226	0.37	Down	6.51E-32	1.78E-22
SLC27A4	Solute carrier family 27 member a4	23618	4976	0.21	Down	0	0
SLC2A1	Solute carrier family 2 member a1	23767	6156	0.26	Down	0	0
SLC2A10	Solute carrier family 2 member a10	863	145	0.17	Down	1.88E-108	9.06E-100
SLC2A5	Solute carrier family 2 member a5	439	72	0.16	Down	5.43E-55	1.85E-45
SLC35F3	Solute Carrier Family 35 memberf3	315	41	0.13	Down	1.24E-43	3.87E-35
SLC36A4	Solute carrier family 36 member a4	830	294	0.35	Down	6.78E-48	2.16E-38
SLC38A1	Solute carrier family 38 member a1	412	62	0.15	Down	1.82E-54	6.18E-45
SLC38A2	Solute carrier family 38 member a2	5985	1405	0.23	Down	0	0
SLC39A11	Solute carrier family 39 member a11	2011	378	0.19	Down	5.48E-239	4.36E-229
SLC41A1	Solute carrier family 41 member a1	1765	791	0.45	Down	1.54E-64	5.70E-56
SLC43A1	Solute carrier family 43 member a1	10025	575	0.06	Down	0	0
SLC43A3	Solute carrier family 43 member a3	371	114	0.31	Down	3.82E-24	9.51E-15
SLC45A3	Solute carrier family 45 member a3	1031	91	0.09	Down	1.44E-182	9.62E-175
SLC47A2	Solute carrier family 47 member a2	140	23	0.16	Down	3.94E-16	8.54E-06
SLC4A1	Solute carrier family 4 member a1	136	18	0.13	Down	4.50E-17	1.01E-07

SLC4A11	Solute carrier family 4 member a11	3005	590	0.20	Down	0	0
SLC4A9	Solute carrier family 4 member a9	145	19	0.13	Down	1.47E-17	3.38E-09
SLC5A1	Solute carrier family 5 member a1	3328	541	0.16	Down	0	0
SLC5A10	Solute carrier family 5 member a10	434	83	0.19	Down	2.34E-50	7.57E-39
SLC5A8	Solute carrier family 5 member a8	437	153	0.35	Down	1.38E-22	3.41E-14
SLC6A14	Solute carrier family 6 member a14	2121	542	0.26	Down	3.96E-191	2.73E-181
SLC6A20	Solute carrier family 6 member a20	1451	424	0.29	Down	4.49E-111	2.18E-101
SLC6A9	Solute carrier family 6 member a9	3633	809	0.22	Down	0	0
SLC7A1	Solute carrier family 7 member a1	1644	273	0.17	Down	4.67E-213	3.45E-203
SLC7A10	Solute carrier family 7 member a10	211	23	0.11	Down	1.17E-30	3.20E-22
SLC9A2	Solute carrier family 9 member a2	392	52	0.13	Down	8.90E-56	3.06E-46
SLCO1A2	Solute carrier organic anion transporter family member 1a2	361	13	0.04	Down	1.70E-81	6.92E-70
SLCO2B1	Solute carrier organic anion transporter family member 2b1	5750	2086	0.36	Down	0	0
SLCO4A1	Solute carrier organic anion transporter family member 4a1	503	157	0.31	Down	3.49E-33	9.69E-24
SLIT3	Slit guidance ligand 3	11214	4217	0.38	Down	0	0
SMAD1	Smad family member 1	4430	1738	0.39	Down	8.39E-222	6.36E-212
SMAD7	Smad family member 7	402	167	0.42	Down	4.94E-15	1.07E-05
SMTN	Smoothelin	7264	1307	0.18	Down	0	0
SNCAIP	Synuclein alpha interacting protein	816	151	0.19	Down	2.88E-96	1.30E-86
SNED1	Sushi. nidogen and egf like domains 1	483	73	0.15	Down	2.98E-64	1.09E-54
SNTB1	Syntrophin beta 1	551	214	0.39	Down	2.88E-25	7.28E-16
SORCS1	Sortilin related vps10 domain containing receptor 1	194	10	0.05	Down	6.93E-38	2.02E-28
SORL1	Sortilin related receptor 1	4455	1147	0.26	Down	0	0
SOX2	SRY-box transcription factor 2	684	18	0.03	Down	1.03E-160	6.30E-152
SOX7	SRY-box transcription factor 7	505	79	0.16	Down	7.73E-66	2.86E-58
SOX9	SRY-box transcription factor 9	4210	458	0.11	Down	0	0
SP6	Sp6 transcription factor	1326	256	0.19	Down	2.69E-153	1.59E-143
SPAG5	Sperm associated antigen 5	1535	472	0.31	Down	3.67E-110	1.78E-100
SPAM1	Sperm adhesion molecule 1	106	0	0.00	Down	4.94E-26	1.26E-16
SPARC	Secreted protein acidic and rich in cysteine; Osteonectin	195032	87689	0.45	Down	0	0
SPARCL1	Sparc like 1	11986	4394	0.37	Down	0	0
SPEG	Striated muscle enriched protein kinase	2327	514	0.22	Down	3.19E-242	2.57E-232
SPHK1	Sphingosine kinase 1	5090	999	0.20	Down	0	0
SPINK5	Serine peptidase inhibitor kazal type 5	196864	43974	0.22	Down	0	0
SPIRE2	Spire type actin nucleation factor 2	2301	370	0.16	Down	0	0
SPNS2	Spns lysolipid transporter 2. sphingosine-1-phosphate	767	131	0.17	Down	8.32E-96	3.73E-86
SPOCK2	Sparc (osteonectin). cwcv and kazal like domains proteoglycan 2	246	62	0.25	Down	1.52E-18	3.55E-10
SPTBN5	Spectrin beta. non-erythrocytic 5	602	53	0.09	Down	6.39E-106	3.03E-96
SPTLC3	Serine palmitoyltransferase long chain base subunit 3	3148	1064	0.34	Down	1.15E-199	8.20E-191
SREBF1	Sterol regulatory element binding transcription factor 1	15077	4192	0.28	Down	0	0
SRMS	Src-related kinase lacking c-terminal regulatory tyrosine and n-terminal myristylation sites	479	33	0.07	Down	4.19E-92	1.83E-81
SSFA2	Itpr interacting domain containing 2	12557	4568	0.36	Down	0	0
ST6GAL1	St6 beta-galactoside alpha-2.6-sialyltransferase 1	6745	3126	0.46	Down	1.01E-238	8.03E-230
ST6GALNAC6	St6 n-acetylgalactosaminide alpha-2.6-sialyltransferase 6	2327	1077	0.46	Down	5.58E-81	2.30E-71
STAB1	Stabilin 1	6557	3035	0.46	Down	1.14E-232	8.94E-224

STAB2	Stabilin 2	501	126	0.25	Down	1.87E-42	5.79E-34
STAG3	Stag3 cohesin complex component	1647	508	0.31	Down	6.12E-118	3.09E-108
STON1	Stonin 1	2459	869	0.35	Down	1.04E-144	5.93E-137
STON2	Stonin 2	484	47	0.10	Down	3.40E-81	1.41E-71
STRN	Striatin	2548	1001	0.39	Down	5.56E-126	2.93E-116
SULT1E1	Sulfotransferase family 1e member 1	434	89	0.21	Down	1.45E-44	4.57E-36
SUSD2	Sushi domain containing 2	2805	543	0.19	Down	0	0
SUSD5	Sushi domain containing 5	95	0	0.00	Down	6.92E-24	1.69E-13
SUV420H2	Lysine methyltransferase 5c	630	248	0.39	Down	1.36E-27	3.59E-19
SVOPL	Svop like	369	84	0.23	Down	1.27E-33	3.59E-25
SYCP1	Synaptonemal complex protein 1	254	34	0.13	Down	9.65E-35	2.72E-25
SYNE2	Spectrin repeat containing nuclear envelope protein 2	5502	912	0.17	Down	0	0
SYNJ2	Synaptojanin 2	1249	426	0.34	Down	1.13E-75	4.53E-67
SYT7	Synaptotagmin 7	1244	140	0.11	Down	2.64E-200	1.88E-190
SYTL1	Synaptotagmin like 1	11131	1697	0.15	Down	0	0
SYTL3	Synaptotagmin like 3	401	64	0.16	Down	6.81E-51	2.25E-41
TACC3	Transforming acidic coiled-coil containing protein 3	2129	801	0.38	Down	2.94E-113	1.45E-103
TAF4B	TATA-Box Binding Protein Associated Factor 4b	590	174	0.29	Down	1.01E-42	3.10E-33
TAGLN	Transgelin	14158	3709	0.26	Down	0	0
TANC2	Tetratricopeptide repeat. ankyrin repeat and coiled-coil containing 2	1463	385	0.26	Down	9.27E-128	4.90E-117
TBX18	T-box transcription factor 18	210	56	0.27	Down	4.95E-15	1.07E-05
TC2N	Tandem c2 domains. nuclear	743	167	0.22	Down	5.57E-75	2.19E-65
TCF3	Transcription factor 3	5241	2057	0.39	Down	9.47E-254	8.04E-253
TEAD3	Tea domain transcription factor 3	3642	1108	0.30	Down	2.84E-270	2.45E-259
TEAD4	Tea domain transcription factor 4	1099	252	0.23	Down	6.89E-109	3.32E-99
TEKT5	Tektin 5	2084	250	0.12	Down	0	0
TET2	Tet methylcytosine dioxygenase 2	2350	1093	0.47	Down	7.84E-82	3.23E-71
TET3	Tet methylcytosine dioxygenase 3	2512	418	0.17	Down	0	0
TFRC	Transferrin receptor	3431	1587	0.46	Down	4.81E-121	2.47E-111
TGFBRAP1	Transforming growth factor. beta receptor associated protein 1	1311	577	0.44	Down	6.40E-50	2.09E-40
TGM3	Transglutaminase 3	32649	2030	0.06	Down	0	0
TGM6	Transglutaminase 6	197	46	0.23	Down	2.16E-16	4.81E-07
TGM7	Transglutaminase 7	241	22	0.09	Down	9.79E-40	2.91E-30
THBS1	Thrombospondin 1	12550	4746	0.38	Down	0	0
THBS3	Thrombospondin 3	3607	1154	0.32	Down	3.01E-249	2.47E-239
THRB	Thyroid hormone receptor beta	644	269	0.42	Down	7.95E-27	2.02E-16
THSD1	Thrombospondin type 1 domain containing 1	18246	4474	0.25	Down	0	0
THSD4	Thrombospondin type 1 domain containing 4	505	88	0.17	Down	4.68E-61	1.67E-51
TIAM1	Tiam rac1 associated gef 1	18537	3998	0.22	Down	0	0
TIAM2	Tiam rac1 associated gef 2	1618	370	0.23	Down	3.35E-162	2.05E-152
TICAM1	Tir domain containing adaptor molecule 1	1973	648	0.33	Down	5.01E-131	2.69E-120
TIMELESS	Timeless circadian regulator	1424	369	0.26	Down	2.09E-125	1.10E-115
TIMP3	Timp metallopeptidase inhibitor 3	8347	3674	0.44	Down	0	0
TINAGL1	Tubulointerstitial nephritis antigen like 1	1675	262	0.16	Down	7.26E-226	5.58E-216
TJP3	Tight junction protein 3	480	70	0.15	Down	2.75E-65	1.01E-55
TLE1	Tle family member 1. transcriptional corepressor	4327	1334	0.31	Down	0	0
TLE3	Tle family member 3. transcriptional corepressor	10083	3351	0.33	Down	0	0

TLL1	Tolloid like 1	169	35	0.21	Down	1.82E-17	3.96E-06
TM7SF2	Transmembrane 7 superfamily member 2	15522	1905	0.12	Down	0	0
TMC5	Transmembrane channel like 5	139	14	0.10	Down	1.95E-19	4.63E-11
TMEM132A	Transmembrane protein 132a	4290	1289	0.30	Down	0	0
TMEM132C	Transmembrane protein 132c	1582	361	0.23	Down	7.67E-159	4.62E-149
TMEM135	Transmembrane protein 135	2776	907	0.33	Down	1.46E-184	9.78E-176
TMEM2	Transmembrane protein 2	1976	721	0.36	Down	1.51E-109	7.33E-102
TMEM63B	Transmembrane protein 63b	13838	5260	0.38	Down	0	0
TMEM79	Transmembrane protein 79	6897	1161	0.17	Down	0	0
TMOD3	Tropomodulin 3	2126	694	0.33	Down	1.38E-142	7.77E-132
TMPRSS11A	Transmembrane serine protease 11a	1039	29	0.03	Down	2.34E-245	1.90E-235
TMPRSS11B	Transmembrane serine protease 11b	792	10	0.01	Down	5.83E-203	4.19E-193
TMPRSS11D	Transmembrane serine protease 11d	2253	52	0.02	Down	0	0
TMPRSS13	Transmembrane serine protease 13	3059	469	0.15	Down	0	0
TMPRSS4	Transmembrane serine protease 4	1206	233	0.19	Down	4.63E-139	2.59E-129
TMTC2	Transmembrane o-mannosyltransferase targeting cadherins 2	410	152	0.37	Down	1.05E-19	2.47E-10
TNFAIP1	Tnf alpha induced protein 1	2343	1063	0.45	Down	1.50E-84	6.33E-79
TNFRSF11B	TNF Receptor Superfamily Member 11b	271	26	0.10	Down	3.53E-44	1.10E-34
TNFRSF19	Tnf receptor superfamily member 19	634	272	0.43	Down	7.42E-24	1.84E-14
TNIK	Traf2 and nck interacting kinase	1127	303	0.27	Down	1.32E-93	5.87E-85
TNK1	Tyrosine kinase non receptor 1	1076	152	0.14	Down	2.76E-153	1.63E-143
TNKS1BP1	Tankyrase 1 binding protein 1	21521	6112	0.28	Down	0	0
TNN	Tenascin N	670	89	0.13	Down	1.26E-96	5.69E-88
TNS4	Tensin 4	4710	919	0.20	Down	0	0
TOX3	Tox high mobility group box family member 3	231	35	0.15	Down	6.84E-29	1.80E-19
TP63	Tumor protein p63	9255	1298	0.14	Down	0	0
TP73	Tumor protein p73	2452	206	0.08	Down	0	0
TPX2	Tpx2 microtubule nucleation factor	2763	719	0.26	Down	4.47E-245	3.62E-235
TRABD	Trab Domain Containing	2642	1168	0.44	Down	8.57E-103	4.01E-93
TRAF4	Tnf receptor associated factor 4	4554	1039	0.23	Down	0	0
TRAF5	Tnf receptor associated factor 5	705	182	0.26	Down	1.00E-61	3.56E-51
TRIB1	Tribbles pseudokinase 1	1532	511	0.33	Down	5.19E-99	2.36E-88
TRIM13	Tripartite motif containing 13	1892	854	0.45	Down	2.59E-69	9.88E-60
TRIM2	Tripartite motif containing 2	3514	1629	0.46	Down	2.20E-123	1.14E-113
TRIM29	Tripartite motif containing 29	56435	7235	0.13	Down	0	0
TRIP13	Thyroid hormone receptor interactor 13	396	126	0.32	Down	1.13E-23	2.84E-15
TROAP	Trophinin associated protein	737	239	0.32	Down	1.83E-47	5.87E-38
TRPC3	Transient receptor potential cation channel subfamily c member 3	81	2	0.02	Down	5.83E-16	1.28E-06
TRPC4	Transient receptor potential cation channel subfamily c member 4	171	34	0.20	Down	2.44E-16	5.43E-07
TRPC6	Transient receptor potential cation channel subfamily c member 6	635	181	0.29	Down	2.56E-49	8.27E-39
TRPM3	Transient receptor potential cation channel subfamily m member 3	208	39	0.19	Down	9.34E-22	2.26E-12
TRPM6	Transient receptor potential cation channel subfamily m member 6	192	42	0.22	Down	4.52E-18	1.02E-07
TRPS1	Transcriptional repressor gata binding 1	6236	2570	0.41	Down	1.64E-284	1.47E-275
TRPV4	Transient receptor potential cation channel subfamily v member 4	2377	374	0.16	Down	0	0
TRPV6	Transient receptor potential cation channel subfamily v member 6	3843	403	0.10	Down	0	0

TSPEAR	Thrombospondin type laminin g domain and ear repeats	497	42	0.08	Down	3.13E-89	1.35E-78
TTC38	Tetratricopeptide repeat domain 38	22047	5726	0.26	Down	0	0
TTC39C	Tetratricopeptide repeat domain 39c	1922	663	0.34	Down	1.29E-116	6.51E-108
TTC7A	Tetratricopeptide repeat domain 7a	5258	2404	0.46	Down	2.65E-192	1.83E-182
TTK	Ttk protein kinase	735	196	0.27	Down	3.69E-61	1.32E-51
TTPAL	Alpha tocopherol transfer protein like	459	190	0.41	Down	8.39E-18	1.91E-08
TUB	Tub bipartite transcription factor	450	75	0.17	Down	8.08E-56	2.78E-46
TUBA4A	Tubulin Alpha 4a	43395	17864	0.41	Down	0	0
TXNDC5	Thioredoxin domain containing 5	8505	3561	0.42	Down	0	0
UBE2C	Ubiquitin conjugating enzyme e2 c	2096	544	0.26	Down	1.02E-184	6.86E-176
UBQLN1	Ubiquilin 1	11448	4924	0.43	Down	0	0
UEVLD	Uev and lactate/malate dehyrogenase domains	742	300	0.40	Down	1.87E-31	5.15E-23
UGGT1	Udp-glucose glycoprotein glucosyltransferase 1	3272	1210	0.37	Down	6.00E-181	3.94E-171
UGT1A1	Udp glucuronosyltransferase family 1 member a1	138	13	0.09	Down	6.46E-23	1.54E-11
UGT8	Udp glycosyltransferase 8	273	41	0.15	Down	5.54E-36	1.57E-25
UHRF1	Ubiquitin like with phd and ring finger domains 1	1322	219	0.17	Down	5.32E-171	3.38E-161
UHRF1BP1	Bridge-like lipid transfer protein family member 3a	1429	491	0.34	Down	3.53E-87	1.51E-77
UHRF1BP1L	Bridge-Like Lipid Transfer Protein Family Member 3A like	9937	3191	0.32	Down	0	0
ULK1	Unc-51 like autophagy activating kinase 1	7324	3083	0.42	Down	0	0
UNC13D	Unc-13 homolog d	782	201	0.26	Down	4.84E-68	1.83E-58
UNC5B	Unc-5 netrin receptor b	3887	748	0.19	Down	0	0
UNC93A	Unc-93 homolog a	2372	1064	0.45	Down	5.66E-90	2.44E-79
USP6NL	Usp6 n-terminal like	2990	1030	0.34	Down	4.77E-185	3.19E-175
VANGL1	Vangl planar cell polarity protein 1	2964	1111	0.37	Down	6.64E-160	4.01E-150
VAV3	Vav guanine nucleotide exchange factor 3	2573	821	0.32	Down	1.34E-176	8.71E-172
VDR	Vitamin d receptor	3670	439	0.12	Down	0	0
VEGFA	Vascular endothelial growth factor alpha	717	295	0.41	Down	4.12E-30	1.10E-20
VILL	Villin like	1379	252	0.18	Down	1.93E-165	1.20E-156
VNN1	Vanin 1	538	137	0.25	Down	2.88E-46	9.13E-37
VSIG10	V-set and immunoglobulin domain containing 10	600	182	0.30	Down	7.08E-42	2.15E-32
VWA2	Von willebrand factor a domain containing 2	1016	140	0.14	Down	1.17E-145	6.74E-137
VWF	Von willebrand factor	8822	1268	0.14	Down	0	0
WDFY2	Wd repeat and fyve domain containing 2	750	339	0.45	Down	2.10E-26	5.33E-16
WDR45L	Wd repeat domain 45b	4846	2092	0.43	Down	3.51E-203	2.51E-192
WEE2	Wee2 oocyte meiosis inhibiting kinase	508	132	0.26	Down	1.62E-42	4.96E-33
WHAMM	Wasp homolog associated with actin. golgi membranes and microtubules	1849	843	0.46	Down	4.53E-67	1.68E-56
WNT10B	Wnt family member 10b	484	147	0.30	Down	4.48E-33	1.24E-23
WNT3	Wnt family member 3	1961	553	0.28	Down	6.46E-158	3.88E-148
WNT4	Wnt family member 4	2120	357	0.17	Down	5.16E-273	4.51E-263
WWC1	Ww and c2 domain containing 1	2980	412	0.14	Down	0	0
WWTR1	Ww domain containing transcription regulator 1	2967	1127	0.38	Down	2.07E-156	1.24E-146
XKRX	Xk related x-linked	682	191	0.28	Down	2.69E-53	9.06E-44
YAP1	Yes1 associated transcriptional regulator	3324	1539	0.46	Down	6.70E-117	3.36E-107
YES1	Yes proto-oncogene 1. src family tyrosine kinase	3786	769	0.20	Down	0	0
YOD1	Yod1 deubiquitinase	2533	902	0.36	Down	2.29E-148	1.33E-138
ZBED4	Zinc finger bed-type containing 4	2471	1042	0.42	Down	3.06E-106	1.45E-97
ZBTB16	Zinc finger and btb domain containing 16	1044	486	0.47	Down	4.91E-35	1.38E-24

ZBTB43	Zinc finger and btb domain containing 43	673	275	0.41	Down	1.77E-27	4.66E-19
ZBTB7B	Zinc finger and btb domain containing 7b	9568	2339	0.24	Down	0	0
ZBTB7C	Zinc finger and btb domain containing 7c	1675	381	0.23	Down	7.23E-169	4.55E-159
ZDHHC13	Zinc finger dhhc-type palmitoyltransferase 13	2501	1151	0.46	Down	1.79E-88	7.70E-79
ZNF185	Zinc finger protein 185	5565	1162	0.21	Down	0	0
ZNF296	Zinc finger protein 296	552	257	0.47	Down	2.14E-17	4.77E-07
ZNF335	Zinc finger protein 335	4179	1934	0.46	Down	6.59E-148	3.81E-138
ZNF385B	Zinc finger protein 385b	979	97	0.10	Down	3.31E-167	2.06E-156
ZNF436	Zinc finger protein 436	901	408	0.45	Down	4.74E-31	1.28E-21
ZNF532	Zinc finger protein 532	4851	1360	0.28	Down	0	0
ZNF710	Zinc finger protein 710	1525	517	0.34	Down	3.44E-95	1.54E-86
ZNF750	Zinc finger protein 750	11619	1733	0.15	Down	0	0
ZYG11B	Zyg-11 family member b. cell cycle regulator	859	397	0.46	Down	6.08E-28	1.59E-18
